# Dynamic carbon overlayered CuCo alloy promotes syngas to higher alcohols

**DOI:** 10.1038/s41467-026-78203-7

**Published:** 2026-09-30

**Authors:** Jia Liu, Si-Wei Ying, Jiachang Zuo, Qiuyue Zhang, Shan Gao, Xuelian Liang, Xinping Duan, Jing Ren, Yufei Zhao, Mingshu Chen, Linmin Ye, Su-Yuan Xie, Jian-Wei Zheng, Youzhu Yuan

**Affiliations:** 1 State Key Laboratory of Physical Chemistry of Solid Surfaces, College of Chemistry and Chemical Engineering, Xiamen University, Xiamen, China; 2 State Key Laboratory of Chemical Resource Engineering, Beijing University of Chemical Technology, Beijing, China

**Keywords:** Porous materials, Heterogeneous catalysis, Catalyst synthesis

## Abstract

Syngas (CO/H_2_), sourced from coal, biomass, and shale gas, plays a crucial role in producing a wide range of industrially essential hydrocarbons and oxygenates through Fischer–Tropsch synthesis and its variations. Despite extensive research dating back nearly a century, transforming syngas into higher alcohols remains challenging due to issues with low selectivity and catalyst instability. Generally, more than 50% of CO in typical syngas conversions yields unwanted hydrocarbons, mainly alkanes. Herein, we develop a catalyst integrating a Mo_2_C-matrix-supported CuCo alloy with hydrophobic graphitic carbon overlayers, achieving 80% selectivity toward value-added alcohols and olefins, of which 59% are higher alcohols, while limiting alkanes to around 10%. The overlayers dynamically participate in the reaction through carbon consumption and replenishment, generating carbon-overlayer-derived CH_*x*_ species that form an asymmetrically polarised coupling pair with CO-derived intermediates, thereby lowering the barrier for CO insertion and diverting CO from deoxygenation pathways leading to alkane and coke formation. Consequently, the yield of value-added products reaches four times that of typical benchmarks at 80% CO conversion with long-term stability. This work represents a substantial advance in higher alcohol synthesis and sheds new light on the catalytic role of surface carbon in syngas conversion.

## Abstract (web summary)

Converting syngas into higher alcohols (HAs) remains difficult because of limited selectivity and catalyst instability. Here, the authors develop a CuCo alloy–Mo_2_C catalyst with hydrophobic graphitic carbon overlayers that delivers 80% selectivity toward value-added alcohols and olefins, including 59% higher alcohols, while limiting alkane formation to about 10%.

## Introduction

Syngas (CO/H_2_), derived from coal, biomass, and shale gas, holds significant potential for its transformation into a wide array of industrially vital hydrocarbons and oxygenates through Fischer–Tropsch (F–T) synthesis and related processes^1–3^. Such conversions enable the production of essential compounds such as aromatics, acids, and olefins, as well as higher alcohols (HAs) with two or more carbon atoms (C_2+_OH), which are among the most promising clean alternative aviation fuels and support global sustainability objectives^4,5^. While large-scale commercialisation of hydrocarbon synthesis from syngas is underway, the direct and efficient production of HAs remains a complex challenge, plagued by low selectivity and catalyst instability, despite extensive research dating back nearly a century^6–8^. In principle, HA synthesis requires catalysts possessing dual functionalities for C–O bond breaking and retention, which traditional single-metal catalysts struggle to achieve^9,10^. Modified F–T synthesis catalysts, particularly CuCo-based systems, have emerged as promising candidates, with extensive research demonstrating their potential^11–14^. However, making significant breakthroughs remains a formidable challenge due to the difficulty in improving HA selectivity while maintaining long-term catalyst stability^15,16^. Excessive CO dissociation causes an imbalance between CH_*x*_ hydrogenation and CO insertion, leading to undesirable alkanes as the dominant products. Additionally, C–O bond cleavage promotes the formation of metal carbide species, causing CuCo phase separation due to the high carbon solubility of F–T-type metals. Moreover, surface CO dissociation can induce carbon deposition that blocks active sites, resulting in catalyst deactivation and deteriorated catalytic performance^17–19^. Therefore, mitigating the adverse effects associated with CO deoxygenation, especially alkane formation, is crucial for improving catalytic performance in syngas-to-HA conversion.

Here, we show a catalyst integrating a Mo_2_C-matrix-supported CuCo alloy with graphitic carbon overlayers (GCLs) through carburisation^20^. The presence of these GCLs markedly promotes HA yield and selectivity while suppressing alkane-forming side reactions and stabilising the CuCo alloy, thereby prolonging catalyst lifetime. Unlike conventional spectator carbon species, GCLs are unexpectedly active. Notably, the defective carbon sites at the CuCo–GCL interface generate GCL-derived CH_*x*_ species that directly couple with CO-derived intermediates to form an asymmetrically polarised C–C coupling pair and actively participate in the reaction cycle. This mechanism partially substitutes for traditional CO deoxygenation pathways, reducing excessive CO dissociation that typically leads to alkane formation. During the synthesis of HAs, the consumed carbon is replenished by CO, rendering the carbon species regenerable and retarding coke formation. Additionally, the hydrophobic properties of the GCLs protect the CuCo alloy from oxidation and inhibit the water-gas shift reaction (WGSR), thereby lowering CO_2_ selectivity.

## Results

### Structure evolution and catalytic performance

**Fig. 1:**
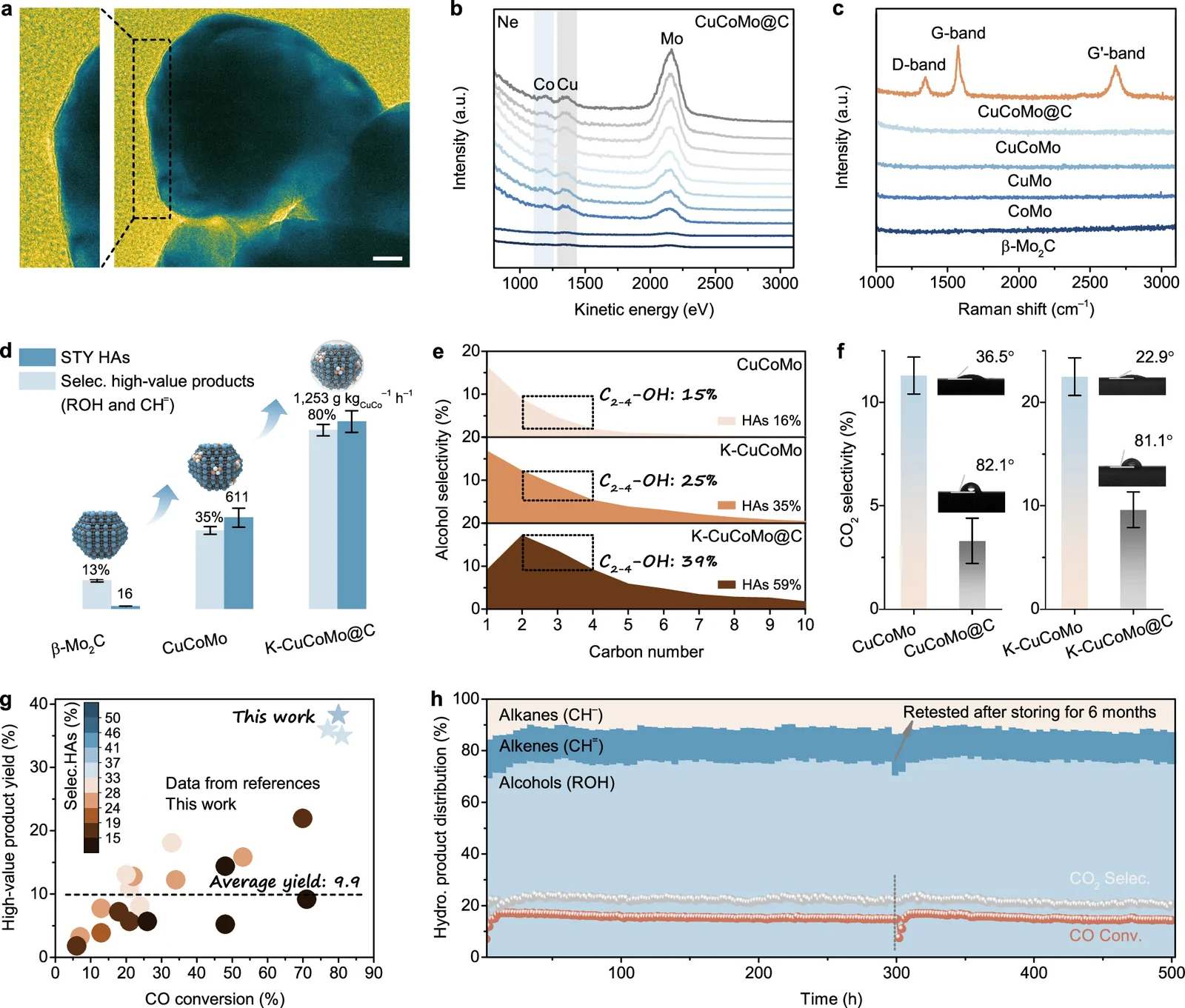
Structural characteristics and catalytic performance of the catalysts. **a** HRTEM images of CuCoMo@C. Scale bar, 20 nm. **b** Ne spectra from layer-by-layer etching LEIS of CuCoMo@C. **c** Raman spectra of different catalysts after carburisation. **d** Selectivity of high-value products and STY of HAs. **e** Detailed alcohol selectivity of different catalysts based on the carbon number. **f** CO_2_ selectivity and the contact angle. **g** Comparison of high-value product yield and HA selectivity with reported catalysts (refs. ^38–53^). This work: K-CuCoMo@C. **h** Time-on-stream (TOS) evolution of K-CuCoMo@C (GHSV = 4 L g_cat_^−1^ h^−1^). The spent catalyst after a 300 h test was stored for 6 months and further tested for another 200 h. Error bars represent standard deviations (SD). Reaction conditions: *T* = 240 °C, *P* = 3.0 MPa, GHSV = 8 L g_cat_^−1^ h^−1^, and H_2_/CO = 2. ROH = total alcohols. MeOH = methanol. HAs = higher alcohols. CH^−^ = alkanes (including CH_4_). CH^=^ = alkenes. RH = hydrocarbons (CH^−^ + CH^=^). High-value products = ROH + CH^=^. STY = space-time yield based on the CuCo weight. Yield = product yield on a carbon-number basis.

The precursor containing mixed oxides Co_*x*_Cu_1__-__*x*_MoO_4_^21^ was first prepared from aqueous metal salt solutions (Fig. S1a, b). After carburisation, the oxides are reduced to metallic Cu and Co. Carbon species derived from CH_4_ replace oxygen in the Mo matrix while simultaneously depositing on the surface as GCLs, yielding the CuCoMo@C catalyst (Fig. 1a). Notably, similar GCLs are not observed on the CuMo or CoMo samples (Fig. S1d). In situ X-ray diffraction (in situ XRD) was conducted to investigate the structural evolution during carburisation. Three major stages are identified along the transformation sequence MoO_3_ → MoO_2_ → β-Mo_2_C. CuMoO_4_, CoMoO_4_, and Co_*x*_Cu_1__-__*x*_MoO_4_ are gradually reduced to metallic Cu, Co, and a CuCo alloy, respectively (Fig. S2). Analysis of the lattice constants reveals that both Cu and Co lattice constants increase with temperature because of thermal expansion. For CuCoMo@C, Cu lattice constants remain between those of CuMo and CoMo over the temperature range of 625–700 °C, indicating the formation of a CuCo alloy (Fig. S1c). Energy-dispersive X-ray spectroscopy (EDS) line-scan and elemental mapping show that Cu and Co exhibit identical spatial distributions and are concentrated in the particle interior, where a CuCo alloy with a lattice spacing of 2.10 Å is observed. Carbon is enriched at the surface, confirming the presence of GCLs. Mo_2_C with a lattice spacing of 2.28 Å surrounds the CuCo alloy particles (Fig. S3).

By controlling the sputtering energy, the first two scans of high-sensitivity low-energy ion scattering (HS–LEIS) spectra show negligible signals for Cu, Co, and Mo species, indicating that carbon species cover the catalyst surface (Fig. 1b). During Ne^+^ sputtering, the removal of carbon species is accompanied by the synchronous appearance of Cu and Co signals, suggesting that Cu and Co species exist beneath the GCLs. Figure 1c shows that only the CuCoMo@C sample presents Raman bands of carbon^22^. The G-band at 1578.4 cm^−1^ and G’-band at 2686.9 cm^−1^ are characteristic of a highly graphitised structure. The appearance of D-band at 1346.2 cm^−1^ confirms the existence of defective carbon. These results indicate that the CuCo alloy, supported by Mo_2_C, is covered by GCLs. Extended X-ray absorption fine structure (EXAFS) spectra at the Cu K-edge confirm that Cu remains in the metallic state, with both catalysts showing a similar peak at 2.22 Å for Cu–Cu(Co) coordination. However, CuCoMo and CuCoMo@C exhibit quite different Co K-edge spectra. Co–O coordination is observed only for CuCoMo, whereas CuCoMo@C contains Co–Co(Cu) coordination exclusively, demonstrating that GCLs protect the CuCo alloy from oxidation (Fig. S4). Notably, the peak shifts to a lower R distance compared with the Co foil, which may result from the altered Co coordination environment induced by surrounding Mo and C atoms, leading to enhanced interfacial interactions.

In CO hydrogenation, Mo_2_C demonstrates a strong capability to convert CO^23,24^, with dissociated CO undergoing deep hydrogenation to generate a high selectivity of 78.7% for hydrocarbons at 240 °C. The addition of Cu and Co to Mo_2_C enhances alcohol production, but the F–T reaction remains dominant, resulting in 56.5% hydrocarbon selectivity, with alkanes accounting for 53.9% (Table S1). The rapid alkane synthesis reaction suppresses CO conversion toward alcohols. Potassium promotion (K-CuCoMo) increases the selectivity toward HAs to 35.3%, yet the value remains insufficient (Fig. S5a–d). However, the presence of GCLs (K-CuCoMo@C) significantly enhances the selectivity of HAs to 58.9% and promotes CO conversion, resulting in a 2.1-fold improvement in STY of HAs (Fig. 1d). Concurrently, the hydrocarbon selectivity is reduced to 22.7%. These results suggest that CH_*x*_ hydrogenation is inhibited, while the CO insertion process is enhanced by surface GCLs. Among alcohol products with different carbon numbers, GCLs markedly suppress methanol formation and increase C_2–4_–OH selectivity from 15% of CuCoMo and 25% of K-CuCoMo to 39% of K-CuCoMo@C (Fig. 1e). Meanwhile, in kinetic tests, the K-CuCoMo@C catalysts exhibit a deviation from the linear Anderson–Schulz–Flory (ASF) distribution, particularly for short-chain C_2_–C_4_ alcohols (Fig. S6). These results suggest that the formation of HAs involves external CH_*x*_ monomers that are influenced by the GCLs. Additionally, the GCLs increase the proportion of olefins in hydrocarbon products from 4.6% to 52.0%, enabling the co-production of olefins and alcohols. The selectivity for high-value products reaches 80%, with alkanes maintaining a low selectivity of nearly 10% (Fig. S7a). Moreover, the incorporation of Mo and K increases CO_2_ selectivity. However, increasing the thickness of GCLs enhances hydrophobicity, as evidenced by the larger contact angle (Fig. S8). The hydrophobic nature isolates water produced during hydrogenation, inhibiting the side WGSR and reducing CO_2_ production. As a result, the CO_2_ selectivity decreases to below 10% after carbon coating (Fig. 1f).

Subjecting the K-CuCoMo@C catalyst to reactions at 230–290 °C shows that increasing the temperature inhibits methanol formation while enhancing the production of C_2+_OH, reaching a distribution up to 91.7%. CO_2_ production does not increase significantly due to the hydrophobic nature of the GCLs. The yield of HAs reaches its maximum at 280 °C, while an increase in pressure further enhances CO conversion, resulting in the highest STY of 3,201 g kg_CuCo_^−1^ h^−1^ at a CO conversion of 80% (Figs. S5e, f and S7b). Compared to the commercial CuZnAl catalyst, K-CuCoMo@C demonstrates nearly triple the reaction rate (Fig. S7c). The catalyst coated with GCLs exhibits hydrocarbon selectivity far below the typical reported average of 56%, effectively inhibiting the formation of undesired alkanes and substantially enhancing the yield of HAs (Fig. S9). Consequently, this catalyst delivers a remarkable yield of high-value products, approximately four times higher than that of most catalysts reported in the literature (Fig. 1g and Table S2). The (K-)CuCoMo@C catalysts show excellent stability, maintaining their performance for over 300 h of continuous operation. Even after six months of storage, the catalytic performance remains robust during a 200 h test (Fig. 1h). XRD patterns of the (K-)CuCoMo@C catalysts before and after reaction show negligible changes, with no discernible diffraction peaks associated with Co_*x*_C, confirming that CuCo species can retain their metallic state due to the protective GCLs. In contrast, the CuCoMo catalyst in the absence of GCLs exhibits unstable catalytic performance, with XRD patterns showing reduced peak intensity for CuCo species (Fig. S10a–e). Compared with the fresh catalyst, the Raman spectrum of the spent CuCoMo catalyst shows evident D and G bands, confirming the formation of carbon deposits during the reaction (Fig. S10f–h). This result suggests that the reconstruction of the GCLs on the surface leads to the redispersion of Cu and Co metals. The reduced metal particle size enhances the selectivity toward hydrocarbons.

### CuCo–GCL interface and dynamic carbon mobility

**Fig. 2:**
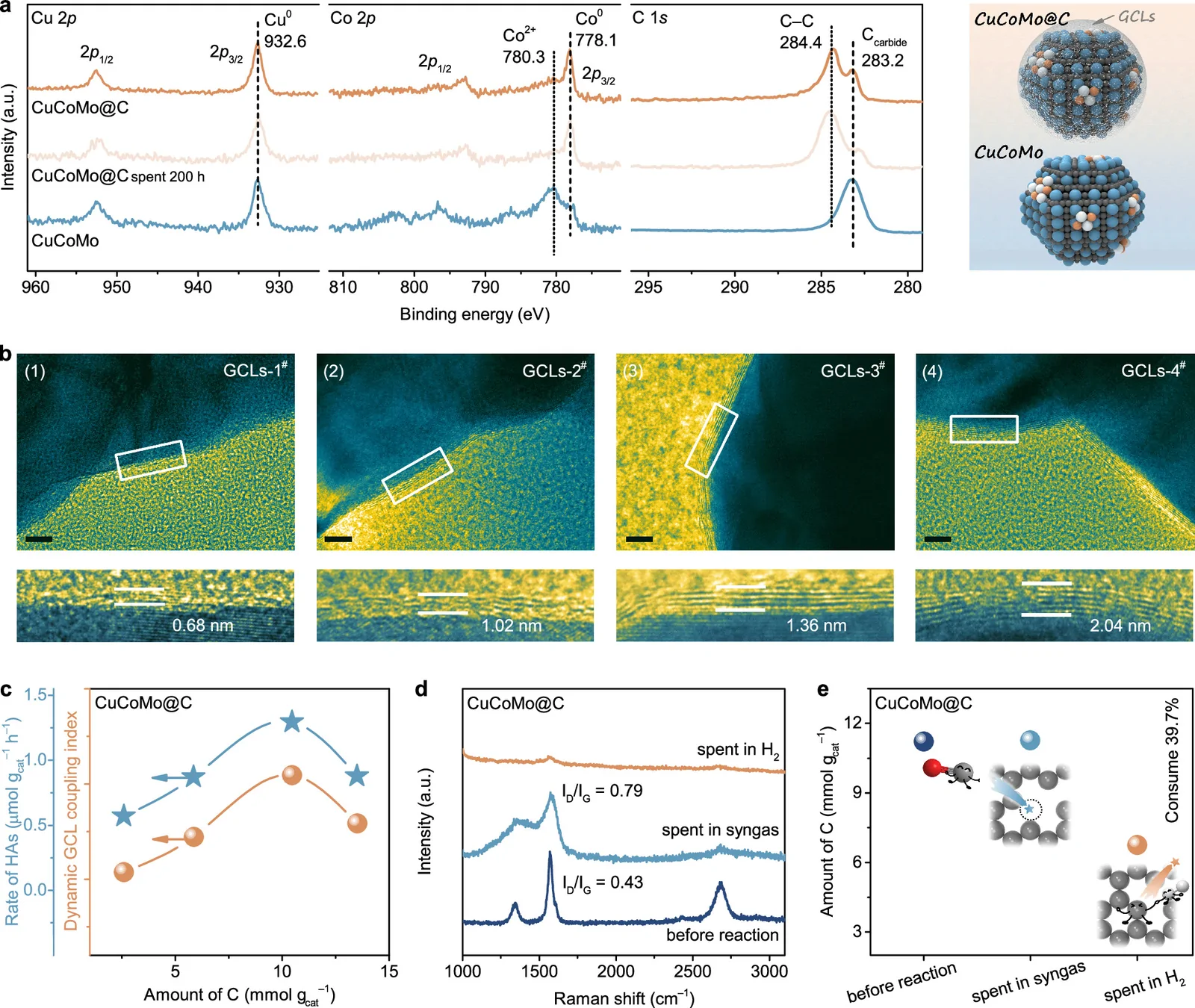
Effects of the GCLs on catalytic performance. **a** Ar-ion etching XPS spectra (50 s) of fresh CuCoMo@C, spent CuCoMo@C (200 h), and fresh CuCoMo. **b** HRTEM images of CuCoMo@C with different carbon amounts (GCLs-1^#^ to GCLs-4^#^). Scale bar, 5 nm. **c** Correlation between the HA formation rate and the dynamic GCL coupling index (DGCI) as a function of carbon amount. DGCI serves as a semi-quantitative descriptor of the combined contributions of GCL-regulated intermediate-generation and C–C coupling sites, defined as DGCI = CuCo–GCL_def-int_ × CuCo–GCL_mod_, where CuCo–GCL_def-int_ is estimated as CuCo–GCL_int_ × I_D_/I_G_. CuCo–GCL_int_: CuCo–GCL interfacial sites, identified from moderately adsorbed CO species. CuCo–GCL_def-int_: defective interfacial carbon sites at the CuCo–GCL interface. CuCo–GCL_mod_: GCL-modified CuCo sites, associated with strongly adsorbed CO species. **d** Raman spectra of CuCoMo@C before reaction, spent in syngas, and spent in H_2_. **e** Carbon amounts of CuCoMo@C before reaction, spent in syngas, and spent in H_2_ calculated from O_2_–TPO. Reaction conditions: GHSV = 4 L g_cat_^−1^ h^−1^, *T* = 240 °C, *P* = 3.0 MPa, under syngas (H_2_/CO = 2) or pure H_2_.

Ar-ion etching X-ray photoelectron spectroscopy (XPS) was employed to examine the effects of GCL coverage (Fig. 2a). In the CuCoMo catalyst, carbon species are primarily present as Mo_2_C, Cu remains metallic, whereas Co is mainly in the +2 oxidation state, likely owing to air oxidation prior to XPS measurement (Fig. S11a). In contrast, for CuCoMo@C before and after 200 h of reaction, the binding energies of the Cu 2*p* and Co 2*p* peaks remain essentially unchanged and are consistent with the metallic state, indicating that the CuCo alloy is stable even after exposure to air. The C 1*s* spectra show two carbon species assigned to graphitic carbon and carbide, confirming the coexistence of GCLs and the Mo_2_C matrix (Fig. S11b, c). These results prove that the presence of GCLs stabilises the bimetallic CuCo alloy against oxidation and phase separation^25^. High-resolution transmission electron microscopy (HRTEM) images show that four CuCoMo@C catalysts with GCL thicknesses ranging from 0.68 to 2.04 nm, corresponding to approximately 1–6 graphitic carbon layers, were prepared by controlling the carburisation conditions (Fig. 2b). The carbon amount was quantified from O_2_–temperature-programmed oxidation (O_2_–TPO) by monitoring O_2_ consumption using the m/z = 32 signal, revealing a gradual increase in carbon amount with increasing GCL thickness (Fig. S12a, b). Raman spectra show that at a moderate carbon amount, the elevated I_D_/I_G_ ratio indicates a higher density of carbon defects (Fig. S12c).

To further clarify the structure of the GCL-covered catalyst surface, CO chemisorption was employed to probe the number and nature of accessible adsorption sites. The results show that substantial CO uptake is retained even after the formation of GCLs, indicating that the GCLs do not completely block the metallic surface, but instead create more adsorption environments. More importantly, as the carbon amount and defect density vary, the adsorption properties of these sites evolve systematically, suggesting that the GCLs are not merely spectator species, but actively regulate the surface structure (Fig. S13a). Based on their adsorption behaviour, two major types of CO species can be distinguished. The first corresponds to moderately adsorbed CO species, reasonably assigned to CuCo–GCL interfacial sites (CuCo–GCL_int_), including defective interfacial carbon sites. The second corresponds to strongly adsorbed CO species, attributed to GCL-modified CuCo sites (CuCo–GCL_mod_), which remain accessible for CO/H_2_ activation and generate CO-derived intermediates (Fig. S13b). Notably, when the abundance of these quantified interfacial sites is incorporated into the GCL coupling index, its dependence on carbon amount follows a volcano-type trend that closely matches the selectivity and formation rate of HAs (Figs. 2c and S13c). This strong correlation suggests that the catalytically relevant structure originates from the synergistic CuCo–GCL interface, rather than bare CuCo surfaces or isolated GCLs alone. Moderate carbon deposition optimises the effective metal–GCL interfacial structure, thereby promoting HA synthesis and leading to a prolonged induction period. In contrast, excessive carbon coverage reduces the defective interfacial sites, lowers metal accessibility, suppresses alcohol formation, and enables the reaction to reach equilibrium more rapidly (Fig. S12d). In addition, post-reaction Raman spectra reveal an increase in carbon defect density within the GCLs, accompanied by pronounced structural evolution (Fig. 2d). Together, these results suggest that defective interfacial carbon sites at the CuCo–GCL interface (CuCo–GCL_def-int_) serve as the active centres for C–C coupling, while the GCLs likely undergo dynamic structural evolution under reaction conditions.

The dynamic evolution of GCLs during the reaction is crucial for HA formation. To understand the process, CuCoMo@C was sequentially subjected to reactions in pure H_2_ and syngas. As shown in Fig. 2d, e, after H_2_ treatment, the carbon amount decreases, the Raman bands associated with GCLs are significantly weakened, and hydrocarbon products are detected by chromatography. TPO profiles indicate that the amount of consumed carbon in CuCoMo@C is much larger than that in CuCo@C under identical reaction conditions (Fig. S14a–d). The enhanced hydrogenation of GCLs supported by the Mo_2_C matrix, compared with metallic substrates, can be attributed to the interstitial carbon atoms in Mo_*x*_C^26^, which promote the mobility of GCLs (Fig. S15), together with enhanced electron donation from Mo_2_C to the GCLs, leading to electron enrichment of interfacial carbon species and increased reactivity toward hydrogenation (Fig. S14e, f). Consequently, the GCLs in CuCoMo@C readily generate reactive CH_*x*_ intermediates that participate in the reaction cycle. After the syngas reaction, the carbon amount of CuCoMo@C remains stable, but the structure of GCLs changes. The G’-band intensity decreases, while the D-band becomes broader, showing the generation of additional carbon defect sites and replenishment of consumed GCLs during CO hydrogenation. HS–LEIS and HRTEM further verify this dynamic process. After H_2_ treatment, the first HS–LEIS scan reveals characteristic Cu, Co, and Mo signals, confirming the consumption of surface carbon species (Fig. S16a, b). However, after the syngas reaction, the metallic signals remain weak in the first four HS–LEIS scans, demonstrating the regeneration of GCLs from CO. HRTEM images show that the replenished GCLs exhibit a more disordered and thicker structure than the initial GCLs (Fig. S16c, d). To substantiate the carbon consumption and replenishment process, near-ambient-pressure X-ray photoelectron spectroscopy (NAP–XPS) was conducted by alternately switching between H_2_ and CO every 15 min (Fig. S16e). Adsorbed CO species appear under CO but decrease under H_2_. Interestingly, the integrated peak area of C–C bonded carbon species decreases under H_2_ and returns to the original ratio after switching back to CO (Fig. S16f), consistent with the dynamic reconstruction of GCLs.

### Dynamic GCL reconstruction through carbon exchange

**Fig. 3:**
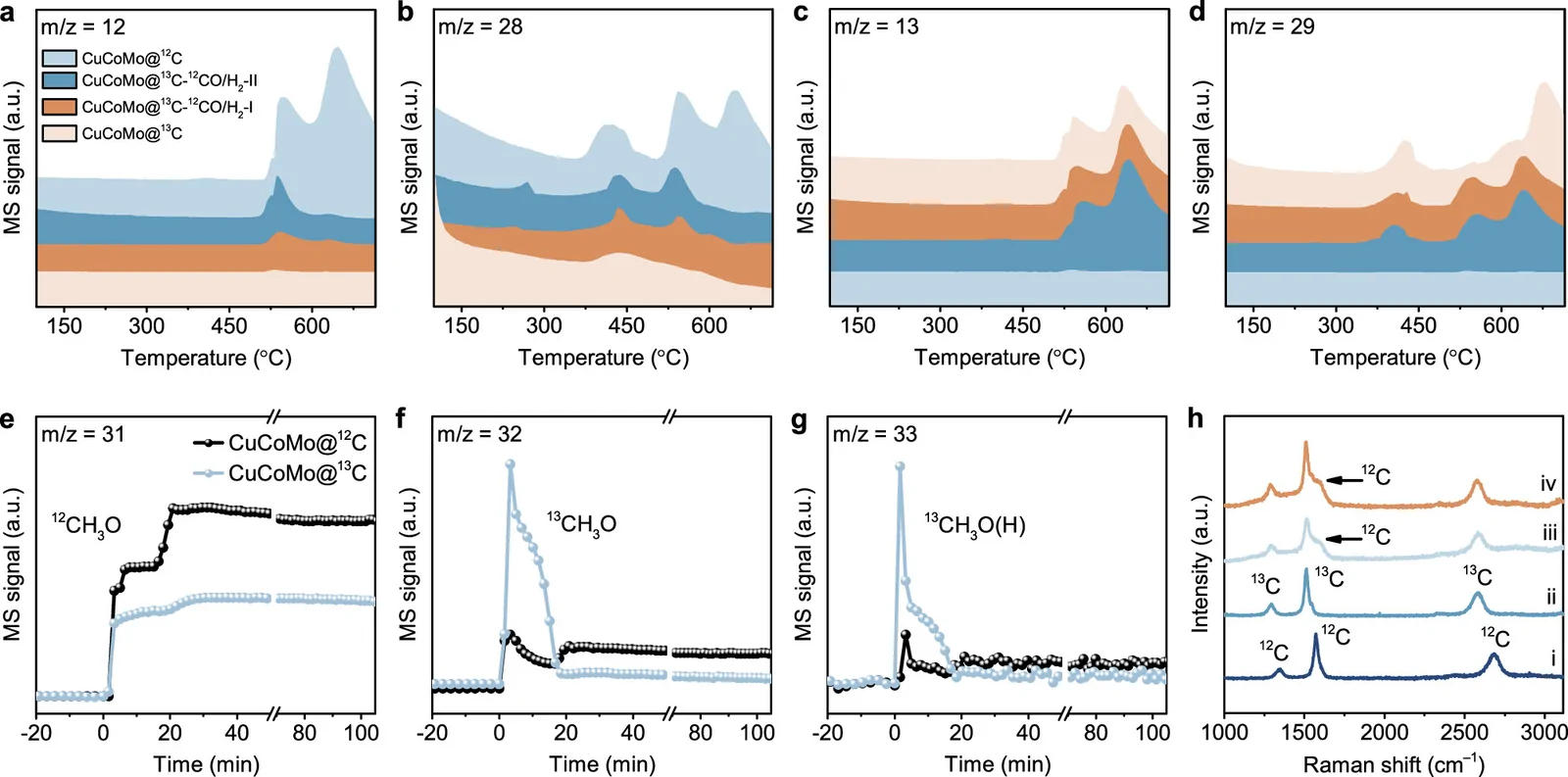
Isotope tracing of reaction processes. **a–d** O_2_–TPO profiles of CuCoMo@^12^C, CuCoMo@^13^C, and syngas-spent CuCoMo@^13^C after reaction durations I and II, with the corresponding MS signals (m/z = 12, 28, 13, and 29). **e–g** TPSR profiles of CuCoMo@^12^C and CuCoMo@^13^C, with the corresponding MS signals (m/z = 31, 32, and 33). **h** Raman spectra of the catalysts. (i) CuCoMo@^12^C. (ii) CuCoMo@^13^C. (iii and iv) syngas-spent CuCoMo@^13^C after reaction durations I and II. The reaction duration in II is approximately twice that in I. Reaction conditions: GHSV = 8 L g_cat_^−1^ h^−1^, *T* = 240 °C, *P* = 3.0 MPa, H_2_/^12^CO = 2.

The isotope-labelling study was conducted to verify that carbon species in the GCLs are dynamic and directly participate in CO hydrogenation. CuCoMo@^13^C was prepared using ^13^CH_4_/H_2_ as the carburisation source and subsequently subjected to reaction in syngas (^12^CO/H_2_) at 240 °C for two reaction durations. O_2_–TPO coupled with a mass spectrometer (MS) was then performed on these fresh and syngas-spent catalysts. The m/z = 12 and 28 signals are negligible for CuCoMo@^13^C but intense for CuCoMo@^12^C (Fig. 3a, b), confirming the successful preparation of the isotopically labelled catalyst. Meanwhile, both syngas-spent CuCoMo@^13^C catalysts exhibit appreciable m/z = 12 and 28 intensities, indicating that carbon derived from ^12^CO is incorporated into the GCLs. Conversely, the m/z = 13 and 29 signals are strongest for CuCoMo@^13^C but negligible for CuCoMo@^12^C (Fig. 3c, d), whereas both syngas-spent CuCoMo@^13^C catalysts display diminished intensities, demonstrating the simultaneous consumption of the original ^13^C species in the GCLs. Together, these results show that the GCLs are consumed and replenished during CO hydrogenation. Based on the m/z = 28 and 29 signals, the carbon was quantified at different extents of GCL consumption (Fig. S17). It reveals a progressive decrease in ^13^C, accompanied by a corresponding increase in ^12^C, while the total carbon (^12^C + ^13^C) remains essentially constant, suggesting a sustained dynamic GCL reconstruction cycle during CO conversion.

The dynamic process is further corroborated by temperature-programmed surface reaction (TPSR) measurements (Figs. 3e–g and S18). Product fragments with m/z = 14, 15, 16, 17, 18, 28, 29, 30, 31, 32, 33, 44, 45, and 46 were collected. For most fragments, the signal intensities of CuCoMo@^12^C are higher than those of CuCoMo@^13^C. In contrast, the m/z = 32 (^13^CH_3_O) and 33 (^13^CH_3_O(H)) originating from ^13^C-containing alcohols show higher intensities for CuCoMo@^13^C than for CuCoMo@^12^C, indicating that carbon species directly participate in alcohol formation. These results provide compelling evidence that the GCLs are hydrogenated to generate GCL-derived CH_*x*_ species, which subsequently couple with CO-derived intermediates to form alcohols, thereby partially substituting the conventional CO deoxygenation pathway. Compared with CuCoMo@^12^C, all Raman bands associated with the GCLs are shifted to lower wavenumbers in CuCoMo@^13^C owing to the isotopic mass effect. The D-band shifts from 1341 cm^−1^ to 1290 cm^−1^, the G-band shifts from 1570 cm^−1^ to 1514 cm^−1^, and the G’-band shifts from 2677 cm^−1^ to 2584 cm^−1^ (Fig. 3h). For both syngas-spent CuCoMo@^13^C catalysts, two distinct G-band peaks are observed at 1512 cm^−1^ and 1577 cm^−1^, corresponding to ^13^C- and ^12^C-containing graphitic carbon, respectively. The Raman results confirm that the carbon from CO is incorporated into the graphitic framework of the GCLs, rather than being physically deposited on the catalyst surface.

### Mechanism of dynamic GCL-involved alcohol synthesis

**Fig. 4:**
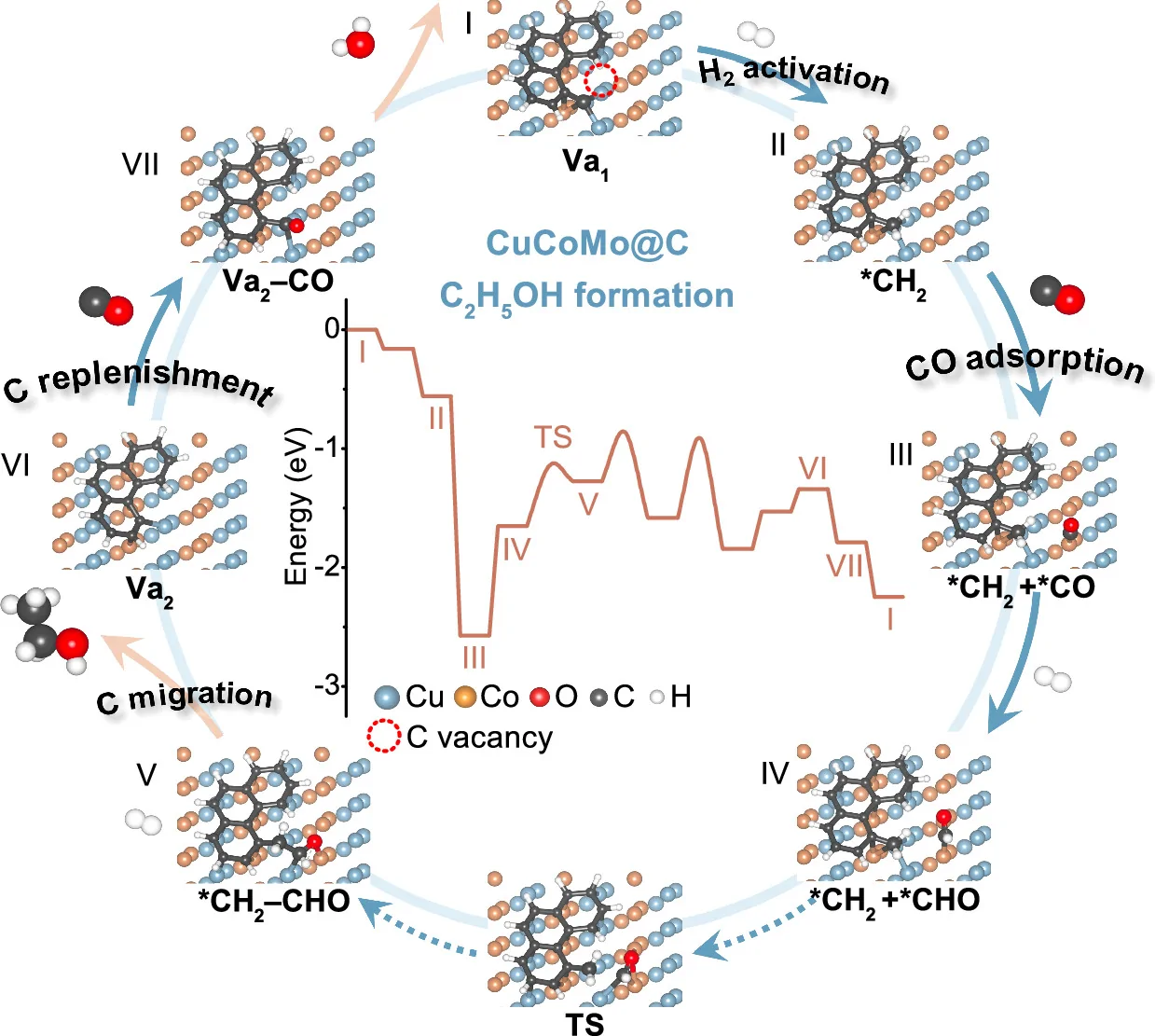
Dynamic GCL participation mechanism. Reaction pathway for ethanol formation over CuCoMo@C.

To gain mechanistic insight into the dynamic participation of GCLs, density functional theory (DFT) calculations were performed. As shown in Fig. 4, the reaction on CuCoMo@C is initiated by hydrogenation of defective interfacial carbon species at the CuCo–GCL interface, generating GCL-derived *CH_2_ species. Meanwhile, CO adsorbed on adjacent CuCo sites is hydrogenated to form CO-derived *CHO intermediate. The co-adsorbed *CH_2_/*CHO subsequently undergo C–C coupling to form *CH_2_–CHO, followed by successive hydrogenation to *CH_2_–CH_2_O, *CH_3_–CH_2_O and finally C_2_H_5_OH (Fig. S19a, b). On the CuCoMo surface, all intermediates originate exclusively from CO activation, and the selectivity toward C_2_ products is determined by the competition between *CH_2_/*CHO and *CH_2_/*CH_2_ coupling, which constitute the highest kinetic barriers for oxygenate and hydrocarbon formation, respectively (Fig. S20)^11,27^. The transition-state (TS) barrier for *CH_2_/*CHO coupling (1.29 eV) is higher than that for *CH_2_/*CH_2_ coupling (1.02 eV), favouring alkane formation. Remarkably, the participation of GCLs fundamentally alters this competition. On CuCoMo@C, the barrier for *CH_2_/*CHO coupling is substantially reduced to 0.51 eV, whereas that for *CH_2_/*CH_2_ coupling increases to 1.16 eV, making *CH_2_–CHO the preferred C–C coupling intermediate (Fig. S21). Compared with the bare metal surface, direct *CH_2_–CH_2_ desorption is thermodynamically more favourable in the presence of the GCLs, further suppressing alkane formation (Fig. S22). The slightly increased barrier is associated with the extraction of a GCL carbon atom during the late-stage hydrogenation step, reflecting the dynamic participation of the GCLs. Upon C_2_H_5_OH desorption, the resulting interfacial carbon vacancy is replenished by gaseous CO^28^, thereby completing the dynamic carbon reconstruction cycle.

To elucidate the origin of the preferential *CH_2_–CHO coupling, charge density difference and Bader charge analyses were performed. Compared with CO-derived *CH_2_^CO^, the GCL-derived *CH_2_^GCL^ possesses a less electron-rich carbon centre owing to charge redistribution at the CuCo–GCL interface (Fig. S23a). On the CuCoMo surface, co-adsorbed *CH_2_^CO^/*CH_2_^CO^ and *CH_2_^CO^/*CHO^CO^ pairs exhibit nearly symmetric charge distributions, with charge differences (|Δq|) of only 0.007 and 0.01 e, respectively. In contrast, on CuCoMo@C, co-adsorbed GCL-derived *CH_2_^GCL^ with CO-derived *CH_2_^CO^ and *CHO^CO^ display much larger charge differences of 0.25 and 0.37 e, respectively, forming asymmetrically polarised coupling pairs (Fig. S23b, c). Although both coupling pairs become electronically polarised, the greater charge polarisation and the higher intrinsic electrophilicity of the carbonyl carbon render *CH_2_^GCL^/*CHO^CO^ coupling kinetically more favourable than *CH_2_^GCL^/*CH_2_^CO^ coupling (Fig. S19c, d). The pronounced asymmetric charge polarisation enhances the electronic complementarity between the coupling partners and selectively lowers the CO insertion barrier^29^. Therefore, GCL-derived CH_*x*_ species act as carbon donors, partially substituting CH_*x*_ species generated through CO deoxygenation, while electronically regulating the C–C coupling process and redirecting CO hydrogenation toward alcohol formation. Furthermore, K promotion slightly strengthens CO adsorption (Fig. S24), increasing the surface coverage of CO-derived intermediates and thereby further facilitating the coupling with GCL-derived CH_*x*_ species^30^, consistent with the experimentally observed enhancement in HA formation.

## Discussion

By constructing a GCL-coated CuCo alloy supported on a Mo_2_C matrix through in situ carburisation, alkane selectivity is successfully suppressed to 10%, enabling the co-production of alcohols and olefins with an overall 80% selectivity. In particular, the selectivity toward HAs is increased to nearly 60%, maintaining long-term stability even after prolonged storage. The present results reveal that defective carbon sites at the CuCo–GCL interface are closely correlated with HA formation. The GCLs are not merely passive structural coatings but active catalytic components that dynamically participate in CO hydrogenation. Unlike conventional F–T catalysts, where all intermediates originate exclusively from CO dissociation, the GCLs undergo hydrogenation to generate GCL-derived CH_*x*_ species that serve as carbon donors for HA formation. Together with remediation by gaseous CO, this establishes a sustained carbon consumption-replenishment process, partially substituting the CO deoxygenation pathway. Beyond acting as carbon reservoirs, the GCLs also regulate the electronic structure of the C–C coupling intermediates. GCL-derived CH_*x*_ species form asymmetrically polarised coupling pairs with CO-derived intermediates, preferentially promoting *CH_*x*_ and *CHO coupling while inhibiting the competing hydrocarbon pathway. Consequently, the CO insertion barrier is substantially lowered, thereby promoting oxygenate formation. In addition, the GCLs stabilise the CuCo alloy against oxidation and phase separation while reducing CO_2_ formation through their hydrophobic nature, highlighting their multifunctional role in catalyst stabilisation and product regulation. This dynamic GCL-involved mechanism provides a solution to the long-standing trade-off between catalytic activity and oxygenate selectivity, extending the traditional understanding of metal sites and surface carbon species. These findings demonstrate a general strategy for engineering dynamic catalyst surfaces to steer C–C coupling pathways and enhance oxygenate selectivity in syngas conversion.

## Methods

### Synthesis of catalysts

Preparation of the CuCoMo catalyst: ammonium molybdate ((NH_4_)_6_Mo_7_O_24_·4H_2_O) was calcined at 500 °C for 4 h to obtain MoO_3_. The resulting MoO_3_ was subsequently carburised in 15% CH_4_/H_2_ (flow rate: 150 mL min^−1^) to get β-Mo_2_C. Then, 0.506 g of copper nitrate (Cu(NO_3_)_2_·3H_2_O) and 0.610 g of cobalt nitrate (Co(NO_3_)_2_·6H_2_O) were impregnated onto 2.310 g of Mo_2_C, followed by sonication for 1 h. The mixture was placed in a vacuum drying oven at 30 °C for 10 h, then gradually heated to 80 °C and kept overnight. The resulting solid was transferred to a tubular furnace and heated at 500 °C for 4 h under 5% H_2_/N_2_ (flow rate: 50 mL min^−1^). After cooling to room temperature, the gas was switched to 15% CH_4_/H_2_ (flow rate: 100 mL min^−1^), and the temperature was increased to 590 °C for 2 h. Finally, the sample was passivated using 1% O_2_/N_2_. The catalyst prepared is denoted as CuCo-Mo_2_C (abbreviated as CuCoMo).

Preparation of the CuCoMo@C catalyst: 4.0 g of ammonium molybdate ((NH_4_)_6_Mo_7_O_24_·4H_2_O), 0.506 g of copper nitrate (Cu(NO_3_)_2_·3H_2_O), and 0.610 g of cobalt nitrate (Co(NO_3_)_2_·6H_2_O) were dissolved in deionised water and stirred for 4 h. The mixture was then transferred to an oil bath and further stirred at 90 °C to form a thick slurry. After drying overnight, the solid was calcined in a muffle furnace at 500 °C for 4 h to obtain the oxide precursor. The carbide was synthesised via a temperature-programmed reduction method. The precursor was placed in a tubular furnace and heated to 700 °C at a ramp rate of 5 °C min^−1^ for 2 h under 15% CH_4_/H_2_ (flow rate: 150 mL min^−1^). After cooling to room temperature, the sample was passivated in 1% O_2_/N_2_. The resulting catalyst was denoted as CuCo-Mo_2_C@C (abbreviated as CuCoMo@C), with a metal loading of 10 wt% and a Cu:Co molar ratio of 1:1. For comparison, both Cu-Mo_2_C (CuMo) and Co-Mo_2_C (CoMo) were prepared with a metal loading of 5 wt%.

Preparation of the K-CuCoMo catalyst: 4.0 g of ammonium molybdate ((NH_4_)_6_Mo_7_O_24_·4H_2_O), 0.506 g of copper nitrate (Cu(NO_3_)_2_·3H_2_O), 0.610 g of cobalt nitrate (Co(NO_3_)_2_·6H_2_O), and 0.014 g of potassium carbonate (K_2_CO_3_) were dissolved in deionised water and stirred for 4 h. The subsequent steps followed the same procedure as that used for CuCoMo@C. The final catalyst was denoted as K-CuCo-Mo_2_C (abbreviated as K-CuCoMo), with a potassium (K) loading of 0.3 wt% as described in the main text.

Preparation of the K-CuCoMo@C catalyst: 4.0 g of ammonium molybdate ((NH_4_)_6_Mo_7_O_24_·4H_2_O), 0.506 g of copper nitrate (Cu(NO_3_)_2_·3H_2_O), 0.610 g of cobalt nitrate (Co(NO_3_)_2_·6H_2_O), 0.014 g of potassium carbonate (K_2_CO_3_), and 0.5 g of glucose were dissolved in deionised water and stirred for 4 h. The mixture was then transferred to an oil bath and continuously stirred at 90 °C to form a thick slurry. After drying overnight, the solid was calcined in a tubular furnace at 500 °C for 4 h under N_2_ (flow rate: 50 mL min^−1^). Subsequently, the precursor was carburised in 15% CH_4_/H_2_ (flow rate: 150 mL min^−1^) by heating to 700 °C for 2 h. After cooling, the sample was passivated in 1% O_2_/N_2_. The resulting catalyst was denoted as K-CuCo-Mo_2_C@C (abbreviated as K-CuCoMo@C). The potassium (K) loading was 0.3 wt% as mentioned in the text.

### Catalyst characterisation

X-ray powder diffraction (XRD) was carried out on the multifunctional diffractometer Smartlab-SE of Rigaku Corporation, using Cu K_α_ (λ = 0.15418 nm) as the radiation source. X-ray fluorescence mode was adopted at 40 kV and 30 mA.

In situ X-ray powder diffraction (in situ XRD) was performed using the same equipment. Typically, the precursor of the CuCoMo@C catalyst was placed in a high-temperature tank, and carburisation was carried out under 15% CH_4_/H_2_ with a heating rate of 1 °C min^−1^.

Transmission electron microscopy (TEM) and high-angle annular dark-field scanning TEM (HAADF–STEM) with energy dispersive spectroscopy (EDS) were performed on Philips Analytical FEI Tecnai 20 at an acceleration voltage of 200 kV. After grinding the catalyst, ethanol was added prior to ultrasonic treatment, and then dropped onto the grid.

O_2_–temperature-programmed oxidation (O_2_–TPO) measurements were carried out using a Micromeritics AutoChem II 2920 instrument connected with a mass spectrometer (MS). Typically, 35 mg of CuCoMo@C catalyst was weighed and pretreated at 300 °C for 1 h under a He atmosphere. After cooling to 50 °C, the gas was switched to 3% O_2_/He. The temperature was then increased to 800 °C at a ramping rate of 5 °C min^−1^, while monitoring the m/z = 32 signal using the MS. For the isotopic experiment, typically, 35 mg of the CuCoMo@^13^C catalyst was used. The signals at m/z = 28, 29, 12, and 13 were recorded, corresponding to ^12^CO, ^13^CO, ^12^C, and ^13^C, respectively.

CO–temperature-programmed desorption (CO–TPD) was performed using a Micromeritics AutoChem II 2920 instrument connected with an MS. Typically, 50 mg of CuCoMo@C catalyst was weighed and activated under 15% CH_4_/H_2_ at 590 °C for 2 h. After cooling to 50 °C, the sample was purged with He for 30 min. Subsequently, CO was introduced for 2 h, followed by He purging for 30 min. The temperature was then increased to 800 °C at a ramping rate of 10 °C min^−1^, while the signal at m/z = 28 was monitored.

Temperature-programmed surface reaction (TPSR) for the isotope experiment was performed using a Micromeritics AutoChem II 2920 instrument connected to an MS. Typically, 50 mg of the CuCoMo@^13^C catalyst was weighed and pretreated at 300 °C for 1 h under He atmosphere. The temperature was then lowered to 240 °C, and the gas was switched to ^12^CO/H_2_/N_2_ for 5 h. During this process, the MS was used to monitor signals at m/z = 14, 15, 16, 17, 18, 28, 29, 30, 31, 32, 33, 44, 45, and 46.

Raman spectroscopy was performed on a Renishaw RT 1000 Raman spectrometer. The excitation wavelength was 532 nm with the power of 5 mW.

High-sensitivity low-energy ion scattering (HS–LEIS) experiments were conducted using a QTAC^100^ instrument from the German IONTOF company. Either 3 keV ^4^He^+^ with an ion current density of 1325 pA cm^−2^ or 5 keV ^20^Ne^+^ with an ion current density of 445 pA cm^−2^ was used to probe the catalyst surface. To analyse surface elements such as Co, Cu, and Mo, ^20^Ne^+^ was selected as the ion source. The sputtering spot sizes were sequentially set at 2000 × 2000 μm (2 times), 1000 × 1000 μm (2 times), 500 × 500 μm (3 times), and 250 × 250 μm (3 times) to investigate the elemental information beneath carbon overlayers.

Ar-ion etching X-ray photoelectron spectroscopy (XPS) measurements were performed using a Thermo Fisher Escalab Xi+ instrument equipped with an Ar-ion gun for ion etching. Al K_α_ (*hv* = 1486.6 eV) was used as the X-ray radiation source. The etching rate was calculated to be 0.1915 nm s^−1^ based on the reference etching rate of Ta_2_O_5_.

Near-ambient-pressure X-ray photoelectron spectroscopy (NAP–XPS) measurements were performed using a SPECS system equipped with a differentially pumped Phoibos hemispherical electron energy analyser and monochromatic Al K_α_ radiation (*hv* = 1486.6 eV). The CuCoMo@C catalyst was activated by 15% CH_4_/H_2_ at 590 °C for 1 h, after which the temperature was decreased to 240 °C. Subsequently, H_2_, CO, H_2_, and CO were alternately introduced into the chamber for 15 min each at a pressure of 0.4 mbar.

Extended X-ray absorption fine structure (EXAFS) spectra were collected at the Beijing Synchrotron Radiation Facility (BSRF), using the 1W1B beamline.

The contact angle test was conducted using the contact angle measurement instrument DSA-30 from Kruss GmbH. Prior to testing, the samples were compressed into smooth, thin sheets.

### DFT calculation methods

Based on the catalyst synthesis and experimental characterisations, the CuCo structure was modelled as a CuCo (111) slab with alternating Cu and Co atomic rows and a Cu:Co ratio of 1:1. The spin-polarised density functional theory (DFT) calculations^31^ were performed using the Vienna ab initio simulation package (VASP 5.4.4) based on the projector augmented-wave method with a plane-wave basis set^32,33^. The exchange-correlation potential was treated by using a generalised gradient approximation (GGA) with the Perdew–Burke–Ernzerhof (PBE) parametrisation^34^. The three-layer CuCo (111) unit cell was constructed as a rectangular orthogonal cell obtained through a linear transformation of the rhombic lattice vectors, with the atomic configuration unchanged. A vacuum layer of 15 Å was applied to avoid the interaction between periodic images. The plane-wave energy cutoff was set to 450 eV. The calculations used the van der Waals correction (DFT–D3). The convergence of the Monkhorst–Pack *k*-point sampling was confirmed by comparing different meshes (Fig. S25), and a 2 × 2 × 1 mesh was adopted for structural optimisation^35^. The structures were fully relaxed until the maximum force on each atom was less than 0.05 eV Å^−1^, and the energy convergence criterion was set to 10^−5^ eV^36^.

The Gibbs free energy change (ΔG) for each elementary step was calculated as

1 $$\Delta G=\Delta E+\Delta {E}_{{{\rm{ZPE}}}}-T\Delta S+\Delta {G}_{{{\rm{pH}}}}$$

where ΔE, $\Delta {E}_{{{\rm{ZPE}}}}$, and ΔS represent the changes in total energy, zero-point energy, and entropy, respectively; T is 25 °C. $\Delta {G}_{{{\rm{pH}}}}$ is the pH correction to the Gibbs free energy, and the pH was set to zero throughout this work.

The adsorption energy ($\Delta {E}_{{{\rm{ads}}}}$) was calculated according to

2 $${E}_{{{\rm{ads}}}}={E}_{{{\rm{total}}}}-{E}_{{{\rm{slab}}}}-{E}_{{{\rm{adsorbate}}}}$$

where ${E}_{{{\rm{total}}}}$ is the total energy of the adsorption system, ${E}_{{{\rm{slab}}}}$ is the energy of the clean slab, and ${E}_{{{\rm{adsorbate}}}}$ is the energy of the isolated adsorbate.

### Catalytic evaluation

The catalytic activity evaluation of the catalyst was carried out on a high-pressure fixed-bed continuous flow reactor. A mixture of 0.3 g of catalyst and 1.7 g of quartz sand was evenly packed into the reaction tube. The catalyst was activated by 15% CH_4_/H_2_ under atmospheric pressure at 590 °C for 2 h. The gas products CO, CO_2_, CH_4_, and N_2_ were detected by GC 2060 equipped with a TCD and a TDX-01 packed column. Hydrocarbon and oxygenate compounds were analysed online using an Agilent 7890 gas chromatography equipped with FID, KB–PONA, and KB–Al_2_O_3_/Na_2_SO_4_ capillary columns.

Conversion was calculated using the following equation

3 $${X}_{{{\rm{CO}}}}=\frac{{n}_{{{\rm{CO}}},{{\rm{inlet}}}}-{n}_{{{\rm{CO}}},{{\rm{outlet}}}}}{{n}_{{{\rm{CO}}},{{\rm{inlet}}}}}\times 100\%$$

Selectivity was calculated using the following equation

4 $${S}_{i}=\frac{{M}_{i}\times {N}_{i}}{\Sigma ({M}_{i}\times {N}_{i})}\times 100\%$$

${n}_{{\mathrm{CO}},{\mathrm{outlet}}}$ and ${n}_{{{\rm{CO}}},{{\rm{outlet}}}}$ represent the inlet and outlet molar concentration of CO, respectively.$\,{M}_{i}$ and ${N}_{i}$ represent the moles and carbons of product i, respectively.

## Supplementary information

Supplementary Information

Transparent Peer Review file

## Source data

Source Data

## Data Availability

The Source Data provided with this article contains the numerical data underlying the figures presented in this study. The structural files corresponding to the DFT-calculated reaction pathways are available at Figshare^37^: https://doi.org/10.6084/m9.figshare.33306180. Data are available from the corresponding authors upon request. Source data are provided with this paper.
