# Supplementary material for: Dynamic carbon overlayered CuCo alloy promotes syngas to higher alcohols
Source: Nat Commun. 2026 Sep 30;17:11239. doi: 10.1038/s41467-026-78203-7 (PMC13720033; doi:10.1038/s41467-026-78203-7)
Supplement: Supplementary file 1 [file 41467_2026_78203_MOESM1_ESM.pdf]

Supplementary Information for

Dynamic carbon overlayered CuCo alloy promotes syngas to higher alcohols

Jia Liu[1], Si-Wei Ying[1], Jiachang Zuo[1], Qiuyue Zhang[1], Shan Gao[1], Xuelian Liang[1], Xinping Duan[1], Jing Ren[2], Yufei Zhao[2], Mingshu Chen[1], Linmin Ye[1], Su-Yuan Xie[1], Jian-Wei Zheng[1,*] & Youzhu Yuan[1,*]

[1]*State Key Laboratory of Physical Chemistry of Solid Surfaces, College of Chemistry and Chemical Engineering, Xiamen University, Xiamen 361005, China*

[2]*State Key Laboratory of Chemical Resource Engineering, Beijing University of Chemical Technology, Beijing 100029, China*

[*]Corresponding authors: jwzheng@xmu.edu.cn; yzyuan@xmu.edu.cn

Supplementary Figures

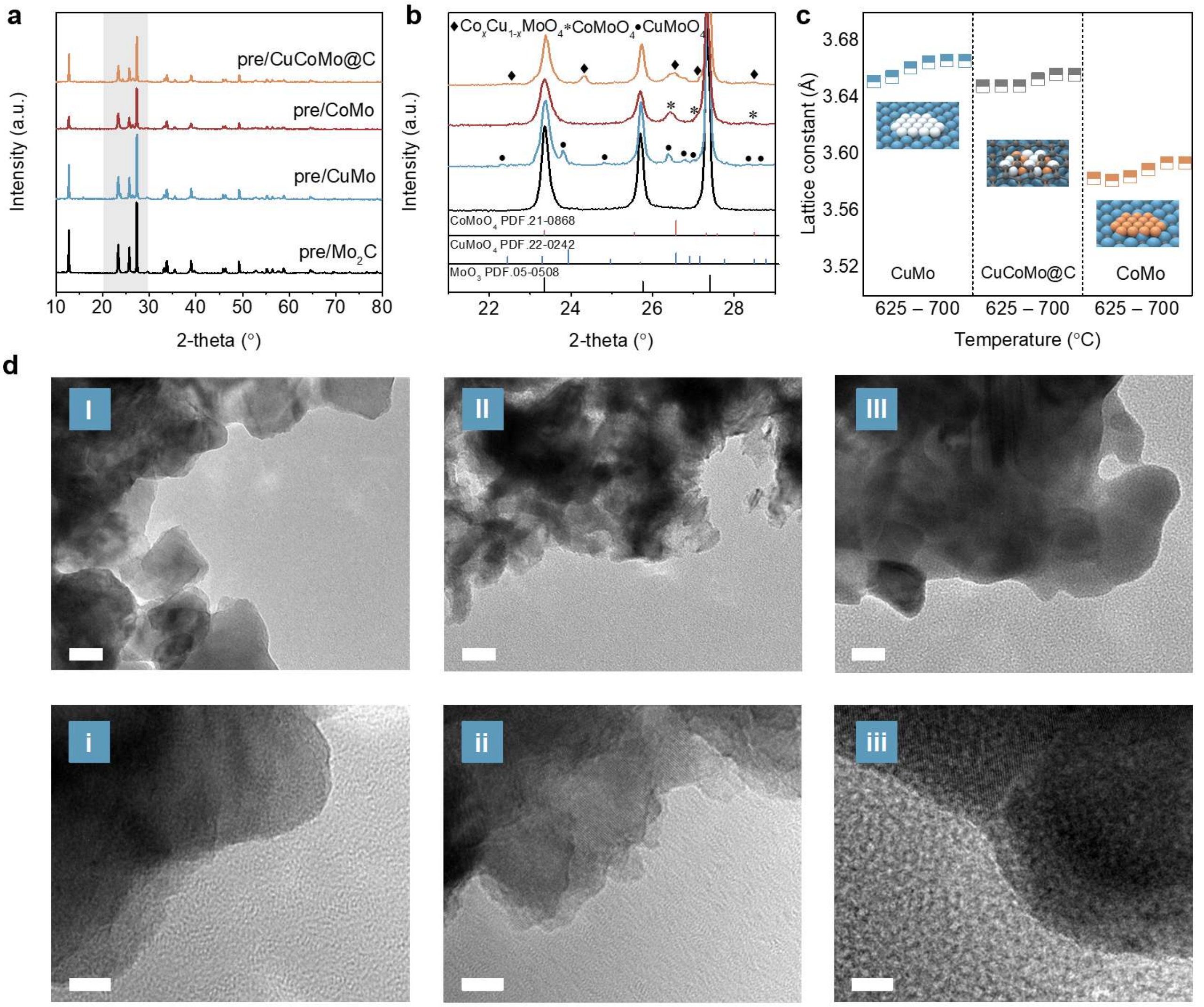

Fig. S1 | Structural characterisation of the catalysts. (a) XRD patterns of the Mo_2C, CuMo, CoMo, and CuCoMo@C precursors. (b) Enlarged view of Fig. S1a in the range of 21.0°–29.0°. (c) Cu lattice constants of CuMo and CuCoMo@C, and the Co lattice constants of CoMo at different temperatures. (d) HRTEM images of the CuCoMo-based catalysts. I, Mo_2C. Scale bar, 20 nm. i, Mo_2C. Scale bar, 5 nm. II, CuMo. Scale bar, 20 nm. ii, CuMo. Scale bar, 5 nm. III, CoMo. Scale bar, 20 nm. iii, CoMo. Scale bar, 5 nm.

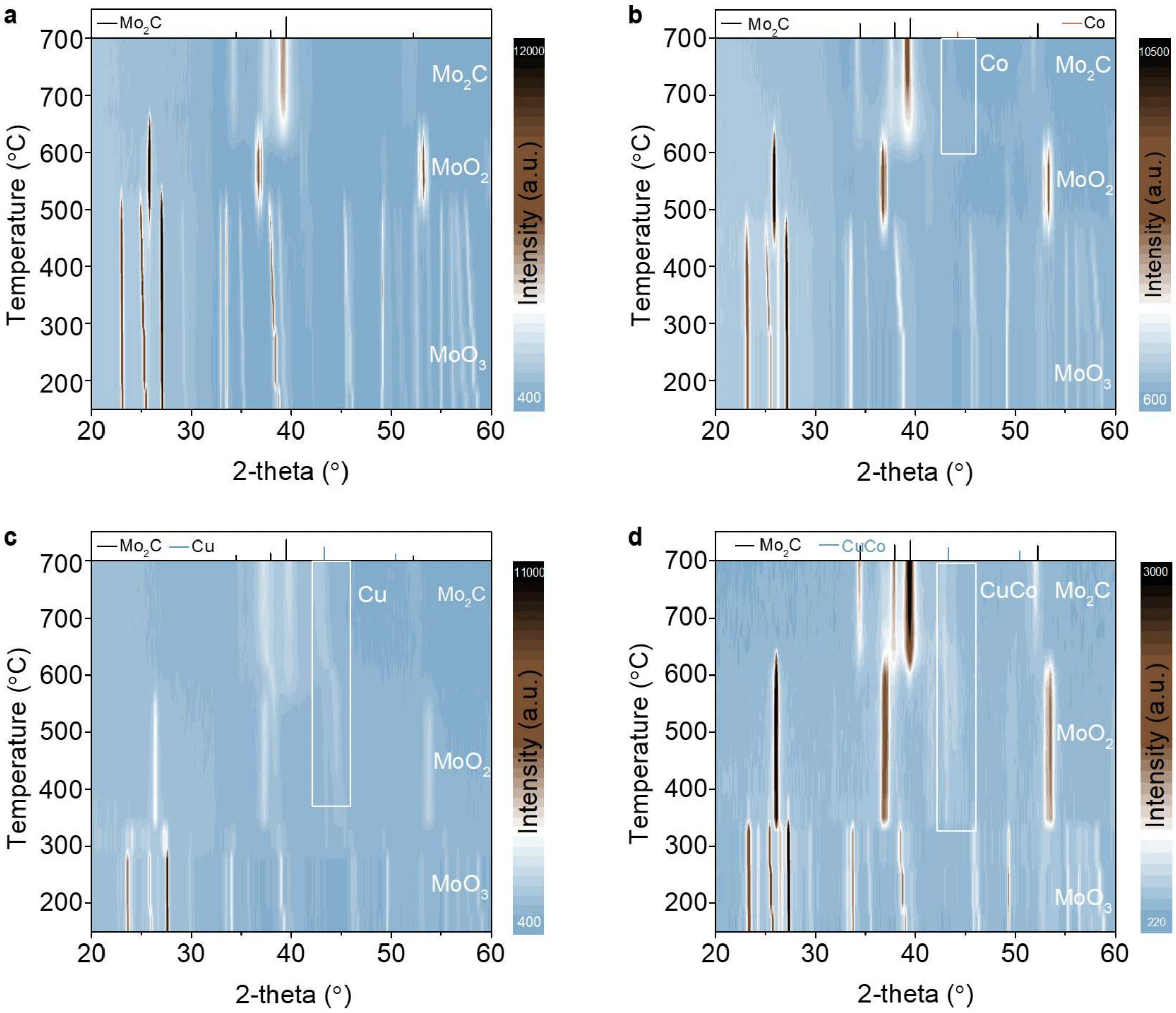

Fig. S2 | Structural evolution during the carburisation process. (a–d) In situ XRD patterns of the Mo_2C, CoMo, CuMo and CuCoMo@C catalysts. Carburisation conditions: 15% CH_4/H_2 at 50 mL min^{-1}, heated from 30 to 700 °C at a ramping rate of 1 °C min^{-1}.

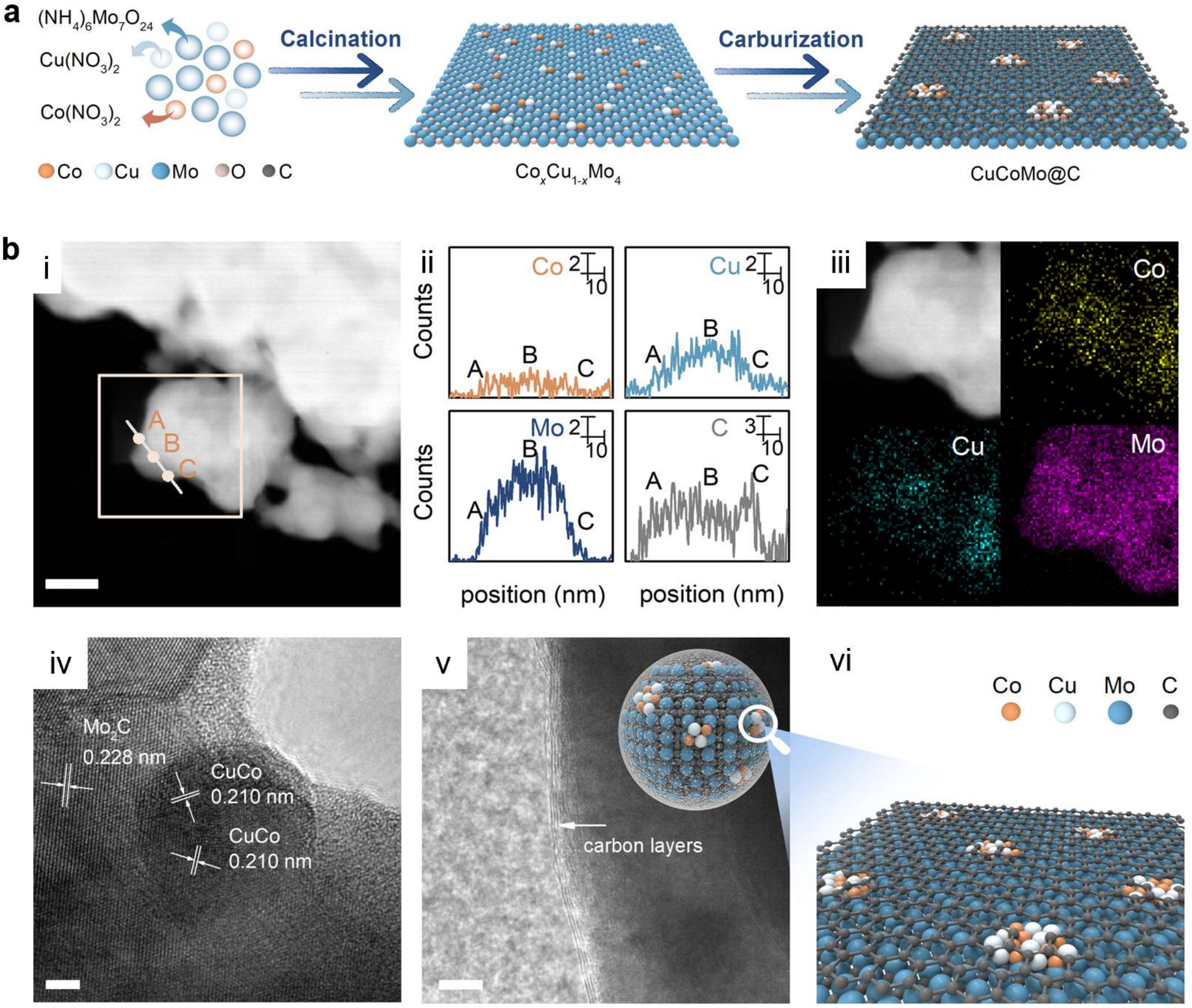

Fig. S3 | Catalyst synthesis and microstructure. (a) Schematic diagram of the CuCoMo@C catalyst preparation process. (b) TEM images. i, HAADF–STEM image. Scale bar, 100 nm. ii, STEM–EDS line-scan profiles of Co, Cu, Mo, and C (along the selected line in Fig. S3b-i, with spots A, B, and C indicated). iii, STEM–EDS elemental maps of the selected region for Cu, Co, and Mo. iv, HRTEM image. Scale bar, 2 nm. v, HRTEM image. Scale bar, 5 nm. vi, Schematic diagram.

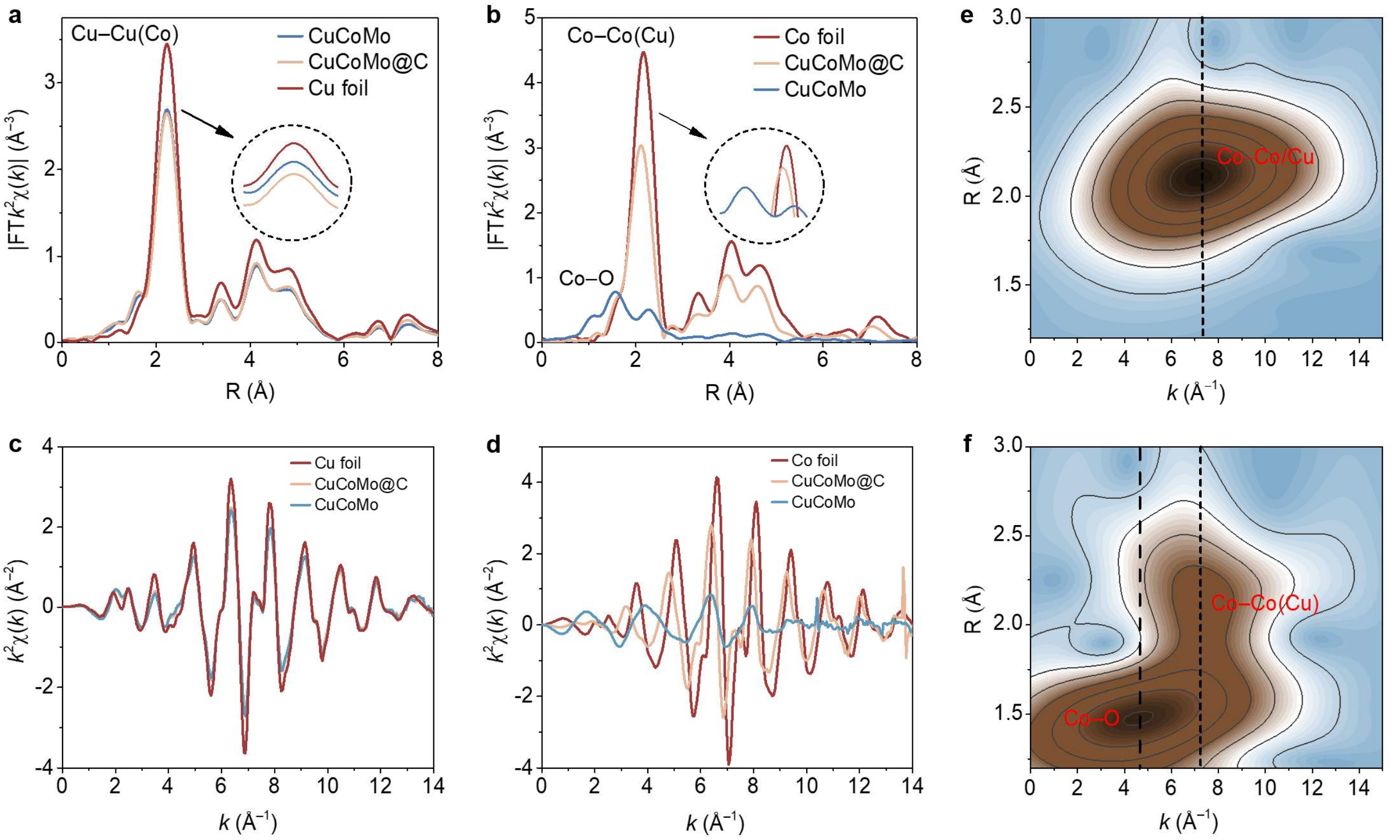

Fig. S4 | XAS profiles. (a–b) EXAFS R-space profile of Cu K-edge and Co K-edge. (c–d) EXAFS *k*-space profiles of Cu K-edge and Co K-edge. (e–f) Wavelet transform (WT) contour plots of Co K-edge for the CuCoMo@C and CuCoMo catalysts.

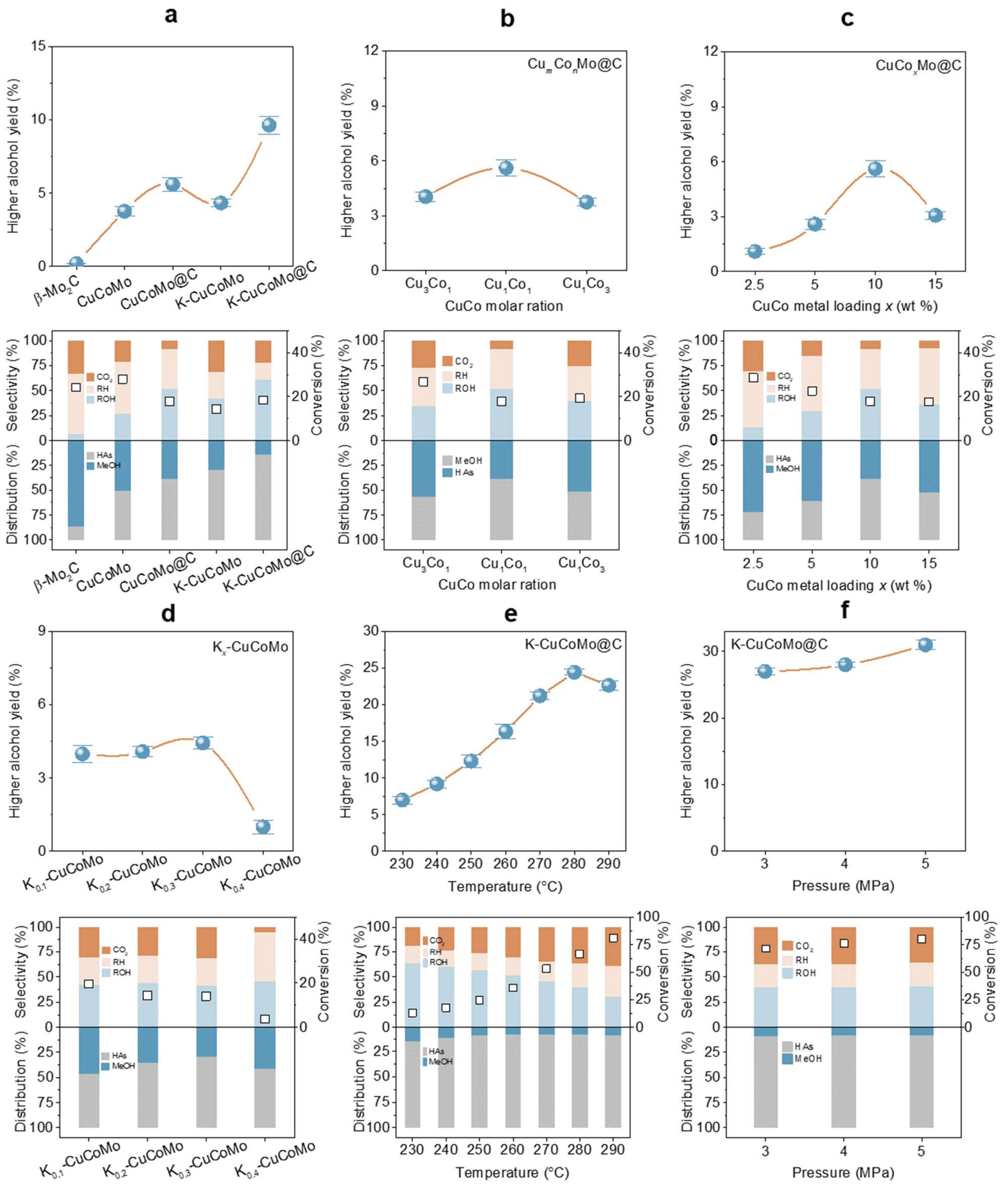

Fig. S5 | Catalytic performance under different reaction conditions. (a) Catalytic performance of different catalysts. The metal loading of Cu and Co is 10 wt%. The molar ratio of Cu and Co is 1:1. Reaction conditions: GHSV = 4 L g_{cat}^{-1} h^{-1}, T = 240 °C, P = 3.0 MPa, and H_2/CO = 2. (b) Catalytic performance of the Cu$_m$Co$_n$Mo@C (m and n: Cu and Co molar ratio) catalysts with different Cu and Co molar ratios. The metal loading of Cu and Co is 10 wt%. Reaction conditions: GHSV = 4 L g_{cat}^{-1}

h^{-1}, T = 240 °C, P = 3.0 MPa, and H_2/CO = 2. (c) Catalytic performance of the $CuCo_x$Mo@C (x: Cu and Co metal loading, wt%) catalysts with different Cu and Co metal loadings. The molar ratio of Cu and Co is 1:1. Reaction conditions: GHSV = 4 L g_{cat}^{-1} h^{-1}, T = 240 °C, P = 3.0 MPa, and H_2/CO = 2. (d) Catalytic performance of the K_x-CuCoMo (x: K loading, wt%) catalysts with different K loadings. The metal loading of Cu and Co is 10 wt%. The molar ratio of Cu and Co is 1:1. Reaction conditions: GHSV = 4 L g_{cat}^{-1} h^{-1}, T = 240 °C, P = 3.0 MPa, and H_2/CO = 2. (e) Catalytic performance of the K-CuCoMo@C catalysts at different reaction temperatures. The metal loading of Cu and Co is 10 wt%. The molar ratio of Cu and Co is 1:1. The loading of K is 0.3 wt%. Reaction conditions: GHSV = 4 L g_{cat}^{-1} h^{-1}, P = 3.0 MPa, and H_2/CO = 2. (f) Catalytic performance of the K-CuCoMo@C catalysts at different reaction pressures. The metal loading of Cu and Co is 10 wt%. The molar ratio of Cu and Co is 1:1. The loading of K is 0.3 wt%. Reaction conditions: GHSV = 4 L g_{cat}^{-1} h^{-1}, T = 280 °C and H_2/CO = 2. Error bars represent SD. Yield: yield on a carbon-number basis.

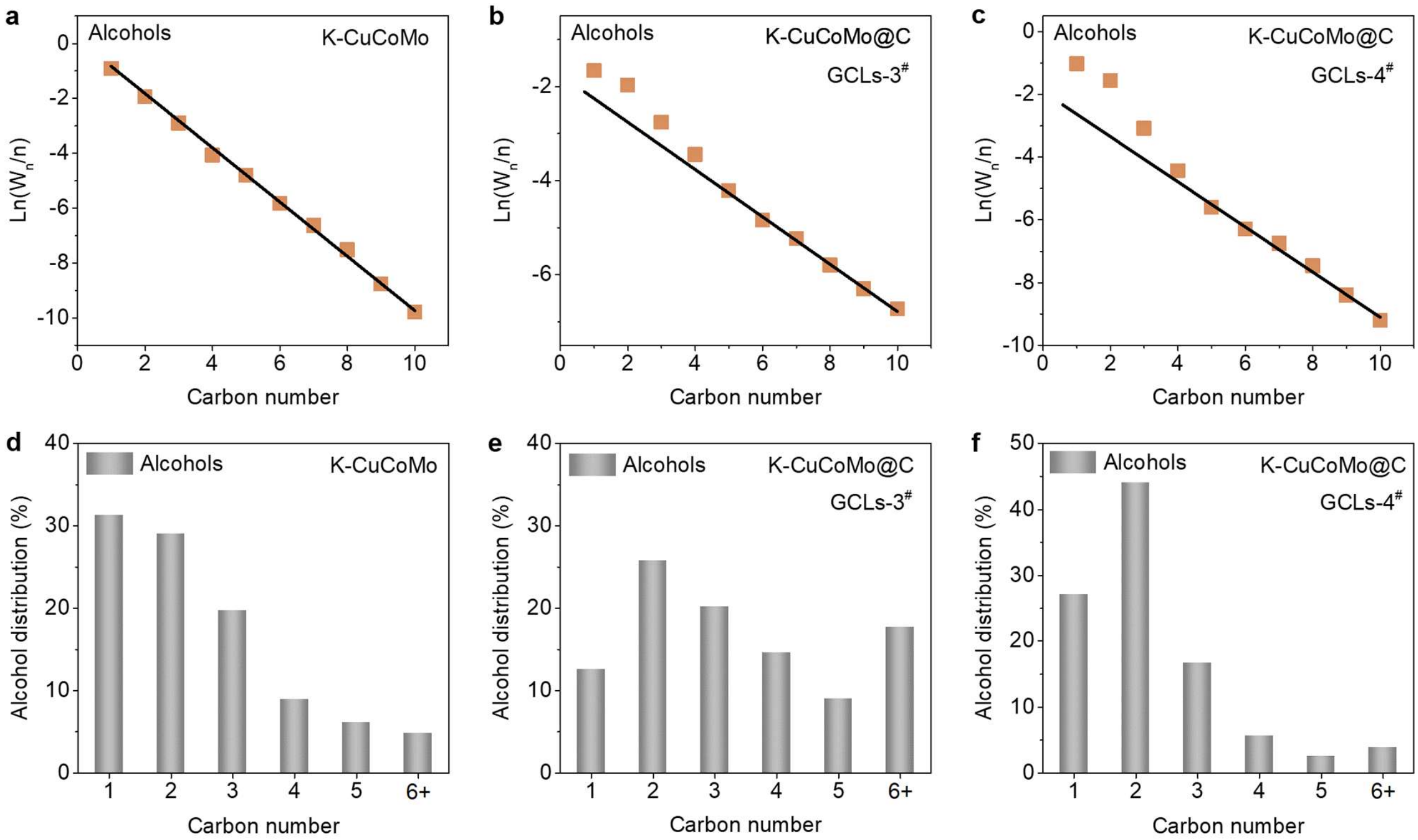

Fig. S6 | Kinetic analysis. (a–c) Alcohol chain-growth probability ($\alpha_{Alcohols}$) of the K-CuCoMo, K-CuCoMo@C (GCLs-3$^{\#}$), and K-CuCoMo@C (GCLs-4$^{\#}$) catalysts. (d–f) Alcohol distribution of the K-CuCoMo, K-CuCoMo@C (GCLs-3$^{\#}$), and K-CuCoMo@C (GCLs-4$^{\#}$) catalysts. Reaction conditions: GHSV = 8 L g_{cat}^{-1} h^{-1}, T = 240 °C, P = 3.0 MPa, and H_2/CO = 2.

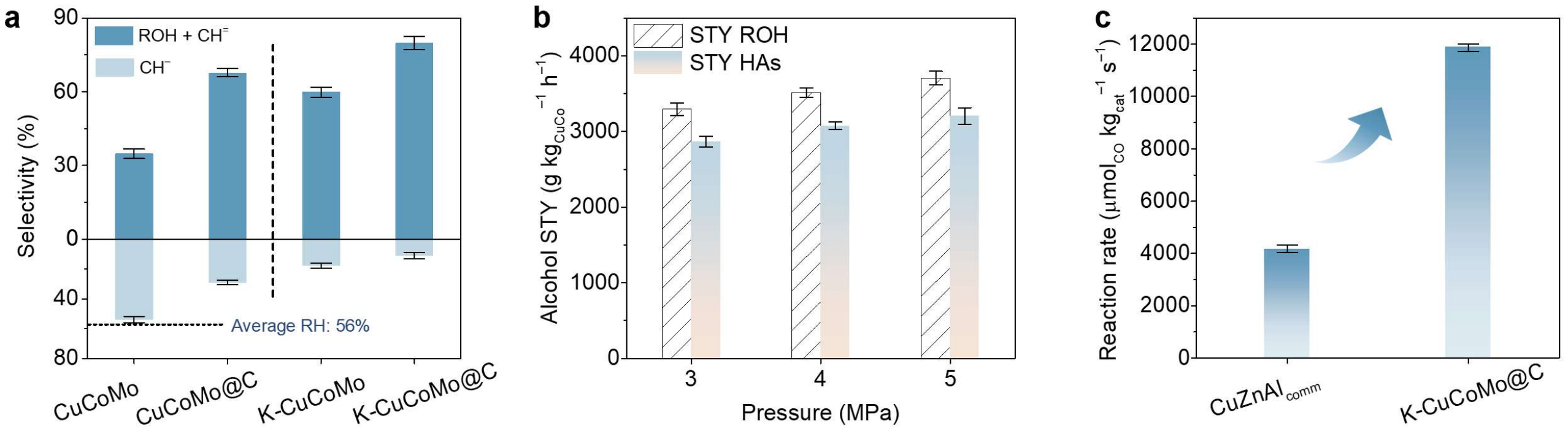

Fig. S7 | Catalytic performance. (a) Hydrogenation product selectivity for the (K-)CuCoMo and (K-)CuCoMo@C catalysts. Reaction conditions: T = 240 °C, P = 3.0 MPa, and GHSV = 8 L g_{cat}^{-1} h^{-1}. (b) Alcohol STY for the K-CuCoMo@C catalyst at different pressures. Reaction conditions: T = 280 °C, and GHSV = 4 L g_{cat}^{-1} h^{-1}. (c) Reaction rates of the commercial CuZnAl and K-CuCoMo@C catalysts. Error bars represent SD. Reaction conditions: T = 280 °C, P = 5.0 MPa, and GHSV = 4 L g_{cat}^{-1} h^{-1}.

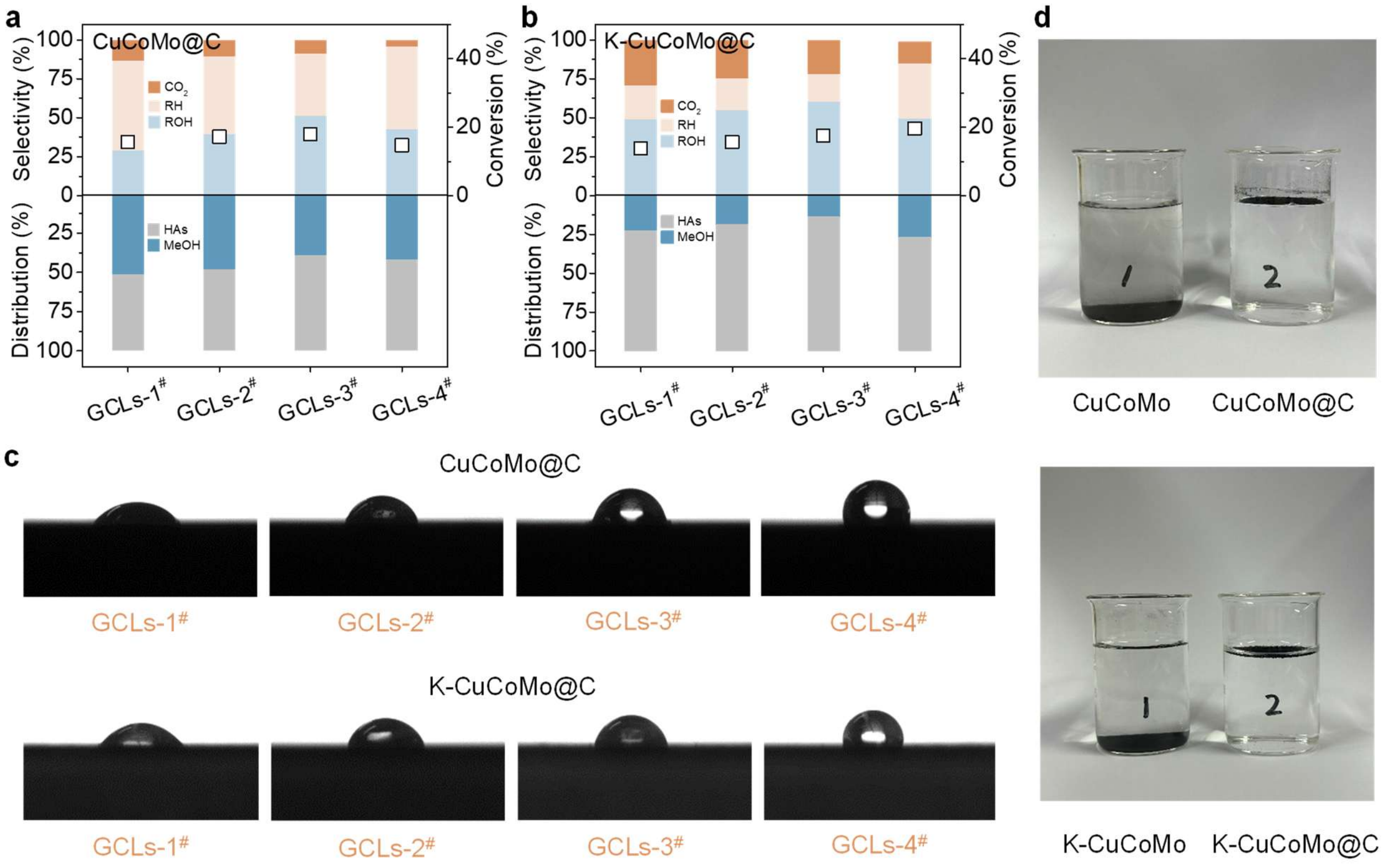

Fig. S8 | Hydrophobic properties of the catalysts. (a) Selectivity, conversion, and alcohol distribution of the CuCoMo@C catalysts with different carbon amounts. (b) Selectivity, conversion, and alcohol distribution of the K-CuCoMo@C catalysts with different carbon amounts. The metal loading of Cu and Co is 10 wt%, the molar ratio of Cu and Co is 1:1, and the loading of K is 0.3 wt%. Reaction conditions: GHSV = 4 L g_{cat}^{-1} h^{-1}, T = 240 °C, P = 3.0 MPa, and H_2/CO = 2. (c) Contact angle of the CuCoMo@C and K-CuCoMo@C catalysts with different carbon amounts. GCLs-1# to GCLs-4# denote catalyst samples No. 1–4 with different carbon amounts. (d) Hydrophobic properties of the catalysts in water. The CuCoMo@C and K-CuCoMo@C catalysts shown are GCLs-4#.

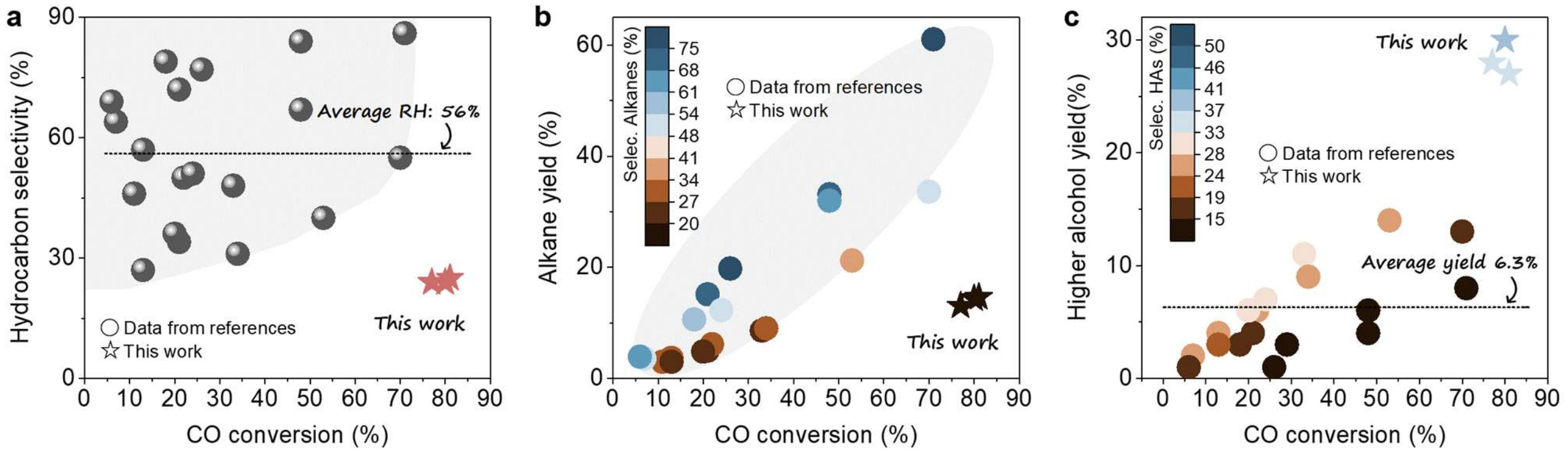

Fig. S9 | Catalytic performance comparisons. (a) Comparison of the hydrocarbon selectivity with reported catalysts. (b) Comparison of the yield and selectivity of alkanes with reported catalysts. (c) Comparison of the yield and selectivity of HAs with reported catalysts. Data from references (○): refs.[38–53]. This work (☆): K-CuCoMo@C.

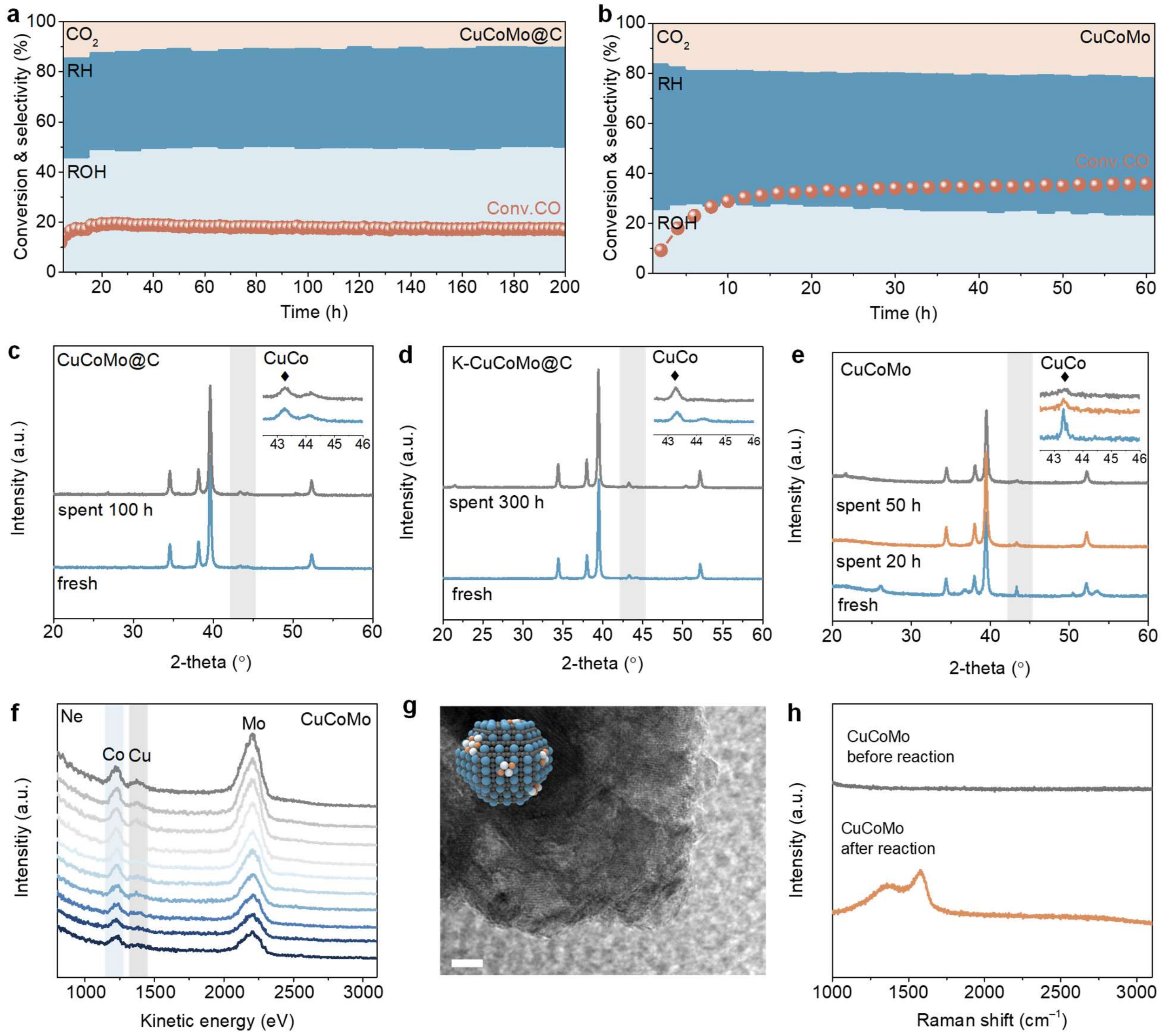

Fig. S10 | Catalyst stability evaluation. (a) 200 h catalytic performance of the CuCoMo@C catalyst. (b) 60 h catalytic performance of the CuCoMo catalyst. Reaction conditions: GHSV = 4 L g_{cat}^{-1} h^{-1}, T = 240 °C, P = 3.0 MPa, and H_2/CO = 2. (c–e) XRD patterns of the CuCoMo@C, K-CuCoMo@C, and CuCoMo catalysts before and after reaction. (f) Ne spectra from layer-by-layer etching LEIS of the fresh CuCoMo catalyst. (g) HRTEM images of the fresh CuCoMo catalyst. Scale bar, 5 nm. (h) Raman spectra of the CuCoMo catalyst before and after reaction.

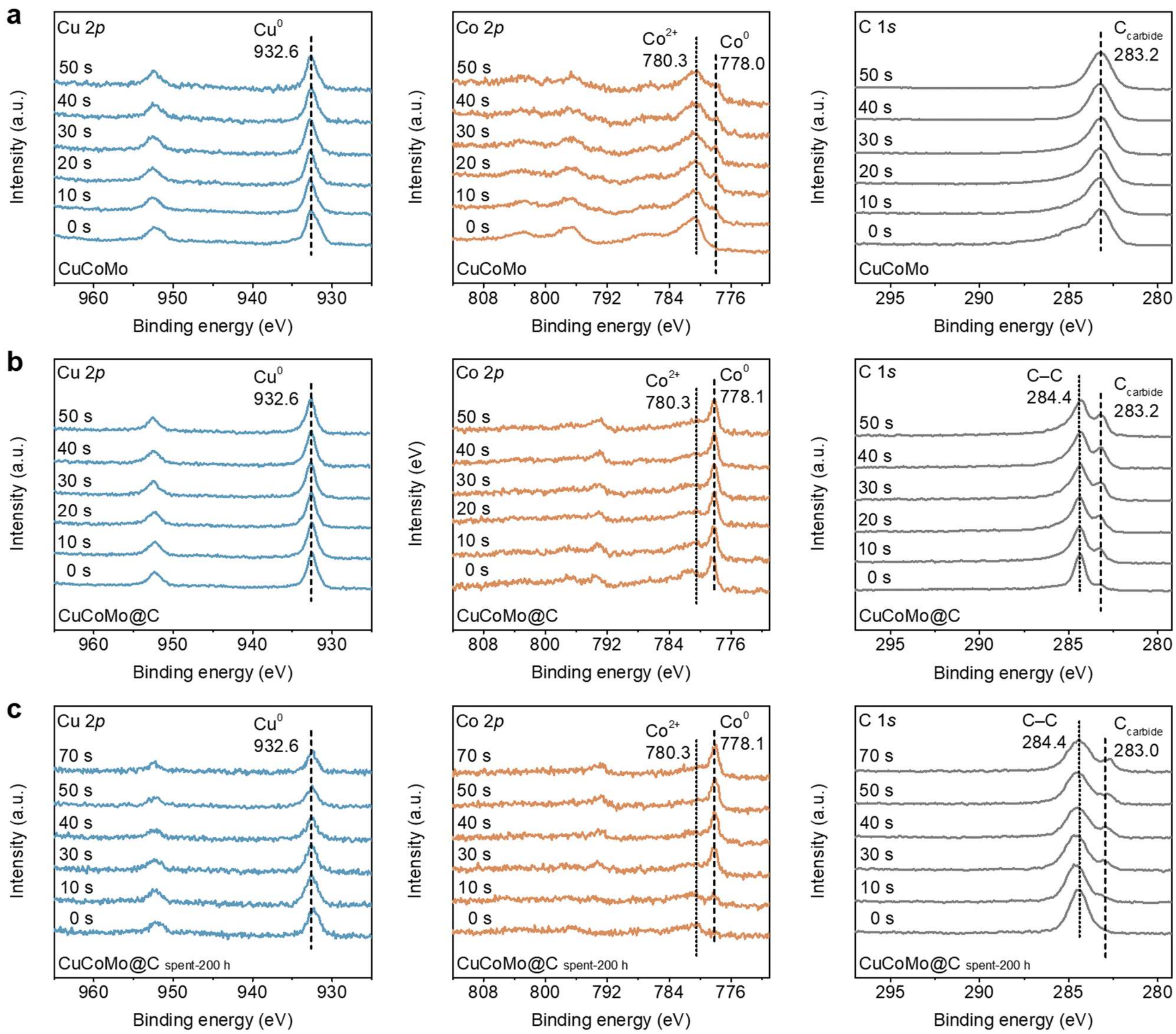

Fig. S11 | Ar-ion etching XPS results of the catalysts. (a) Binding energies of C, Co, and Cu in the CuCoMo catalyst before reaction. (b) Binding energies of C, Co, and Cu in the CuCoMo@C catalyst before reaction. (c) Binding energies of C, Co, and Cu in the CuCoMo@C catalyst after a 200 h reaction.

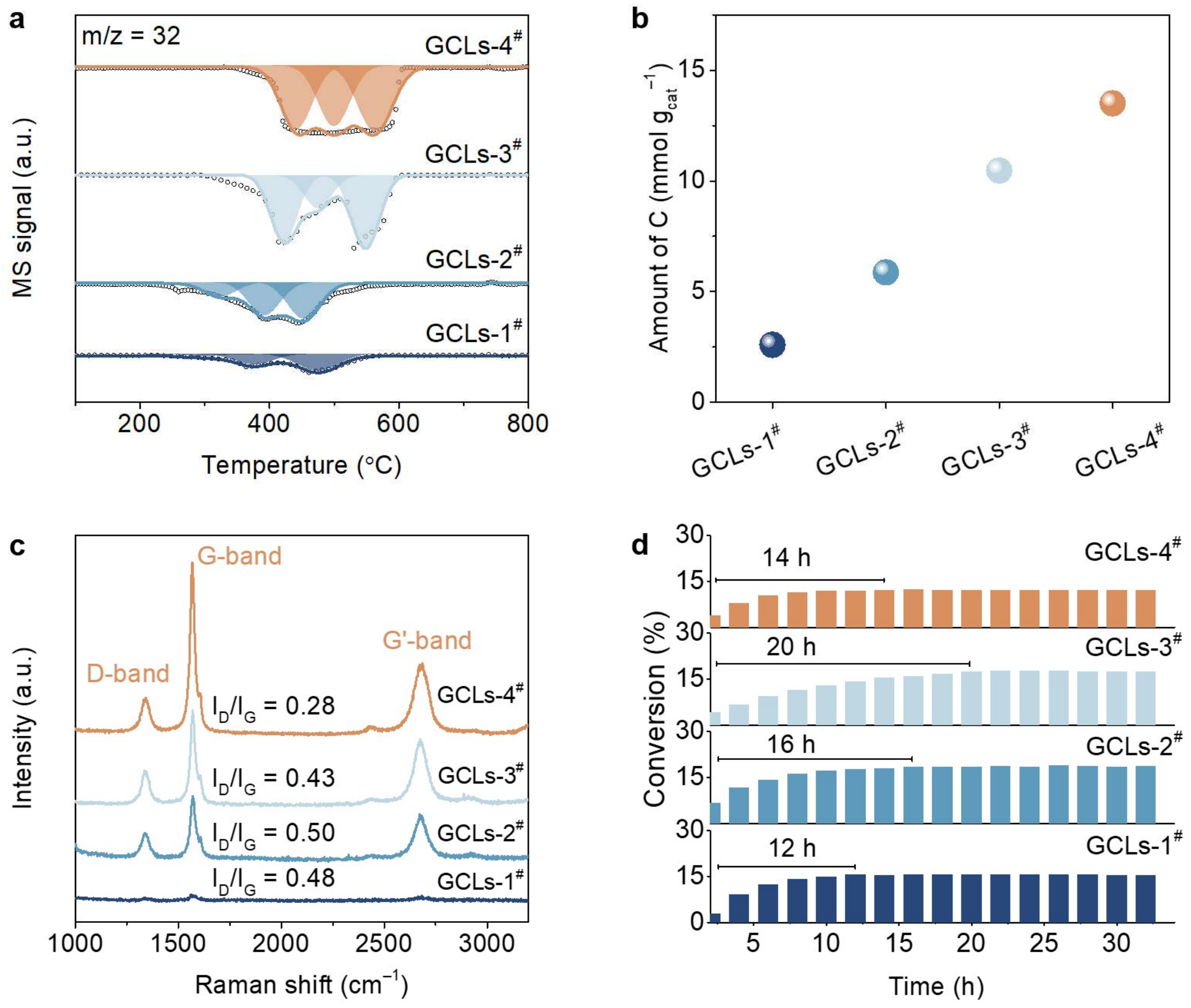

Fig. S12 | Quantitative analyses of carbon overlayers. (a) O_2–TPO profiles (monitored at m/z = 32). (b) Carbon amount of the CuCoMo@C catalysts. (c) Raman spectra of the CuCoMo@C catalysts with different carbon amounts. (d) CO conversion of the CuCoMo@C catalysts with different carbon amounts. Reaction conditions: GHSV = 4 L g_{cat}^{-1} h^{-1}, T = 240 °C, P = 3.0 MPa, and H_2/CO = 2.

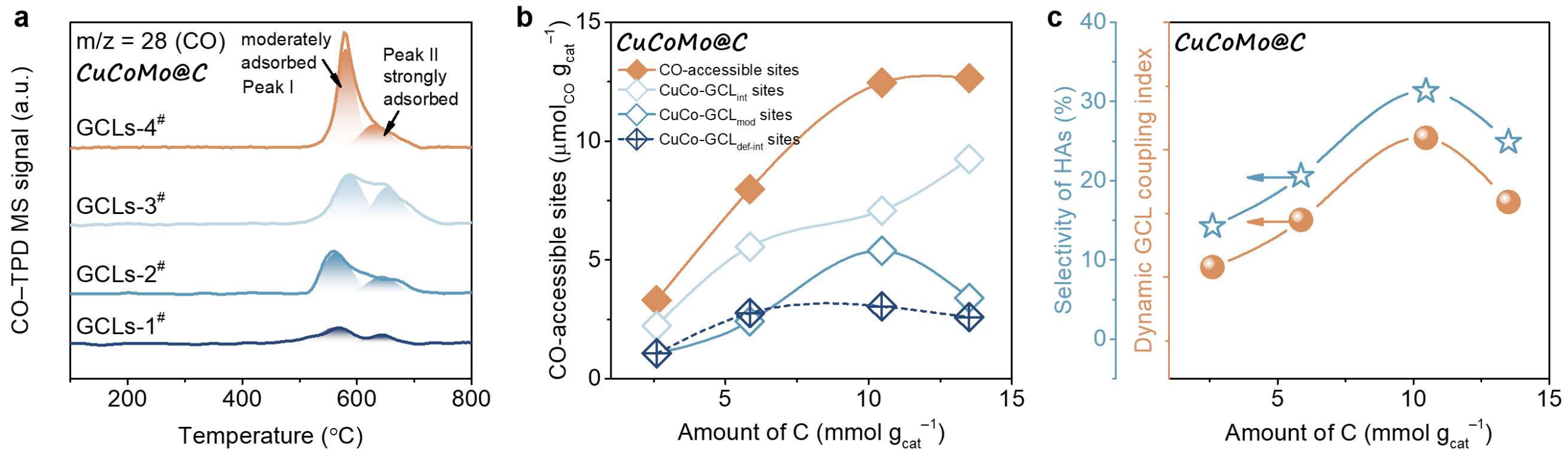

Fig. S13 | Quantification of carbon-overlayer-regulated interfacial adsorption sites. (a) CO–TPD (m/z = 28) profiles of the CuCoMo@C catalysts with different carbon amounts. Two characteristic desorption features are identified: Peak I at lower desorption temperatures is assigned to moderately adsorbed CO on CuCo–GCL interfacial sites and Peak II at higher desorption temperatures is assigned to strongly adsorbed CO on GCL-modified CuCo sites. (b) Quantitative evolution of CO-accessible sites as a function of carbon amount. The total CO-accessible sites were determined by CO pulse adsorption. Based on TPD peak fitting and complementary Raman analyses, the accessible sites are further categorised as follows: CuCo–GCL_{int} sites (CuCo–GCL interfacial sites), CuCo–$GCL_{def\text{-}int}$ sites (defective interfacial carbon sites at the CuCo–GCL interface that participate in C–C coupling reactions; CuCo–$GCL_{def\text{-}int}$ = CuCo–GCL_{int} × I_D/I_G), CuCo–GCL_{mod} sites (GCL-modified CuCo sites that remain accessible to CO/H_2 and generate CO-derived intermediates). (c) Correlation between the HA selectivity and the Dynamic GCL coupling index (DGCI) as a function of carbon amount. DGCI = CuCo–$GCL_{def\text{-}int}$ × CuCo–GCL_{mod}. Reaction conditions: GHSV = 4 L g_{cat}^{-1} h^{-1}, T = 240 °C, P = 3.0 MPa, and H_2/CO = 2.

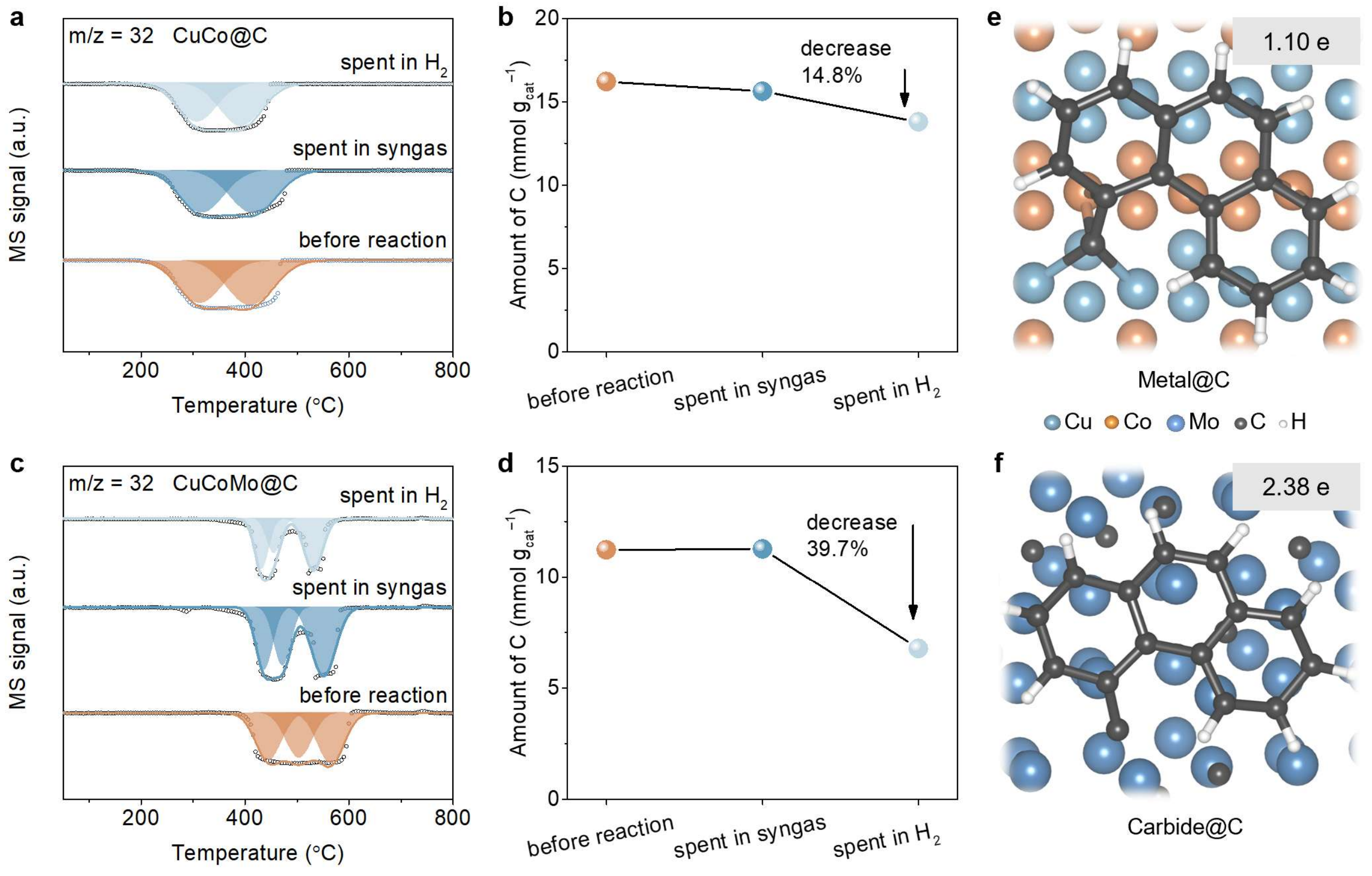

Fig. S14 | Quantitative analysis of carbon overlayer consumption and replenishment. (a) O_2–TPO profiles of the CuCo@C catalyst before reaction, spent in syngas, and spent in H_2 (m/z = 32). (b) Carbon amount of the CuCo@C catalyst at different reaction stages. (c) O_2–TPO profiles of the CuCoMo@C catalyst before reaction, spent in syngas, and spent in H_2 (m/z = 32). (d) Carbon amount of the CuCoMo@C catalyst at different reaction stages. (e) Electron transfer from CuCo to the carbon overlayer. (f) Electron transfer from Mo_2C to the carbon overlayer. Reaction conditions: GHSV = 4 L g_{cat}^{-1} h^{-1}, T = 240 °C, P = 3.0 MPa, under syngas (H_2/CO = 2) or pure H_2.

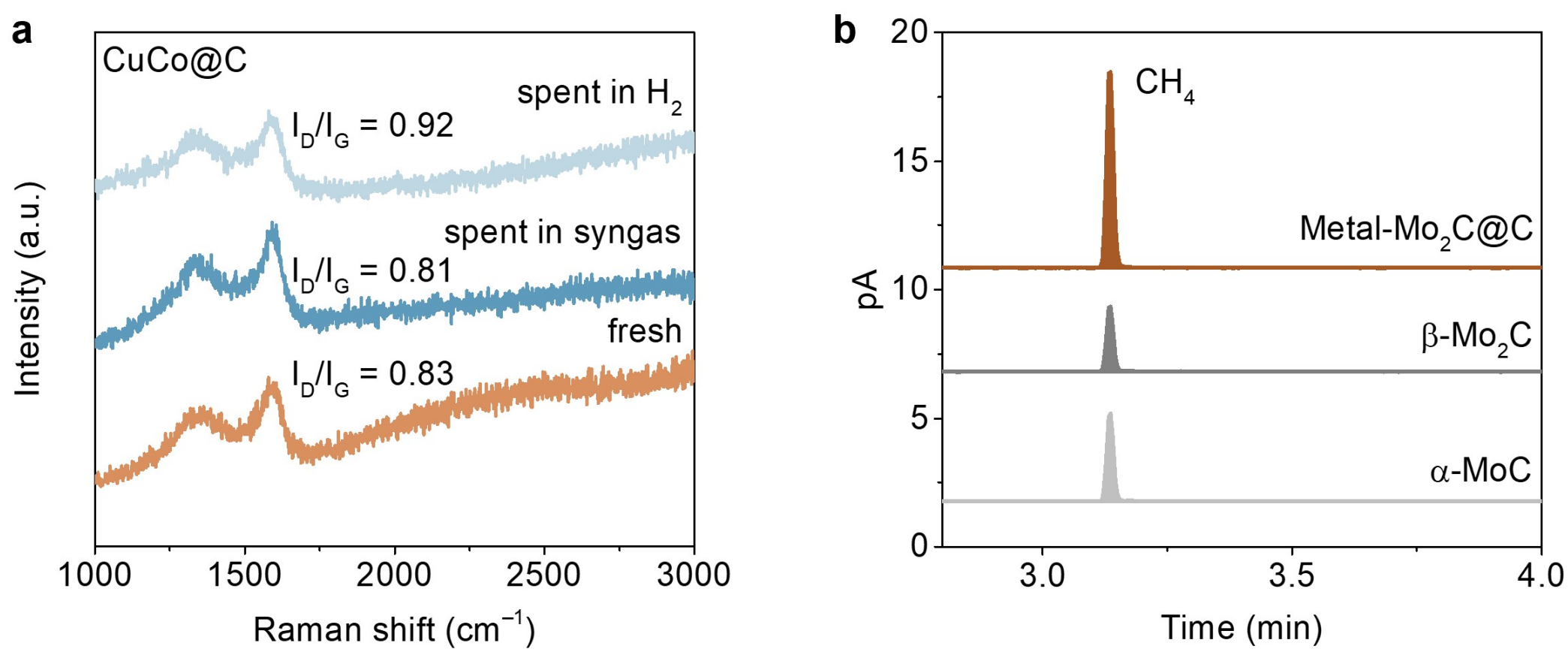

Fig. S15 | Mobility of carbon overlayers. (a) Raman spectra of the CuCo@C catalyst before reaction, spent in syngas, and spent in H_2. (b) Products detected by GC during H_2 reaction. Reaction conditions: T = 240 °C, P = 3.0 MPa, GHSV = 4 L g_{cat}^{-1} h^{-1}, under syngas (H_2/CO = 2) or pure H_2.

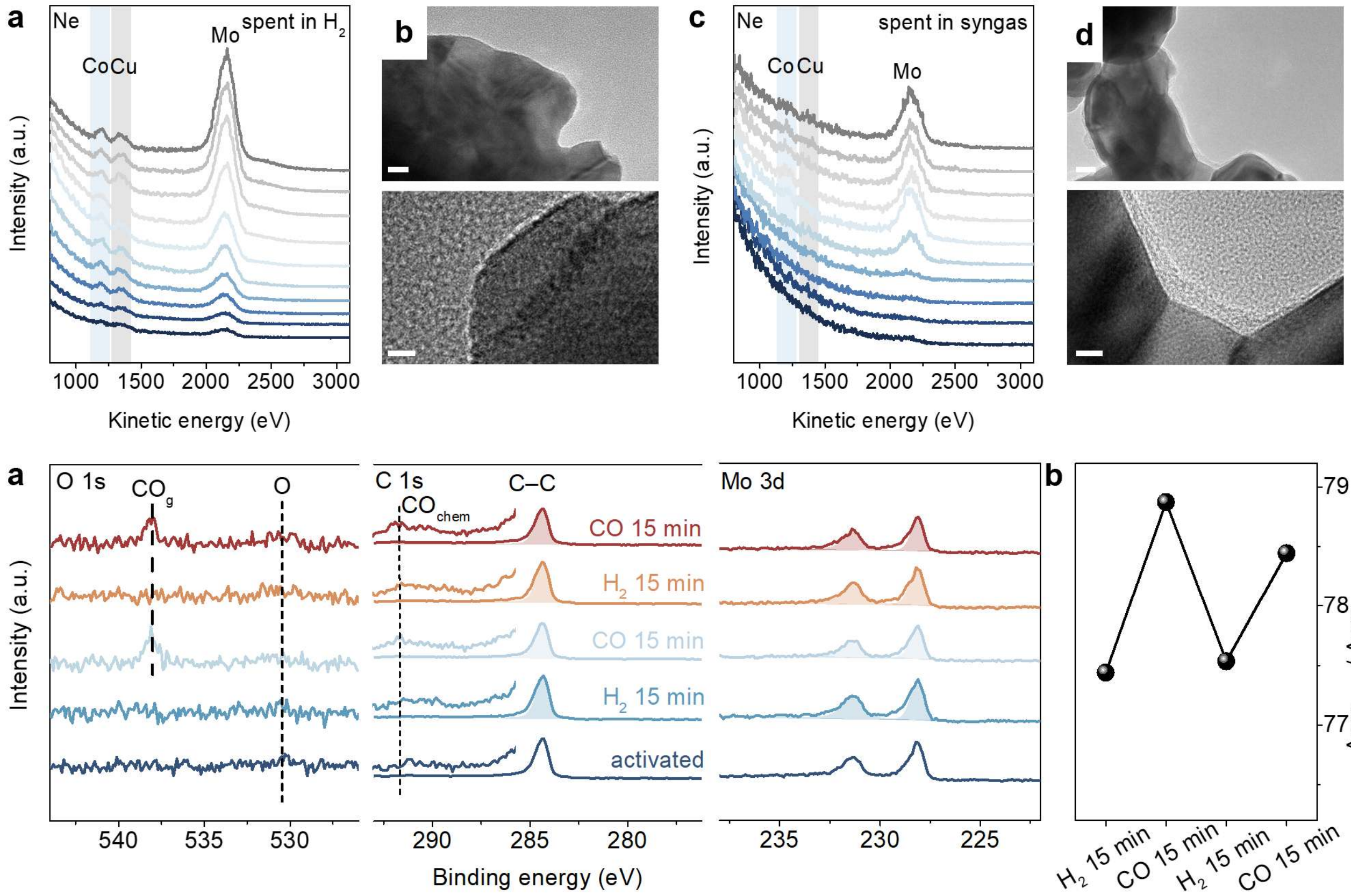

Fig. S16 | Mobility of dynamic carbon overlayers. (a) Ne spectra from layer-by-layer etching LEIS of the CuCoMo@C catalyst spent in H_2. (b) HRTEM images of the CuCoMo@C catalyst spent in H_2. Scale bars, 20 nm and 5 nm. (c) Ne spectra from layer-by-layer etching LEIS of the CuCoMo@C catalyst spent in syngas. (d) HRTEM images of the CuCoMo@C catalyst spent in syngas. Scale bars, 20 nm and 5 nm. (e) NAP–XPS profiles of the CuCoMo@C catalyst after activation, under H_2 for 15 min, under CO for 15 min, under H_2 for 15 min, and under CO for 15 min at 0.4 mbar. (f) Area proportion of the C–C component.

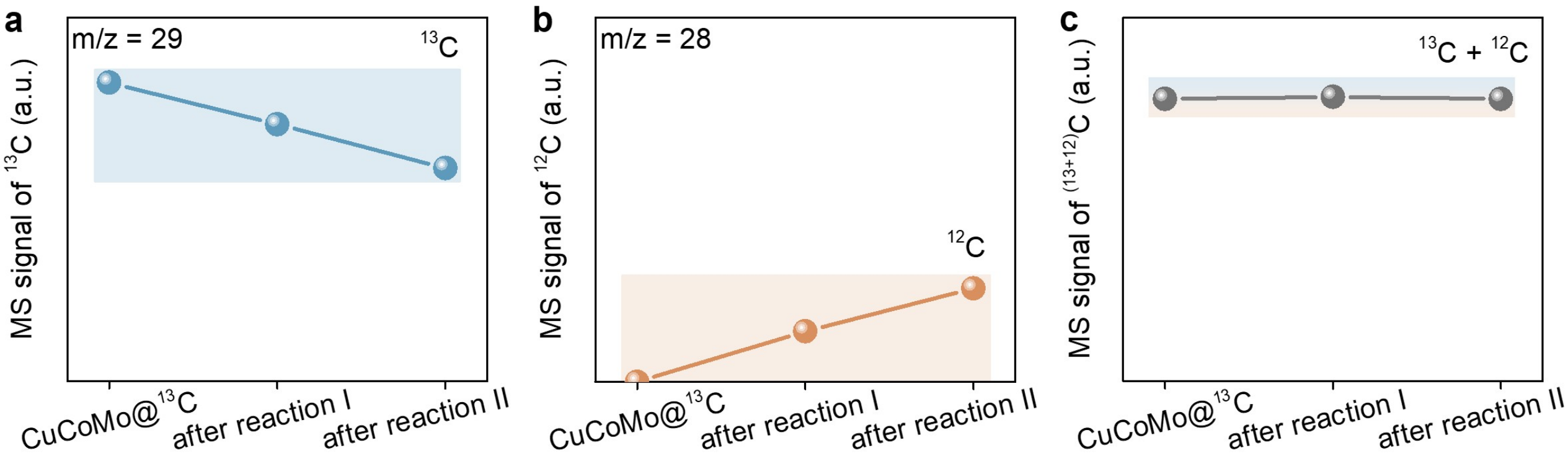

Fig. S17 | Consumption and replenishment of ^{13}C in CuCoMo@^{13}C. (a) MS signal of ^{13}CO. (b) MS signal of ^{12}CO. (c) Combined MS signal of ^{13}CO and ^{12}CO. Reaction conditions: GHSV = 8 L g_{cat}^{-1} h^{-1}, T = 240 °C, P = 3.0 MPa, and H_2/CO = 2.

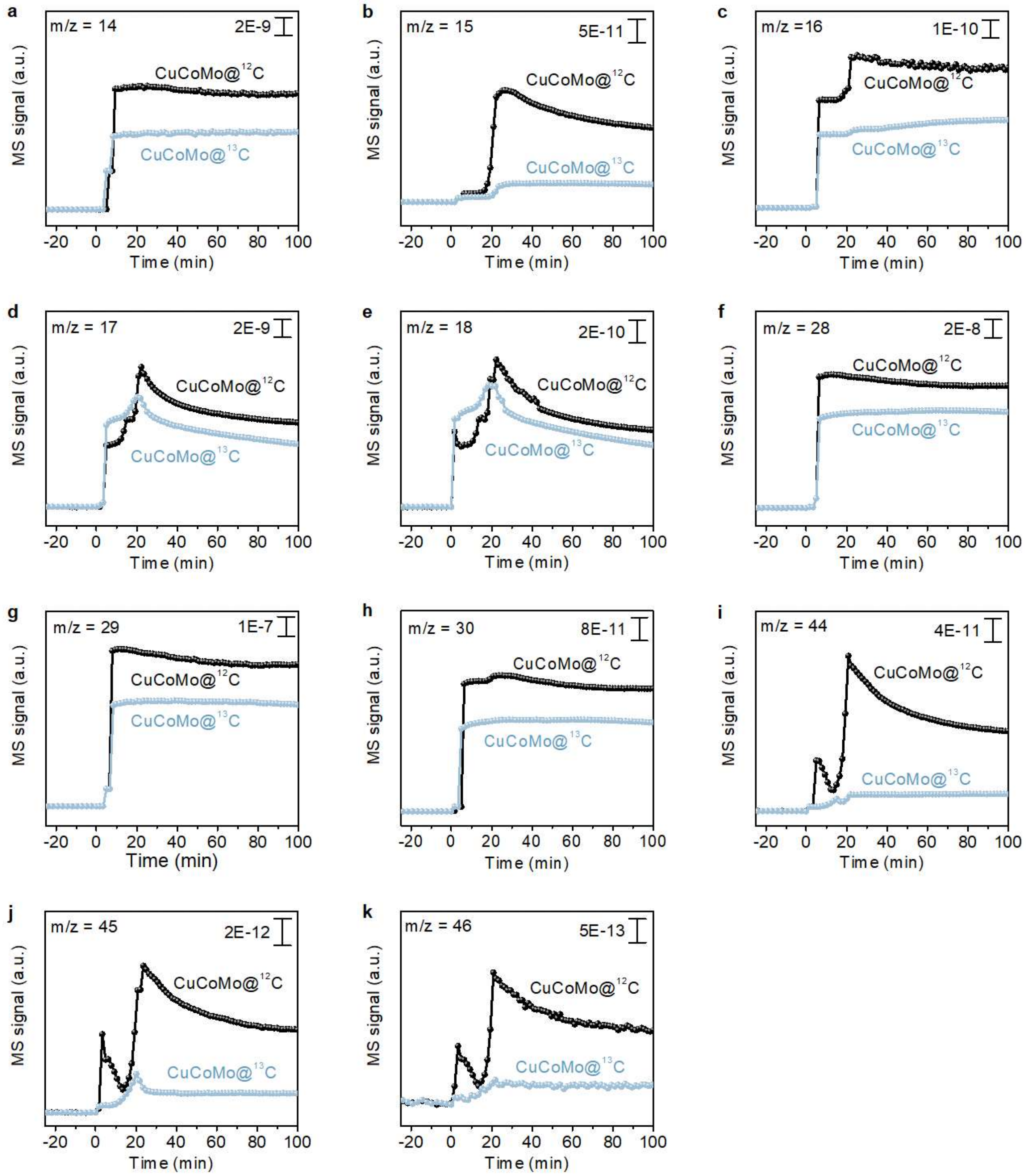

Fig. S18 | TPSR profiles of the CuCoMo@^{12}C and CuCoMo@^{13}C catalysts. (a–k) MS signals of m/z = 14, 15, 16, 17, 18, 28, 29, 30, 44, 45, and 46.

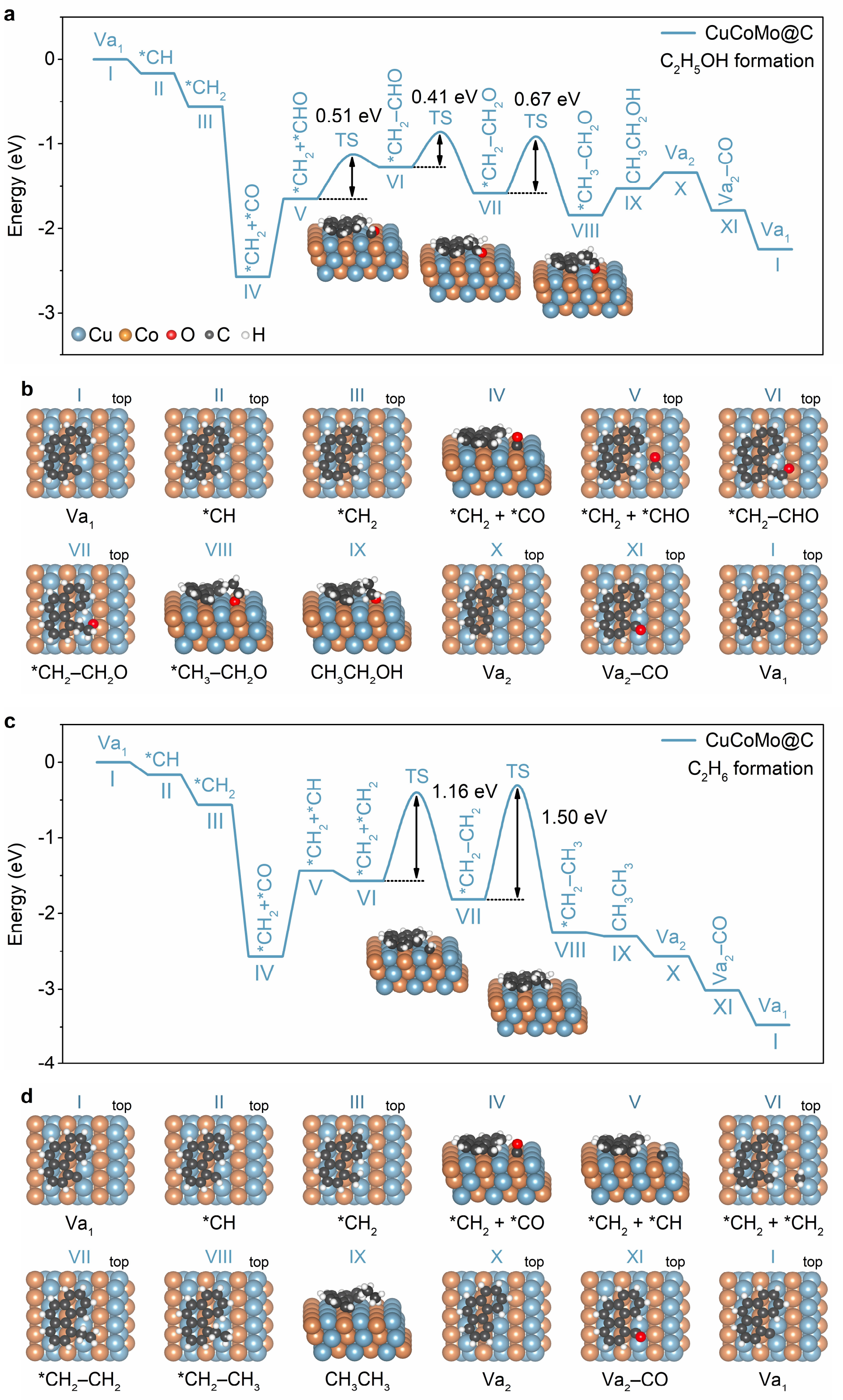

Fig. S19 | Reaction pathways on the CuCoMo@C catalyst. (a) Reaction pathway for C_2H_5OH formation. (b) Views of the adsorbed species for C_2H_5OH formation. (c) Reaction pathway for C_2H_6 formation. (d) Views of the adsorbed species for C_2H_6 formation. Va, carbon vacancy.

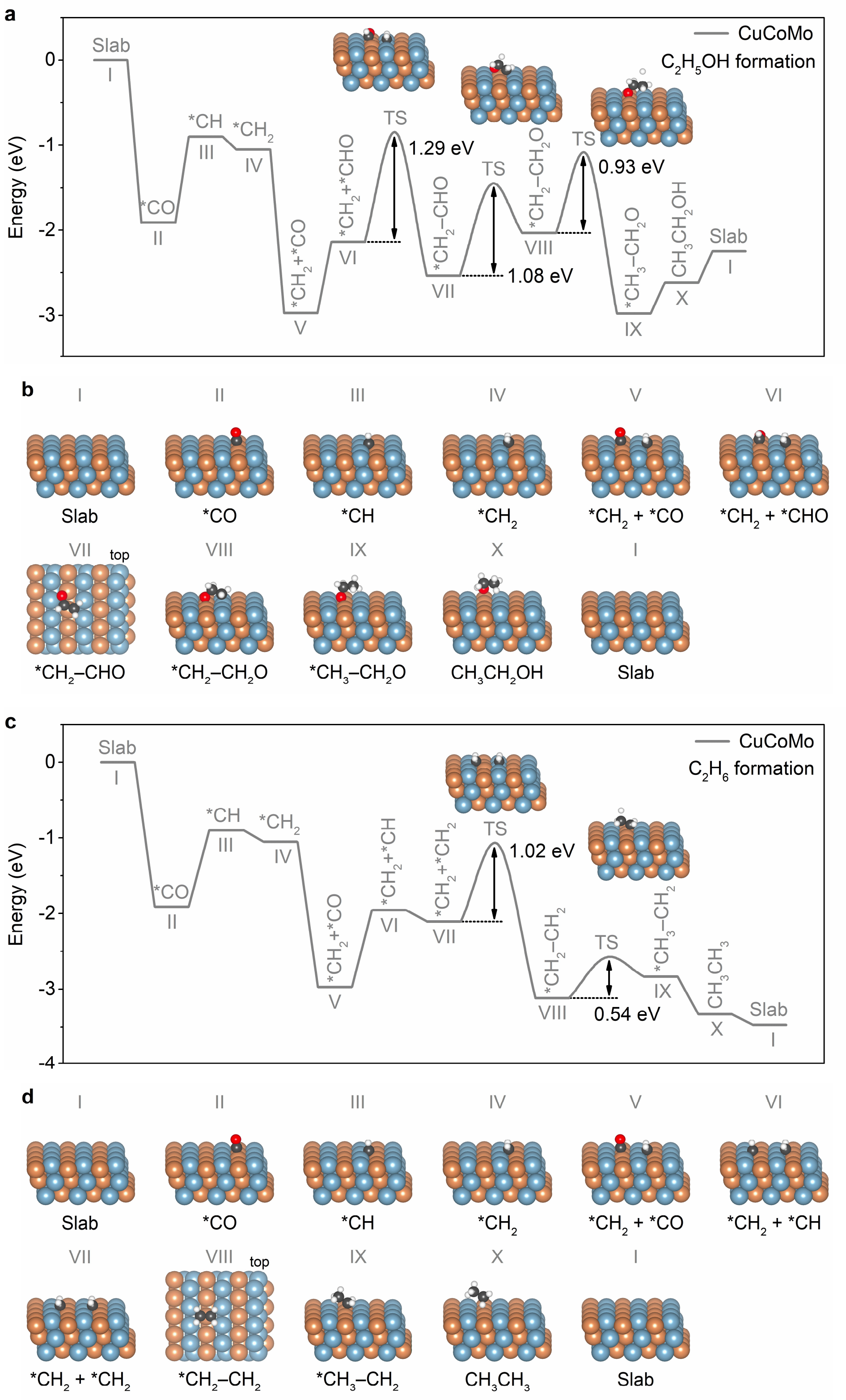

Fig. S20 | Reaction pathways on the CuCoMo catalyst. (a) Reaction pathway for C_2H_5OH formation. (b) Views of the adsorbed species for C_2H_5OH formation. (c) Reaction pathway for C_2H_6 formation. (d) Views of the adsorbed species for C_2H_6 formation. Va, carbon vacancy.

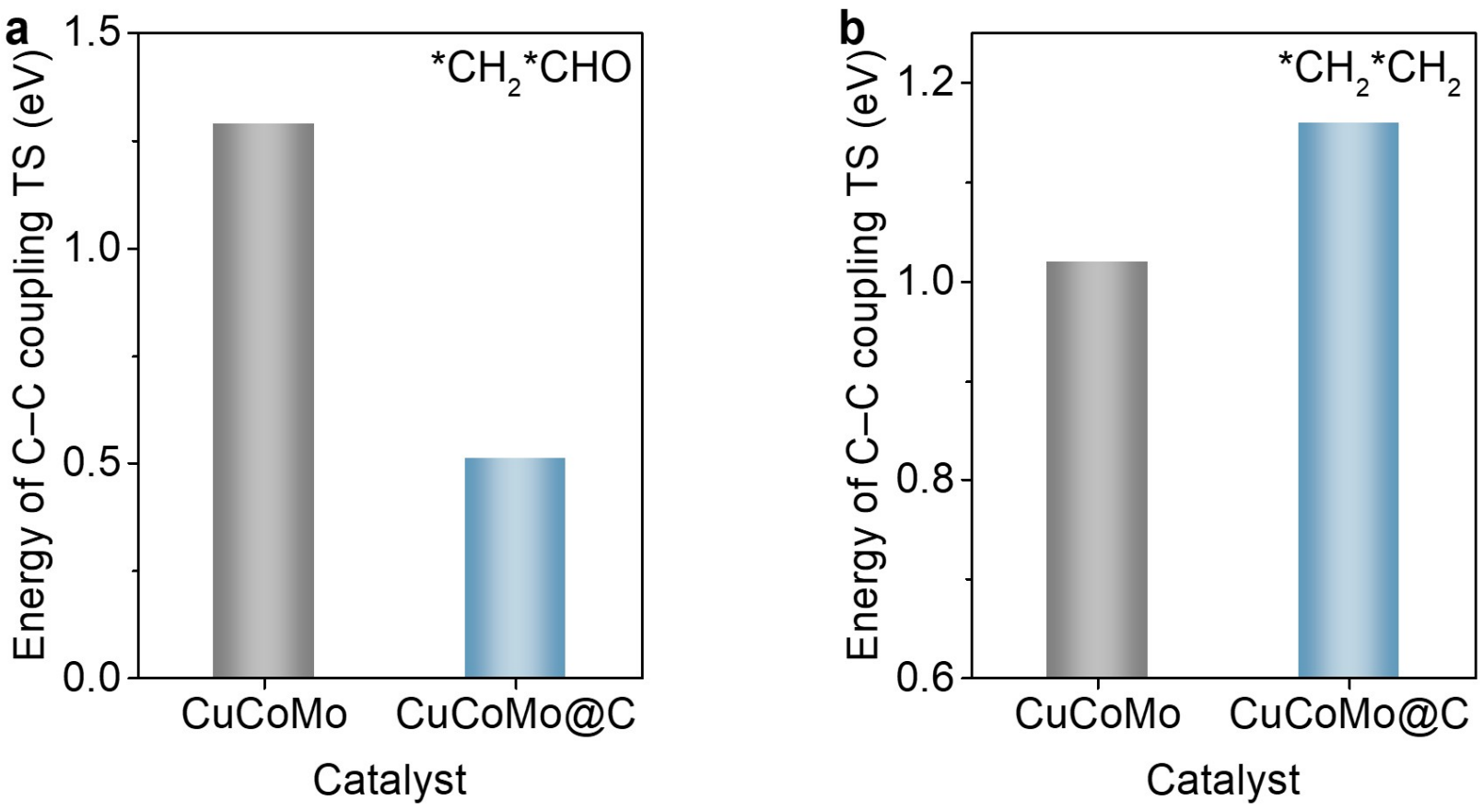

Fig. S21 | C–C coupling TS energies. (a) Energy barriers for the C–C coupling of co-adsorbed $*CH_2$ and *CHO intermediates. (b) Energy barriers for the C–C coupling of co-adsorbed $*CH_2$ and $*CH_2$ intermediates.

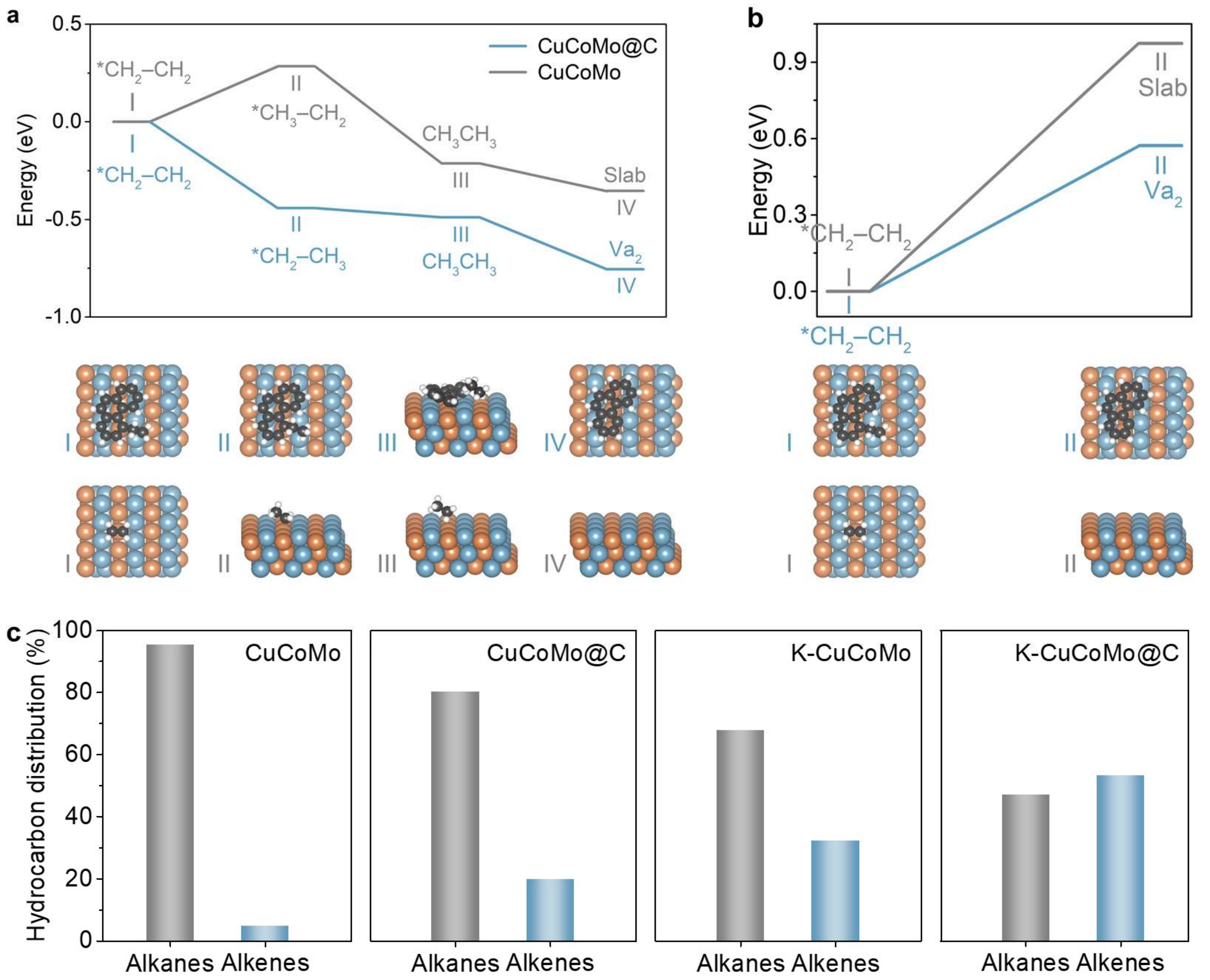

Fig. S22 | Reaction pathways of alkanes and alkenes on CuCoMo and CuCoMo@C surfaces. (a) Reaction pathway of ethylene hydrogenation to ethane. (b) Reaction pathway of ethylene desorption. (c) Hydrocarbon distribution of the catalysts. Reaction conditions: GHSV = 4 L g_{cat}^{-1} h^{-1}, T = 240 °C, P = 3.0 MPa, and H_2/CO = 2.

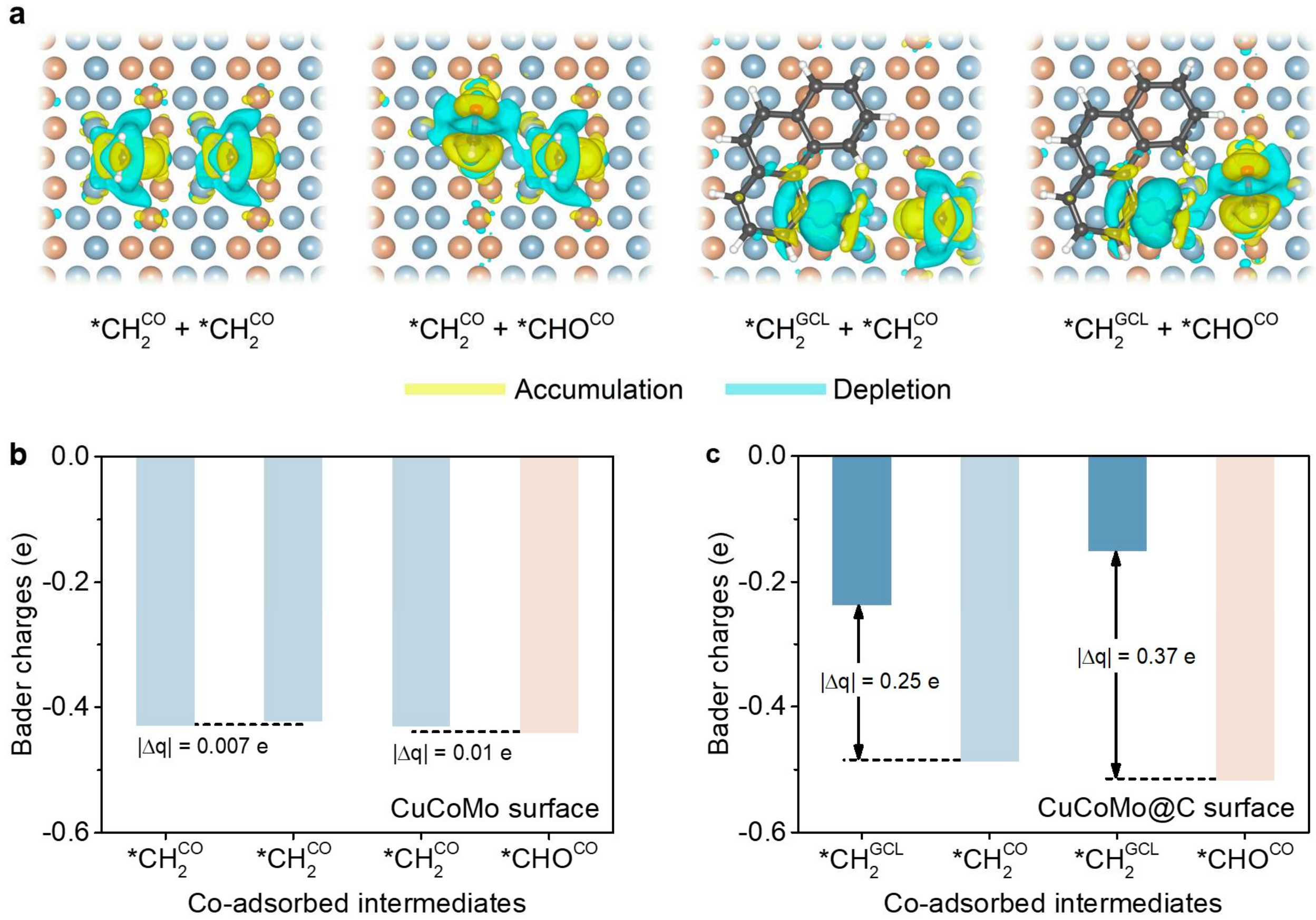

Fig. S23 | Charge distribution analyses of the intermediates. (a) Charge density differences of the co-adsorbed $*CH_2$ and *CHO intermediates on the CuCoMo and CuCoMo@C surfaces. (b) Bader charges of the co-adsorbed $*CH_2$ and *CHO intermediates on the CuCoMo surface. (c) Bader charges of the co-adsorbed $*CH_2$ and *CHO intermediates on the CuCoMo@C surface.

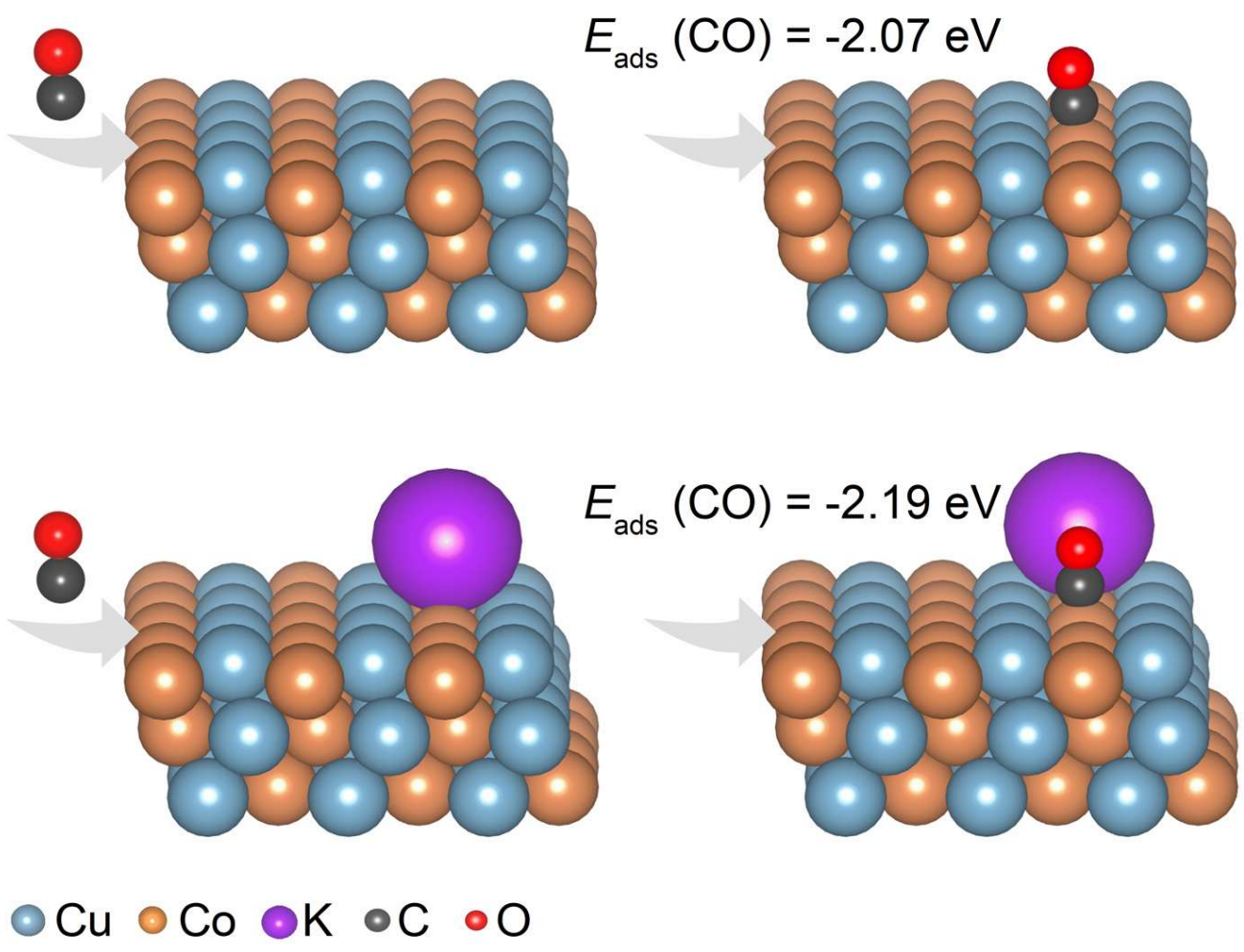

Fig. S24 | CO adsorption energies. (a) CO adsorption energy on the CuCo surface. (b) CO adsorption energy on the K-CuCo surface.

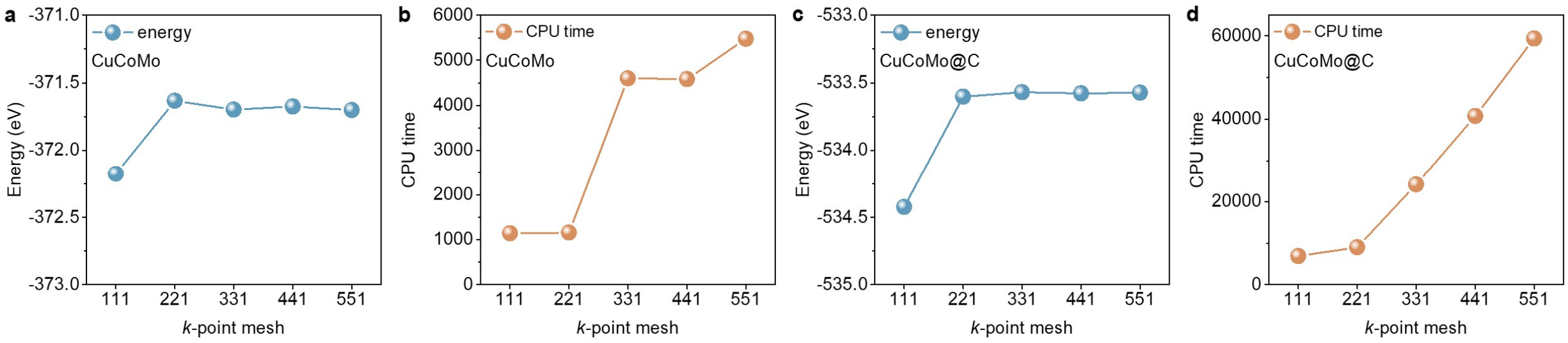

Fig. S25 | *k*-point convergence tests. (a) and (c) Calculated energies as a function of *k*-point sampling for the CuCoMo and CuCoMo@C models, respectively. (b) and (d) Corresponding CPU times required for the calculations.

Supplementary Tables

Table S1 | Catalytic performance data.

	**Temp. (°C)**	**Press. (MPa)**	**GHSV (L g_{cat}^{-1} h^{-1})**	X_{CO} **(%)**	S_{MeOH} (%)	S_{HAs}	$S_{CH^{=}}$	$S_{CH^{-}}$	S_{CO2}	$S_{ROH+CH^{=}}$	**C_{2+}OH/ROH (%)**	**$CH^{=}/CH^{-}$ (%)**	**Carbon balance**
β-Mo_2C	240	3.0	8	6.4	9.3	1.5	1.7	77.0	10.5	12.5	13.9	2.2	0.992
CuCoMo	240	3.0	8	15.2	16.2	16.0	2.6	53.9	11.3	34.8	49.7	4.6	1.053
CuCoMo@C	240	3.0	8	11.8	23.5	36.8	7.4	29.0	3.3	67.7	61.0	20.3	1.021
K-CuCoMo	240	3.0	8	6.3	16.6	35.3	7.9	17.7	22.5	59.8	68.0	30.9	1.003
K-CuCoMo@C	240	3.0	8	10.1	8.8	58.9	11.8	10.9	9.6	79.5	87.0	52.0	1.018

Reaction conditions: T = 240 °C, P = 3.0 MPa, GHSV = 8 L g_{cat}^{-1} h^{-1}, and H_2/CO = 2. ROH = total alcohols. MeOH = methanol. HAs = higher alcohols. CH^{-} = alkanes (including CH_4). $CH^{=}$ = alkenes. High-value products = ROH + $CH^{=}$.

Table S2 | Catalytic performance data for the catalysts studied.[a]

Catalyst[b]	Temp. (°C)	Press. (MPa)	GHSV[c] (L g_{cat}^{-1} h^{-1})	X_{CO} (%)	S_{MeOH}	S_{HAs}	$S_{CH^=}$	S_{CH^-}	S_{CO2}	$S_{(ROH+CH^=)}$	$C_{2+}OH/ROH$ (%)	$Yield_{HAs}$[e] (C %)	$Yield_{(ROH+CH^=)}$[e] (C %)
					(%)								
CuFe/CNF [(1)]	270	5.0	4	70	6	19	7	48	20	33	76	13	22
CuFe/CNF [(2)]	275	5.0	32	11	7	37	19	27	10	63	84	4	7
Co_1Fe_2 [(3)]	260	3.0	4.8	21	11	29	12	22	20	52	73	6	11
Co/AC [(4)]	220	3.0	2000 [d]	48	2	13	15	69	1	30	87	6	14
Na-Co/AC [(5)]	220	3.0	2	13	4	27	29	28	13	60	87	4	8
1.1Rh-CoMn [(6)]	220	6.0	2	33	1	32	22	26	19	55	97	11	18
CoMnLDH [(7)]	240	6.0	6000 [d]	18	3	17	20	59	2	39	85	3	7
Ca-Fe [(8)]	190	4.0	1.8	22	9	28	22	28	8	59	76	6	13
K_2CO_3/Mo_2C [(9)]	300	1.0	5000 [d]	13	5	21	4	23	47	30	81	3	4
Ni/K/Mo_2C@LC [(10)]	380	7.0	7	34	5	26	5	26	38	36	84	9	12
$Cu_{0.25}Co_{0.75}$ [(11)]	250	3.0	3900 [d]	71	2	11	–	86	–	13	85	8	9
CuFe/N-CNT [(12)]	220	2.0	6	21	8	19	–	72	1	27	70	4	6
$Cu_4Fe_1Mg_4$-MMO [(13)]	260	1.0	2.4	53	3	27	–	40	30	30	90	14	16
$Co_{4.7}Mo$@C [(14)]	275	3.0	15	48	3	9	0	67	21	11	82	4	5
Co_2Cu_1 [(15)]	240	4.0	7.2	7	9	25	13	51	2	47	74	2	3
Co-Fe-300-0.25 [(16)]	260	3.0	10.8	24	3	31	–	51	15	34	91	7	8
Au-Fe [(17)]	260	3.0	4.8	20	20	32	12	24	12	64	62	6	13
$CuCo/SiO_2$ [(18)]	240	3.0	4	6	12	15	4	65	4	32	56	1	2
CuCo/AC [(19)]	240	3.0	4	26	17	4	1	76	2	22	19	1	6
CuZnAl-comm [(20)]	280	5.0	4	29	66	10	0	11	13	76	13	3	–
K-CuCoMo@C (This work)	240	3.0	8	10	9	59	12	11	9	80	87	6	8
	285	3.0	4	81	4	33	7	18	38	43	89	27	35
	280	4.0	4	77	4	36	7	17	36	47	90	28	36
	280	5.0	4	80	4	37	6	18	35	47	90	30	38

[a]Reaction conditions: T = 240 °C, P = 3.0 MPa, GHSV = 4 L g_{cat}^{-1} h^{-1}, and H_2/CO = 2. [b](1–17) Data from references (refs.[38–53]); (18 and 19) In-house synthesised catalysts; (20) Commercial catalyst. [c]GHSV: L g_{cat}^{-1} h^{-1}. [d]GHSV: h^{-1}. ROH = total alcohols. MeOH = methanol. HAs = higher alcohols. CH^- = alkanes (including CH_4). $CH^=$ = alkenes. High-value products = ROH + $CH^=$. [e]Yield = product yield on a carbon-number basis.
