# Supplementary material for: Dynamic carbon overlayered CuCo alloy promotes syngas to higher alcohols
Source: Nat Commun. 2026 Sep 30;17:11239. doi: 10.1038/s41467-026-78203-7 (PMC13720033; doi:10.1038/s41467-026-78203-7)
Supplement: Supplementary file 2 [file 41467_2026_78203_MOESM2_ESM.pdf]

Peer Review File

Dynamic carbon overlayered CuCo alloy promotes syngas to higher alcohols

Corresponding Author: Dr Youzhu Yuan

This file contains all reviewer reports in order by version, followed by all author rebuttals in order by version.

Version 0:

Reviewer comments:

Reviewer #1

(Remarks to the Author)
Detailed revision has been made to further improve the manuscript and it can be considered for acceptance.

Reviewer #2

(Remarks to the Author)
The manuscript of Liu et al., relating to changes in catalytic selectivity when synthesising carbon overlayers on CuCo nanoparticles, has been reconsidered after transfer from another journal in the Springer Nature portfolio and appropriate revision. The authors are thanked for their efforts to clarify and improve the manuscript.

The results continue to be interesting and potentially impactful, as improved selectivity could have great impact for commercial processes. The manuscript content remains quite technical, the mechanistic demonstration of how the change in selectivity occurs is perhaps still lacking, and any clear demonstration of transferability to other chemical systems beyond the bespoke multi-element nanoparticles considered is also absent. I might therefore consider recommendation to a catalysis specific journal, where the readership will value the presentation and outcomes more strongly, but also can see the impact as warranting consideration for the current journal.

The following points arise when reviewing the provided response:
- The authors consider in some detail the composition of the CuCo nanoparticle (P31 of response) but fail to explain why, if the nanoparticle is completely covered in carbon overlayers, these changes affect reactivity. The authors explain that changes in the way Cu and Co interact with the reaction intermediates will determine the reactivity - but if Cu and Co are completely inaccessible, this is irrelevant. I still believe there needs to be some quantification of the number of Cu and Co sites accessible for the covered nanoparticles; this would then allow the reviewers and readers to understand fully what is the active site in the described catalysis - the Cu and Co surfaces, or the carbon overlayers. In the revised manuscript, the authors state the alloy surface is "covered by GCLs" (P4, L94), which implies no Cu or Co are accessible.
- For the modelling of the CuCo alloy surface, can the authors confirm what alloying arrangement was taken and why (P35 of response). It seems any mechanism that involves these alloy surfaces is heavily dependent on the alloy order, and so explanation of the choice is valuable for the reader. (I note and appreciate that the models have been made accessible online - thank you.)
- The graphs for testing of simulation settings (k-grids, force convergence) should be in the SI, but also the y-axis is inappropriately large when presenting energies - please zoom in to make much more visible, so the readers can see the magnitude of the change, or tabulate values.
- I appreciate the effort made to model the entire reaction mechanism (P36 of response). However, it would be reasonable to expect that transition states (kinetics) are also calculated for the entire reaction mechanism if we are to make any interpretation of catalytic reactivity/selectivity - the thermodynamics are insufficient to make conclusions of reactivity/selectivity. The impact of inappropriately incomplete study for the reaction mechanism is visible in Extended Data Fig 6/7, where conclusions are made for the single TS calculated - how do we know this is the rate determining step or selectivity determining step given the changes to the catalyst structure and surface when overlaying the carbon? I recognise performing these calculations are significant extra work, and so if these not to be included the authors need to provide a much more robust explanation of why this should be acceptable in the current work.

The following minor notes arise when re-reading the manuscript after the updates:
- The language could be still improved in places. Taking the first paragraph, the following parts are examples where improvements may be implemented:
-- define F-T on first use
-- "are difficult to achieving significant" doesn't make sense
-- "Because excessive" is inappropriate for a sentence start - could be "Excessive CO dissociation causes ..."
-- Some commas would make L40 more readable - e.g., deoxygenation, especially alkane formation, to ...
- Many acronyms are undefined further in the text, and should be defined when first used
- Fig 2, a, right, the differences in the two models is not clear
- L180-185: If it is possible to quantify the Cu or Co surface active sites available, a TON would be beneficial here.
- Fig 4 - "A plausible reaction" - "A possible reaction"? I'm not sure if we have confirmed it as being plausible?
- L284, L287, charge has units of e, please include.
- L289, please improve presentation of \DeltaE_TS to use subscript rather than underscore.
- L290, Bader charge transfer is normally denoted as \Delta q, not e?
- P12, a title for the conclusions might be valuable, as they appear abruptly.
- L515, the data availability should be included here (give link), or in separate data availability statement - currently this seems to be absent.
- Fig S13, this image is not well used and also poorly framed (as in, the image could be zoomed in on the surface configuration of K/CO)

Version 1:

Reviewer comments:

Reviewer #2

(Remarks to the Author)
The revisions and new data provided by the authors are very much appreciated, and many of my previous queries are very well addressed. Thank you.

There are two minor aspects that I think can just be slightly improved for the reader's benefit:

(i) In response to my prior comments, you answered QIII by listing computational settings and providing a statement that "These computational settings are standard and have been widely adopted in DFT studies". If they are standard, then it would be appropriate to cite where that standard is reported (noting there is no citation currently). However, for the k-point sampling, the provided settings (2x2x1) cannot be "standard" as the choice of this setting is dependent on the unit cell of the studied system. In the current manuscript, the super cell used for modelling is not explicitly stated, so the reader cannot try to make this assessment; but furthermore, k-space sampling is a numerically convergent parameter and therefore the validity of this choice should always be presented with a simple graph showing energy as a function of k-space sampling. Please provide such a plot in the SI to validate the accuracy of your setting.

For completeness, I also note that "electronic convergence criterion" is not specific - what exactly is being converged, please? And I also am inclined to think 0.05 eV/\AA is a relatively inaccurate geometry convergence parameter when reporting local minima/reaction barriers, and it would be valuable for the readers to know how e.g. Fig S21 changes when a more "accurate" (and equally standard) setting of 0.01 eV/\AA is applied.

(ii) I thank the authors for including the Data Availability Statement. However, the provided text does not aid the reader - it would be very valuable to actually state what data is provided, in what formats (i.e., metadata), so the reader can engage with and use the data as effectively as possible. Also consider links/citations to the data if suitable.

All other aspects of the revision and response are appreciated, with the additional work completed by the authors commendable.

Open Access This Peer Review File is licensed under a Creative Commons Attribution 4.0 International License, which permits use, sharing, adaptation, distribution and reproduction in any medium or format, as long as you give appropriate credit to the original author(s) and the source, provide a link to the Creative Commons license, and indicate if changes were made.
In cases where reviewers are anonymous, credit should be given to 'Anonymous Referee' and the source.
The images or other third party material in this Peer Review File are included in the article's Creative Commons license, unless indicated otherwise in a credit line to the material. If material is not included in the article's Creative Commons license and your intended use is not permitted by statutory regulation or exceeds the permitted use, you will need to obtain permission directly from the copyright holder.
To view a copy of this license, visit https://creativecommons.org/licenses/by/4.0/

REVIEWER COMMENTS

Reviewer #2

The manuscript of Liu et al., relating to changes in catalytic selectivity when synthesising carbon overlayers on CuCo nanoparticles, has been reconsidered after transfer from another journal in the Springer Nature portfolio and appropriate revision. The authors are thanked for their efforts to clarify and improve the manuscript.

The results continue to be interesting and potentially impactful, as improved selectivity could have great impact for commercial processes. The manuscript content remains quite technical, the mechanistic demonstration of how the change in selectivity occurs is perhaps still lacking, and any clear demonstration of transferability to other chemical systems beyond the bespoke multi-element nanoparticles considered is also absent. I might therefore consider recommendation to a catalysis specific journal, where the readership will value the presentation and outcomes more strongly, but also can see the impact as warranting consideration for the current journal.

Response: We sincerely thank you for the careful evaluation of our manuscript and for recognizing its potential impact on improving catalytic selectivity. We greatly appreciate the constructive comments. We also acknowledge the concerns regarding the mechanistic clarity, transferability, and broader significance of the work. In response, we have carefully checked and standardized the presentation, added new experiments, expanded and refined the DFT calculations to strengthen the mechanistic study, and improved the clarity of the manuscript throughout.

I. Mechanistic demonstration

We agree with you that a clearer mechanistic understanding of the selectivity change is essential. In our previous revision, we combined isotope-labeling experiments with operando/surface-sensitive characterizations and kinetic analyses, including TPSR, O_2–TPO, Raman, NAP–XPS, LEIS, and HRTEM. The combined results consistently demonstrate that graphitic carbon overlayers (GCLs) are not passive spectator layers, but are dynamically involved in catalysis. However, we agree that the previous version lacked a sufficiently clear quantitative link between these mechanistic insights and the observed selectivity trends. To address this point, we have now added CO–TPD and CO pulse adsorption measurements, together with O_2–TPO and Raman analyses, to quantitatively evaluate the

population of accessible metal-GCL interfacial active sites. **In the CuCo–GCL interface, GCL-derived species participate in C–C coupling, while neighboring CuCo sites activate CO/H_2 and generate CO-derived intermediates**. The results show that the GCL-regulated interfacial structure exhibits a similar evolution trend to higher alcohol (HA) selectivity, revealing that **the GCLs govern the genuine active structure responsible for C–C coupling**. Meanwhile, we optimized the catalyst model and performed new DFT calculations to strengthen the mechanistic investigation. Newly added transition-state (TS) calculations demonstrate that **GCL participation markedly lowers the barrier for coupling between GCL-derived *CH_2 and CO-derived *CHO**, thereby redirecting the reaction toward alcohol formation. In addition, the refined Bader charge analysis reveals the formation of an **asymmetric polarized coupling pair**, providing the electronic origin for the reduced C–C coupling barrier. Therefore, the revised manuscript now provides a clearer mechanistic explanation for how GCL evolution drives the selectivity shift toward HAs.

II. Transferability and generality

We appreciate your perspective regarding transferability of our findings. We fully agree that extension to other catalyst families is valuable. While the present manuscript focuses on CuCo-Mo_2C to establish the mechanism, the **central advance is the identification of a broader concept**: dynamic GCL-mediated reaction can actively steer product selectivity by promoting C–C coupling through carbon consumption/replenishment. To preliminarily evaluate the generality of this concept, we have extended the strategy to a CuFe-Mo_2C system and, by tuning the Mo_2C morphology and CuFe composition, synthesized a GCL-coated one dimensional CuFeMo_{1D}@C catalyst. Initial results show a similar enhancement in HA selectivity at lower temperatures (130–190 °C), together with analogous dependence of product distribution on GCL characteristics. Preliminary isotopic and spectroscopic analyses further support the participation of GCL-derived CH_x species and dynamic evolution under reaction conditions. These observations suggest that the mechanism is not restricted to the CuCo-Mo_2C system, but may be more broadly relevant to **metal-carbide-based catalysts capable of dynamic carbon migration and interface reconstruction** for promoting C–C coupling and steering product selectivity. We have also revised the manuscript to more explicitly emphasize that the strategy represents a general approach, rather than a system-specific phenomenon.

III. Suitability for the journal

We greatly appreciate your suggestion regarding journal scope. We would like to emphasize that:

Beyond catalyst optimization, this work introduces a mechanistic concept—dynamic graphitic carbon overlayers can function as active and tunable interfacial motifs that govern catalytic selectivity. This finding **establishes a structure–selectivity relationship at the interface level**, which is of broad interest to the catalysis and materials science communities. Considering the potential implication for syngas conversion and more broadly heterogeneous catalysis, we respectfully believe that the revised manuscript is well aligned with the broad readership of *Nature Communications*.

We thank you again for the constructive feedback, which has helped us significantly improve the clarity and impact of the manuscript. We hope that the revisions have adequately addressed the concerns.

The following points arise when reviewing the provided response:

QI. The authors consider in some detail the composition of the CuCo nanoparticle (P31 of response) but fail to explain why, if the nanoparticle is completely covered in carbon overlayers, these changes affect reactivity. The authors explain that changes in the way Cu and Co interact with the reaction intermediates will determine the reactivity - but if Cu and Co are completely inaccessible, this is irrelevant. I still believe there needs to be some quantification of the number of Cu and Co sites accessible for the covered nanoparticles; this would then allow the reviewers and readers to understand fully what is the active site in the described catalysis - the Cu and Co surfaces, or the carbon overlayers. In the revised manuscript, the authors state the alloy surface is "covered by GCLs" (P4, L94), which implies no Cu or Co are accessible.

Response: We sincerely thank you for this insightful and important comment. We fully agree that clarifying the accessibility of active sites is essential for understanding the catalytic origin of the observed selectivity changes. We would like to clarify that the GCLs partially cover the CuCo nanoparticles, forming CuCo–GCL interfaces with defective interfacial carbon sites (Raman, TEM, contact angle), rather than forming a continuous carbon shell that completely blocks the metallic surface.

To address the request for quantification, we have added CO pulse chemisorption and CO–TPD to analyse the number and nature of accessible adsorption sites (**Fig. S13, Fig. 2c**), together with O_2–TPO and Raman to quantify carbon amount and GCL defect density. These results confirm substantial CO uptake on the covered catalysts, directly demonstrating that accessible adsorption sites remain

after GCL formation. Importantly, both the population and properties of these CO-accessible sites vary systematically with carbon amount. Based on these results, we now distinguish two adsorption types in the revised manuscript:

1.Moderately adsorbed CO, reasonably attributed to CuCo–GCL interfacial sites (CuCo–GCL_{int}), including defective interfacial carbon sites at the CuCo–GCL interface (CuCo–$GCL_{def\text{-}int}$) that generate GCL-derived CH_x species and participate in C–C coupling reactions.

2. Strongly adsorbed CO, attributed to GCL-modified CuCo sites (CuCo–GCL_{mod}) that remain accessible to CO/H_2 and generate reaction CO-derived intermediates.

Most importantly, when the abundance of these quantified interfacial sites is incorporated into a descriptor—Dynamic GCL Coupling Index (DGCI)—its variation with carbon amount exhibits a highly consistent volcano-type trend with the experimentally observed HA selectivity ($DGCI = CuCo\text{–}GCL_{def\text{-}int} \times CuCo\text{–}GCL_{mod}$). Moderate carbon deposition maximizes effective CuCo–GCL interfaces and favors HA formation, whereas excessive carbon coated partially masks interfacial sites, lowers metal accessibility, and/or introduces mass-transfer limitations. **This provides quantitative evidence that defective interfacial carbon sites at the CuCo–GCL interface are the genuine active structures responsible for C–C coupling.** Therefore, the catalytically relevant surface is neither bare CuCo nor isolated carbon overlayers, but a cooperative metal-GCL interfacial architecture formed by partial carbon coverage, in which accessible CuCo sites generate CO-derived intermediates, while adjacent defective carbon interfacial sites generate GCL-derived CH_x species and promote selective C–C coupling.

We have revised the wording throughout the manuscript accordingly and added the new quantitative analyses to make active-site identity explicit.

Action Taken:

1. Revisions in Figures

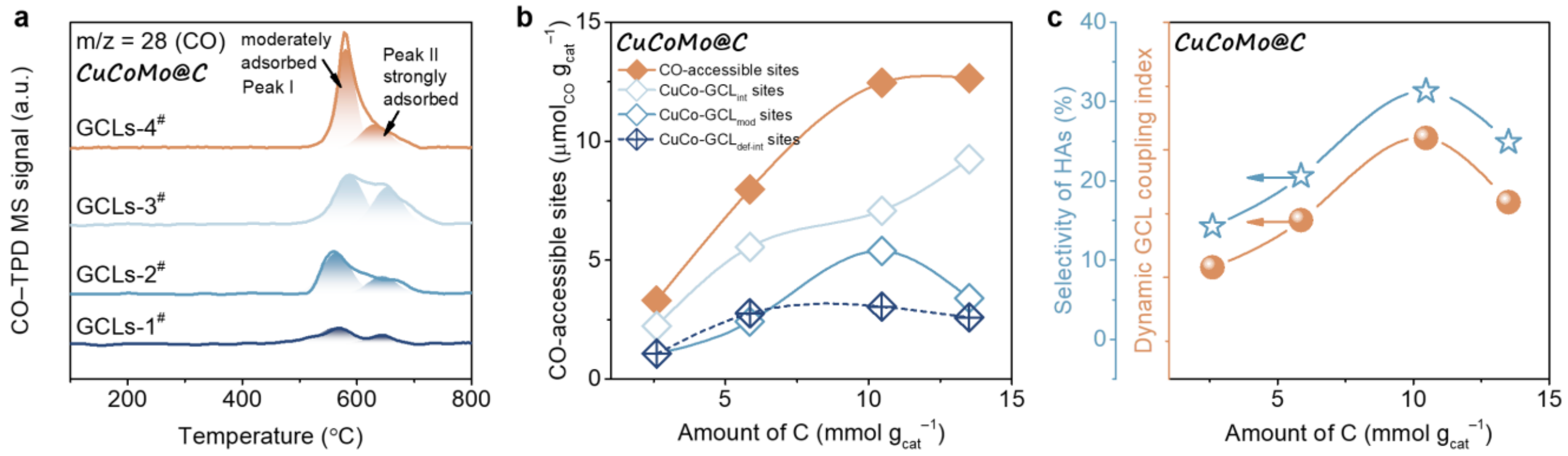

Fig. S13 | Quantification of carbon-overlayer-regulated interfacial adsorption sites. (a) CO–TPD (m/z = 28) profiles of the CuCoMo@C catalysts with different carbon amounts. Two characteristic desorption features are identified: Peak I at lower desorption temperatures, assigned to moderately adsorbed CO on CuCo–GCL interfacial sites, and Peak II at higher desorption temperatures, assigned to strongly adsorbed CO on GCL-modified CuCo sites. (b) Quantitative evolution of CO-accessible sites as a function of carbon amount. The total CO-accessible sites were determined by CO pulse adsorption. Based on TPD peak fitting and complementary Raman analyses, the accessible sites are further categorized as follows: CuCo–GCL$_{int}$ sites (CuCo–GCL interfacial sites), CuCo–GCL$_{def\text{-}int}$ sites (defective interfacial carbon sites at the CuCo–GCL interface that participate in C–C coupling reactions; $CuCo\text{–}GCL_{def\text{-}int} = CuCo\text{–}GCL_{int} \times I_D/I_G$), CuCo–GCL$_{mod}$ sites (GCL-modified CuCo sites that remain accessible to CO/H_2 and generate CO-derived intermediates). (c) Correlation between the HA selectivity and the Dynamic GCL coupling index (DGCI) as a function of carbon amount. $DGCI = CuCo\text{–}GCL_{def\text{-}int} \times CuCo\text{–}GCL_{mod}$. Reaction conditions: GHSV = 4 L g$_{cat}^{-1}$ h^{-1}, T = 240 °C, P = 3.0 MPa, and H_2/CO = 2.

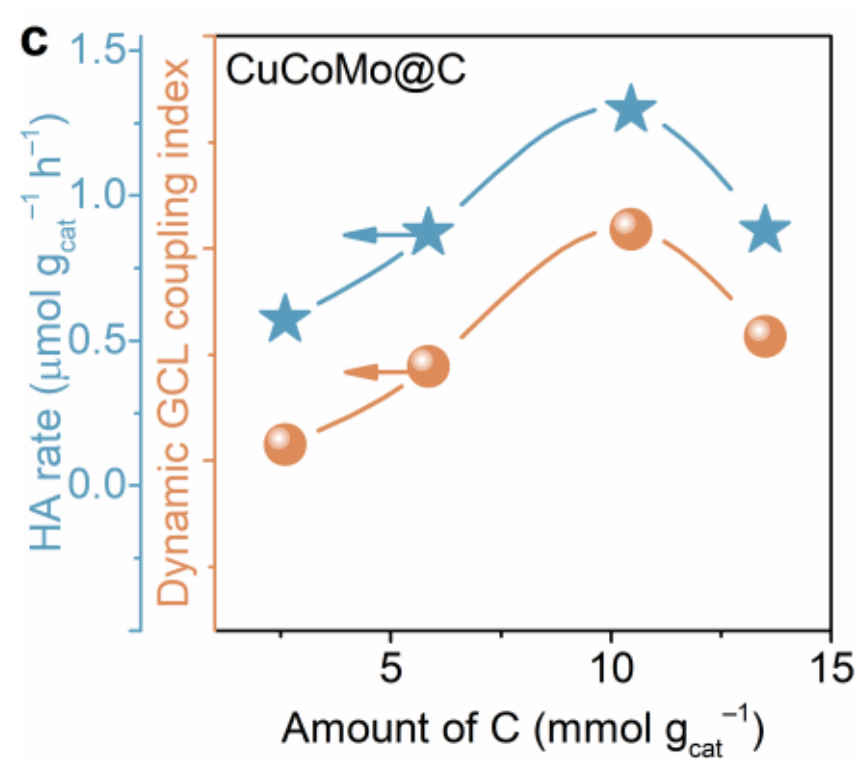

Fig. 2 | Effects of the GCLs on catalytic performance. **(c)** Correlation between the HA formation rate and the dynamic GCL coupling index (DGCI) as a function of carbon amount. DGCI serves as a semi-quantitative descriptor of the combined contributions of GCL-regulated intermediate-

generation and C–C coupling sites, defined as $DGCI = CuCo–GCL_{def\text{-}int} \times CuCo–GCL_{mod}$, where CuCo–$GCL_{def\text{-}int}$ is estimated as $CuCo–GCL_{int} \times I_D/I_G$. CuCo–$GCL_{int}$: CuCo–GCL interfacial sites, identified from moderately adsorbed CO species. CuCo–$GCL_{def\text{-}int}$: defective interfacial carbon sites at the CuCo–GCL interface. CuCo–GCL_{mod}: GCL-modified CuCo sites, associated with strongly adsorbed CO species.

2. Revision in Main Text

On page 7 and 8

"The carbon amount was quantified from O_2–temperature-programmed oxidation (O_2–TPO) by monitoring O_2 consumption using the m/z = 32 signal, revealing a gradual increase in carbon amount with increasing GCL thickness (Fig. S12a, b). Raman spectra show that at a moderate carbon amount, the elevated I_D/I_G ratio indicates a higher density of carbon defects (Fig. S12c).

To further clarify the structure of GCL-covered catalyst surface, CO chemisorption was employed to probe the number and nature of accessible adsorption sites. The results show that substantial CO uptake is retained even after the formation of GCLs, indicating that the GCLs do not completely block the metallic surface, but instead create more adsorption environments. More importantly, as the carbon amount and defect density vary, the adsorption properties of these sites evolve systematically, suggesting that the GCLs are not merely spectator species, but actively regulate the surface structure (Fig. S13a). Based on their adsorption behavior, two major types of CO species can be distinguished. The first corresponds to moderately adsorbed CO species, reasonably assigned to CuCo–GCL interfacial sites (CuCo–GCL_{int}), including defective interfacial carbon sites. The second corresponds to strongly adsorbed CO species, attributed to GCL-modified CuCo sites (CuCo–GCL_{mod}), which remain accessible for CO/H_2 activation and generate CO-derived intermediates (Fig. S13b). Notably, when the abundance of these quantified interfacial sites is incorporated into the GCL coupling index, its dependence on carbon amount follows a volcano-type trend that closely matches the selectivity and formation rate of HAs (Fig. 2c, Fig. S13c). This strong correlation suggests that the catalytically relevant structure originates from the synergistic CuCo–GCL interface, rather than bare CuCo surfaces or isolated GCLs alone. Moderate carbon deposition optimizes the effective metal–GCL interfacial structure, thereby promoting HA synthesis and leading to a prolonged induction period. In contrast, excessive carbon coverage reduces the defective interfacial sites, lowers metal accessibility, suppresses alcohol formation, and enables the reaction to reach equilibrium more rapidly (Fig. S12d). In addition, post-reaction Raman spectra reveal an increase in carbon defect density within the GCLs, accompanied by pronounced structural evolution (Fig. 2d). Together, these results suggest that defective interfacial carbon sites at the CuCo–GCL interface (CuCo–$GCL_{def\text{-}int}$) serve as the active

centers for C–C coupling, while the GCLs likely undergo dynamic structural evolution under reaction conditions."

QII. For the modelling of the CuCo alloy surface, can the authors confirm what alloying arrangement was taken and why (P35 of response). It seems any mechanism that involves these alloy surfaces is heavily dependent on the alloy order, and so explanation of the choice is valuable for the reader. (I note and appreciate that the models have been made accessible online - thank you.)

Response: We sincerely thank you for the attention given to the computational model and for recognizing our effort to make the structures openly accessible.

We fully agree that the atomic arrangement of the CuCo alloy can influence the calculated energetics. To provide a more reliable thermodynamic and kinetic description of the reaction pathway, we reconstructed and optimized the model for the expanded mechanistic study. The optimized model adopts an fcc (1 1 1) surface with alternating Cu and Co atomic rows, while preserving the Cu:Co ratio of 1:1. This model was selected for several reasons. First, our experimental characterizations indicate alloy between Cu and Co rather than separated monometallic phases. The overall Cu:Co ratio is also consistent with the composition of the experimentally optimized catalyst, which exhibited the best performance for HA synthesis. Second, XRD results indicate an fcc-type metallic phase, for which the (1 1 1) facet is the dominant exposed surface.

All DFT calculations, including the previous results and the newly added TS calculations, were performed using the optimized model to ensure a consistent thermodynamic and kinetic analysis throughout the reaction pathway.

Although the calculated data may vary to some extent with different alloy arrangements, our primary objective is to establish an experimentally representative CuCo surface that enables direct comparison between the bare metal surface and the GCL-modified interface. Importantly, the central mechanistic conclusion remains unchanged: the dynamic GCL-derived CH_x species that create an asymmetrically polarized interfacial environment, which facilitates C–C coupling with activated CO-derived intermediates.

To improve clarity, we have revised the manuscript to clarify the CuCo model structure and its selection rationale. The DFT calculation methods section has also been updated accordingly.

Action Taken:

Revision in Main Text

On page 15

DFT calculation methods

"Based on the catalyst synthesis and experimental characterizations, the CuCo structure was modeled as an fcc CuCo (1 1 1) slab with alternating Cu and Co atomic rows and a Cu:Co ratio of 1:1. The spin-polarized density functional theory (DFT) calculations were performed using the Vienna ab initio simulation package (VASP) based on the projector-augmented wave (PAW) method with a plane-wave basis set. The exchange-correlation functional was described using the generalized gradient approximation (GGA) with the Perdew–Burke–Ernzerhof (PBE) functional. A vacuum layer of approximately 15 Å was introduced to eliminate interactions between periodic images. The plane-wave energy cutoff was set to 450 eV, and van der Waals interactions were included using the DFT-D3 correction. A Monkhorst–Pack *k*-point mesh of 2 × 2 × 1 was employed for structural optimization. All structures were fully relaxed until the residual force on each atom was below 0.05 eV $Å^{-1}$, with the electronic energy convergence criterion set to 10^{-5} eV.

The Gibbs free energy change (ΔG) for each elementary step was calculated as

$$\Delta G = \Delta E + \Delta E_{ZPE} - T\Delta S + \Delta G_{pH} \quad (1)$$

where ΔE, ΔE_{ZPE}, and ΔS represent the changes in total energy, zero-point energy, and entropy, respectively; T is 298.15 K. ΔG_{pH} is the pH correction to the Gibbs free energy, and the pH was set to zero throughout this work.

The adsorption energy (ΔE_{ads}) was calculated according to

$$E_{ads} = E_{total} - E_{slab} - E_{adsorbate} \quad (2)$$

where E_{total} is the total energy of the adsorption system, E_{slab} is the energy of the clean slab, and $E_{adsorbate}$ is the energy of the isolated adsorbate.

QIII. The graphs for testing of simulation settings (*k*-grids, force convergence) should be in the SI, but also the y-axis is inappropriately large when presenting energies - please zoom in to make much more visible, so the readers can see the magnitude of the change, or tabulate values.

Response: We sincerely thank you for this helpful suggestion. Since the catalyst model has been reconstructed and all DFT calculations have been repeated using the optimized model, the previous convergence tests are no longer applicable. Instead, we have revised the DFT calculation methods section to clearly specify all computational parameters adopted throughout this work, including the plane-wave energy cutoff (450 eV), Monkhorst–Pack *k*-point mesh (2 × 2 × 1), force convergence

criterion (0.05 eV Å^{-1}), and electronic convergence criterion (10^{-5} eV). These computational settings are standard and have been widely adopted in DFT studies. All calculations in the revised manuscript were performed consistently using these computational parameters.

QIV. I appreciate the effort made to model the entire reaction mechanism (P36 of response). However, it would be reasonable to expect that transition states (kinetics) are also calculated for the entire reaction mechanism if we are to make any interpretation of catalytic reactivity/selectivity - the thermodynamics are insufficient to make conclusions of reactivity/selectivity. The impact of inappropriately incomplete study for the reaction mechanism is visible in Extended Data Fig 6/7, where conclusions are made for the single TS calculated - how do we know this is the rate determining step or selectivity determining step given the changes to the catalyst structure and surface when overlaying the carbon? I recognise performing these calculations are significant extra work, and so if these not to be included the authors need to provide a much more robust explanation of why this should be acceptable in the current work.

Response: We sincerely thank you for this insightful comment and fully agree that kinetic analysis is essential for interpreting catalytic reactivity and selectivity. In response, we have substantially expanded the DFT study by optimizing the catalyst model and performing additional TS calculations for the key steps governing product selectivity.

Specifically, TS calculations were added for the competing C–C coupling pathways together with the subsequent hydrogenation steps involved in C_2 product formation (**Fig. S19–21, and Fig. 4**). For the bare CuCoMo surface, the $*CH_2/*CH_2$ and $*CH_2/*CHO$ coupling steps exhibit the highest activation barriers along their respective reaction pathways, indicating that C–C coupling is the kinetically most demanding step and the key branching point governing product selectivity. Both coupling barriers are relatively high, reflecting the intrinsic difficulty of C_2 product formation on the metallic surface. Moreover, the TS for CO-derived $*CH_2/*CH_2$ coupling is lower than that for $*CH_2/*CHO$ coupling, indicating that hydrocarbon formation is kinetically favoured over alcohol formation. However, on the CuCoMo@C surface, GCL participation markedly lowers the barrier for coupling between GCL-derived $*CH_2$ and CO-derived $*CHO$ while increasing that for $*CH_2$ and $*CH_2$ coupling, thereby redirecting the reaction toward alcohol formation. The newly added TS calculations show that the slightly higher activation barrier in the late-stage hydrogenation step is associated with the extraction

of a carbon atom from the GCLs, reflecting the dynamic participation of the GCLs. Although carbon extraction is involved in both the alcohol and hydrocarbon pathways, the associated energetic penalty is considerably smaller for C_2H_5OH formation than for C_2H_6 formation.

Despite not every elementary step was treated by explicit TS calculations, the newly added key TS encompass the kinetically relevant steps governing product selectivity and the dynamic participation of the GCLs. Together with the refined Bader charge analysis, these results provide a substantially strengthened kinetic basis for the proposed reaction mechanism.

The relevant mechanistic discussion has been revised accordingly in the manuscript.

Action Taken:

Revisions in Figures

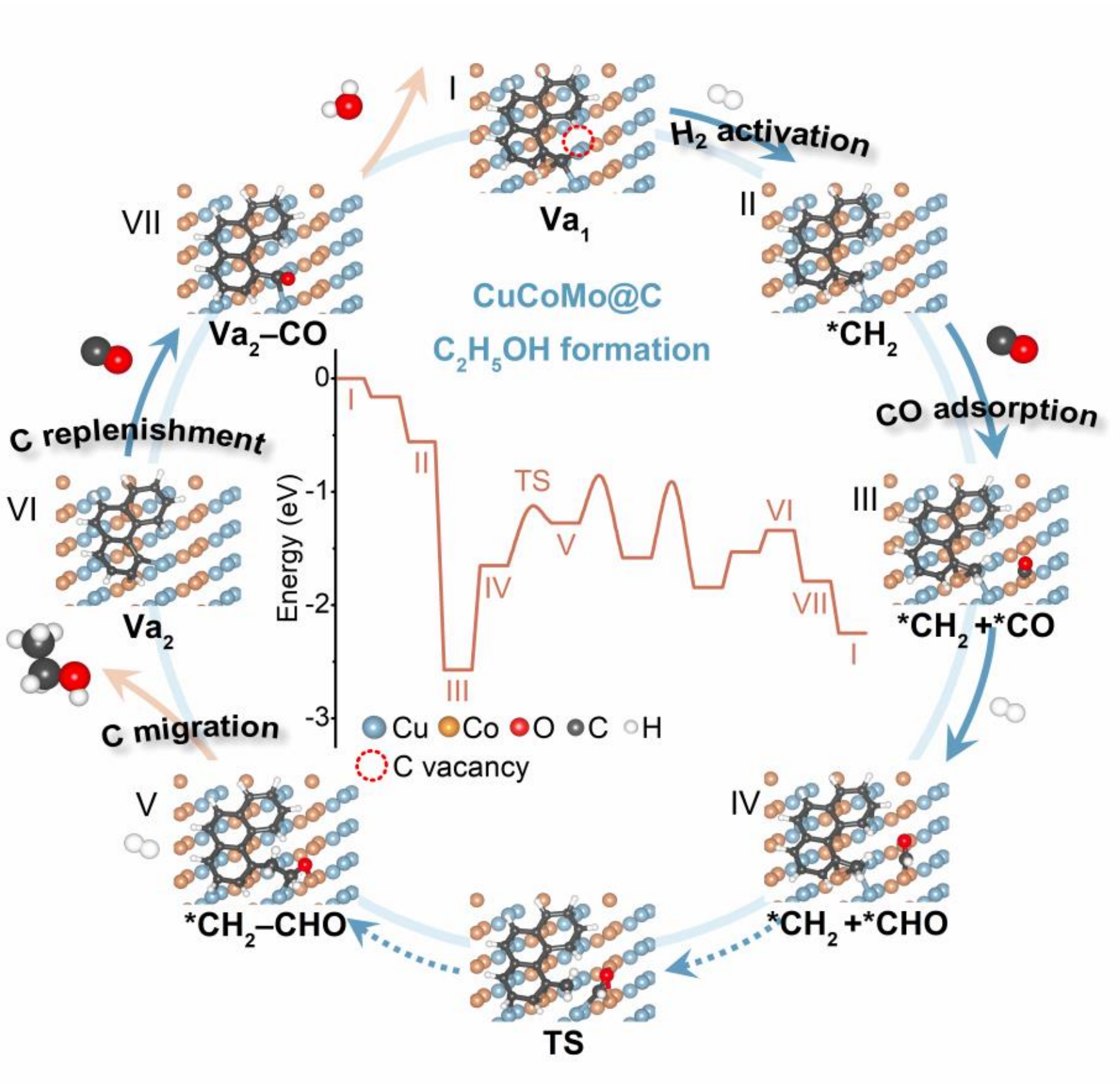

Fig. 4 | Dynamic GCL participation mechanism. Reaction pathway for ethanol formation over CuCoMo@C.

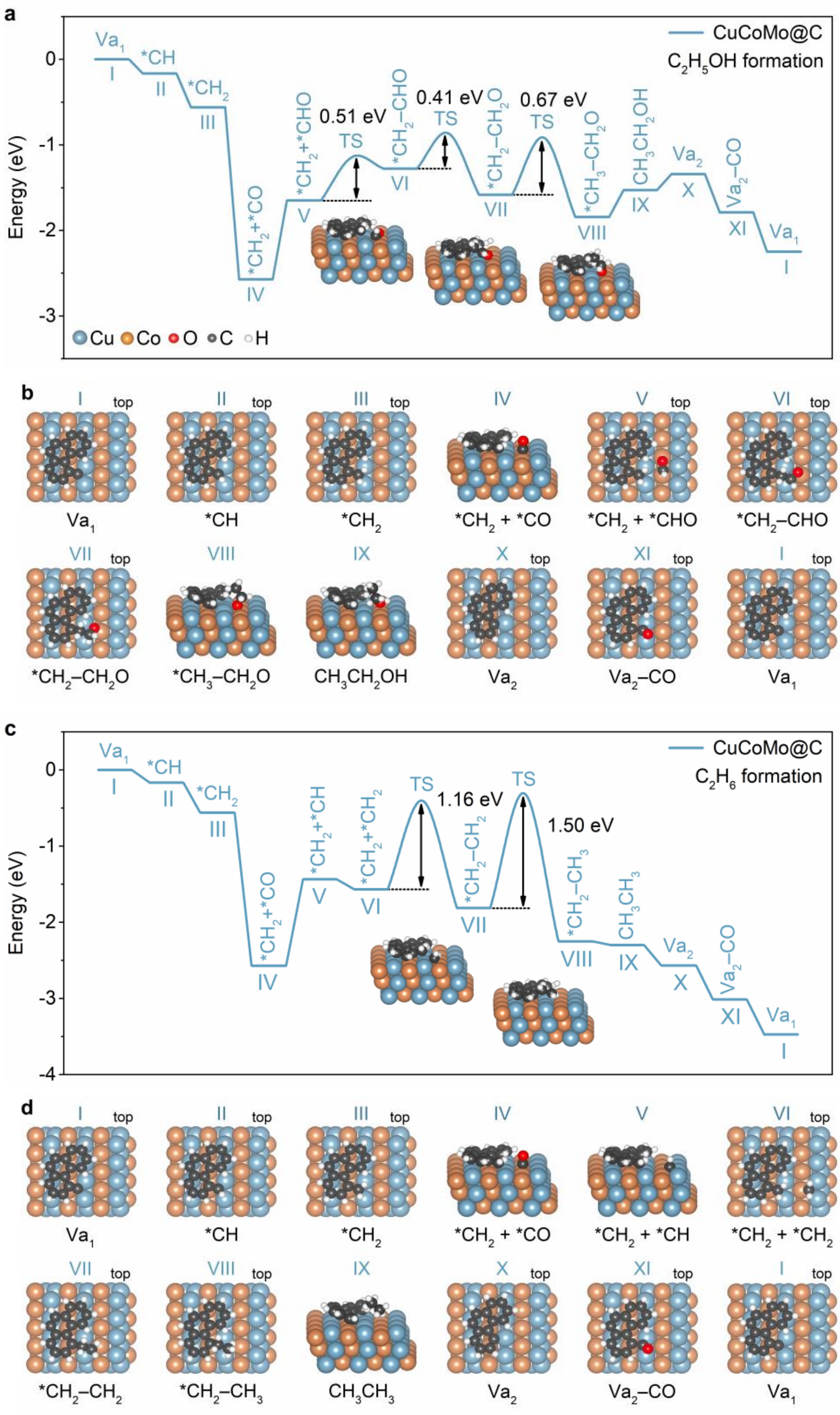

Fig. S19 | Reaction pathways on the CuCoMo@C catalyst. (a) Reaction pathway for C_2H_5OH formation. (b) Views of the adsorbed species for C_2H_5OH formation. (c) Reaction pathway for C_2H_6 formation. (d) Views of the adsorbed species for C_2H_6 formation. Va, carbon vacancy.

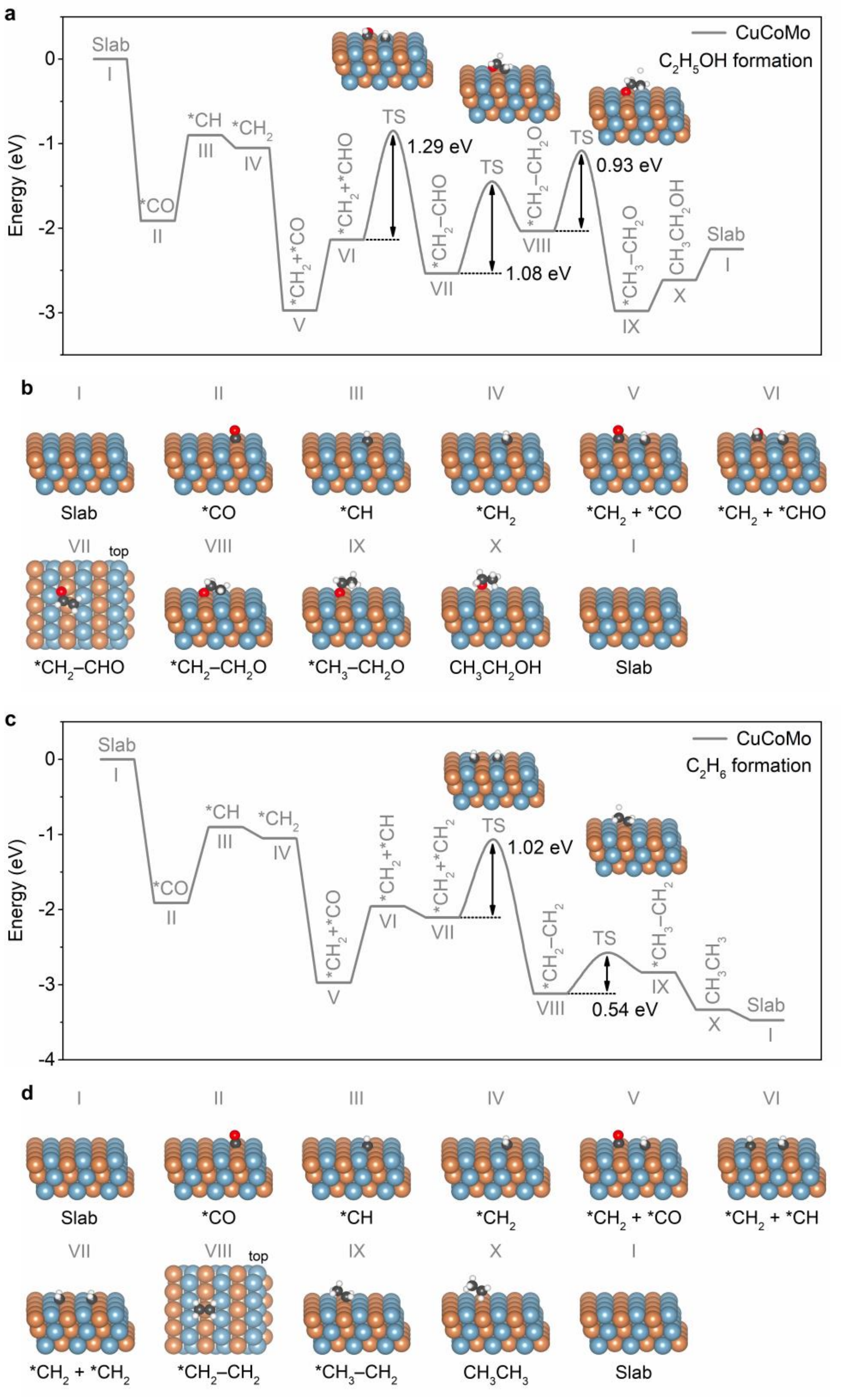

Fig. S20 | Reaction pathways on the CuCoMo catalyst. (a) Reaction pathway for C_2H_5OH formation. (b) Views of the adsorbed species for C_2H_5OH formation. (c) Reaction pathway for C_2H_6 formation. (d) Views of the adsorbed species for C_2H_6 formation. Va, carbon vacancy.

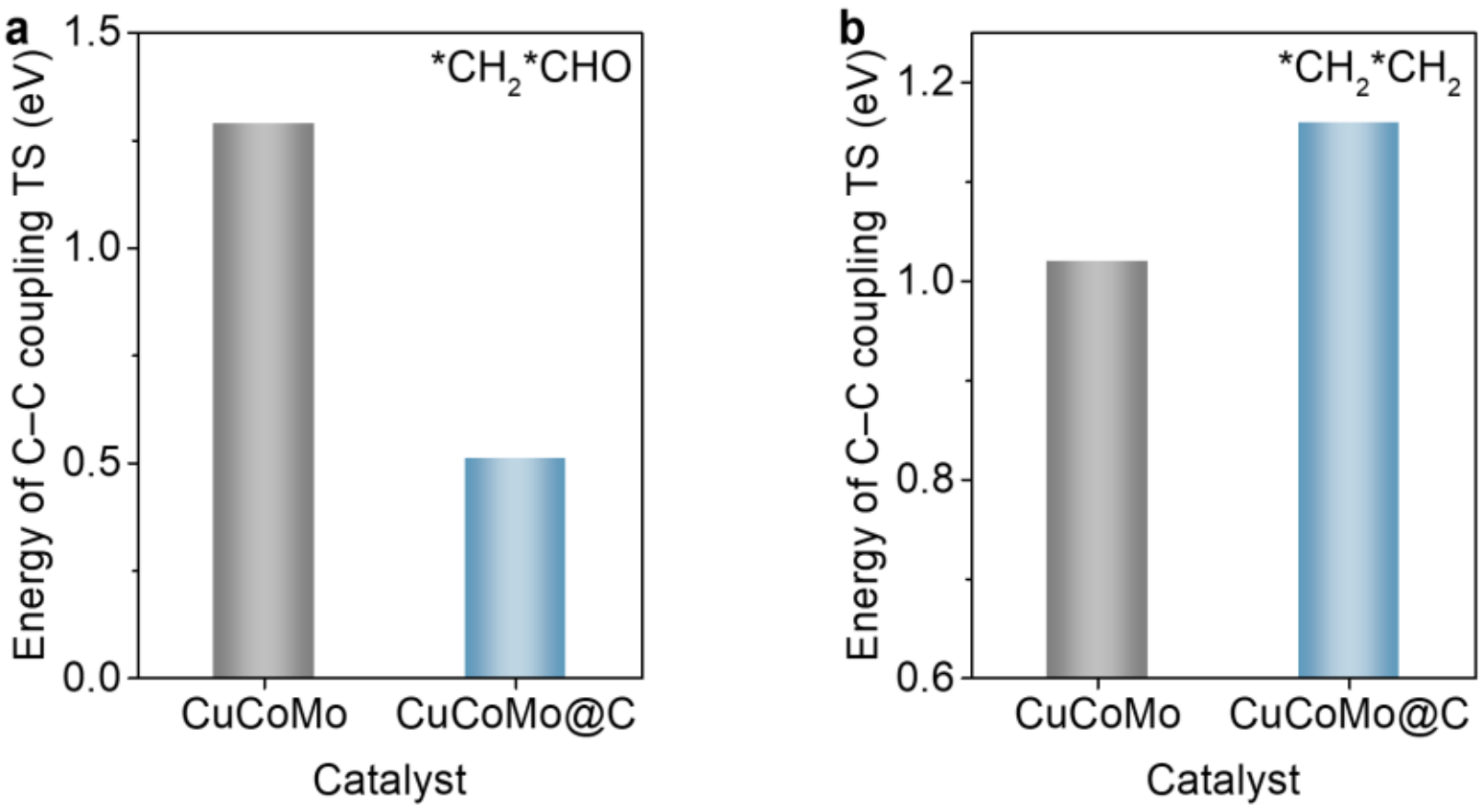

Fig. S21 | C–C coupling TS energies. (a) Energy barriers for the C–C coupling of co-adsorbed *CH_2 and *CHO intermediates. (b) Energy barriers for the C–C coupling of co-adsorbed *CH_2 and *CH_2 intermediates.

Revision in Main Text

On page 11

"To gain mechanistic insight into the dynamic participation of GCLs, density functional theory (DFT) calculations were performed. As shown in Fig. 4, the reaction on CuCoMo@C is initiated by hydrogenation of defective interfacial carbon species at the CuCo-GCL interface, generating GCL-derived *CH_2 species. Meanwhile, CO adsorbed on adjacent CuCo sites is hydrogenated to form CO-derived *CHO intermediate. The co-adsorbed *CH_2/*CHO subsequently undergo C–C coupling to form *CH_2–CHO, followed by successive hydrogenation to *CH_2–CH_2O, *CH_3–CH_2O and finally C_2H_5OH (Fig. S19a, b). On the CuCoMo surface, all intermediates originate exclusively from CO activation, and the selectivity toward C_2 products is determined by the competition between *CH_2/*CHO and *CH_2/*CH_2 coupling[11, 43], which constitute the highest kinetic barriers for oxygenate and hydrocarbon formation, respectively (Fig. S20). The transition-state (TS) barrier for *CH_2/*CHO coupling (1.29 eV) is higher than that for *CH_2/*CH_2 coupling (1.02 eV), favoring alkane formation. Remarkably, participation of GCLs fundamentally alters this competition. On CuCoMo@C, the barrier for *CH_2/*CHO coupling is substantially reduced to 0.51 eV, whereas that for *CH_2/*CH_2 coupling increases to 1.16 eV, making *CH_2–CHO the preferred C–C coupling intermediate (Fig. S21). Compared with the bare metal surface, direct *CH_2–CH_2 desorption is thermodynamically more favorable in the presence of the GCLs, further suppressing alkane formation (Fig. S22). The slightly increased barrier is associated with the extraction of a GCL carbon atom during the late-stage hydrogenation step, reflecting the dynamic participation of the GCLs. Upon

C_2H_5OH desorption, the resulting interfacial carbon vacancy is replenished by gaseous CO[44], thereby completing the dynamic carbon reconstruction cycle (Video S1)."

QV. The following minor notes arise when re-reading the manuscript after the updates:

The language could be still improved in places. Taking the first paragraph, the following parts are examples where improvements may be implemented:

Response: We sincerely thank you for your careful reading of the manuscript and helpful language suggestions. We have revised the manuscript accordingly. In the revised manuscript, **language edits are marked with gray shading**, while the changes made in **response to your comments are highlighted in yellow** for identification.

1. define F-T on first use

We thank you for this suggestion. Fischer–Tropsch (F–T) was already defined at its first appearance in the Abstract and Main test. We have nevertheless rechecked the manuscript and ensured consistent definition upon first use throughout the revised version.

Revisions in Main Text

On page 1

"Syngas (CO/H_2), sourced from coal, biomass, and shale gas, plays a crucial role in producing a wide range of industrially essential hydrocarbons and oxygenates through Fischer–Tropsch (F–T) synthesis and its variations."

On page 2

"Syngas (CO/H_2), derived from coal, biomass, and shale gas, holds significant potential for its transformation into a wide array of industrially vital hydrocarbons and oxygenates through Fischer–Tropsch (F–T) synthesis and related processes[1–3]."

2. "are difficult to achieving significant" doesn't make sense

We thank you for pointing out this language issue. The sentence has been revised for grammatical accuracy and clarity as follows:

Revision in Main Text: "However, making significant breakthroughs remains a formidable challenge due to the difficulty in improving HA selectivity while maintaining long-term catalyst stability[15,16]."

3. "Because excessive" is inappropriate for a sentence start - could be "Excessive CO dissociation causes ..."

We thank you for this helpful suggestion. We agree that the original sentence was incomplete and have revised it for grammatical accuracy and clarity as follows:

Revision in Main Text: "Excessive CO dissociation causes an imbalance between CH_x hydrogenation and CO insertion, leading to undesirable alkanes as the dominant products."

4. Some commas would make L40 more readable - e.g., deoxygenation, especially alkane formation, to ...

We appreciate your careful observation. We agree that the original sentence could be improved for readability and clarity. The sentence has been revised as follows:

Revision in Main Text: "Therefore, mitigating the adverse effects associated with CO deoxygenation, especially alkane formation, is crucial for improving catalytic performance in syngas-to-HA conversion."

5. Many acronyms are undefined further in the text, and should be defined when first used

We appreciate your comment. All acronyms have been carefully checked and are now defined at their first use in the revised manuscript.

Revisions in Main Text:

"**Syngas (CO/H_2)**, derived from coal, biomass, and shale gas, holds significant potential for its transformation into a wide array of industrially vital hydrocarbons and oxygenates through **Fischer–Tropsch (F–T)** synthesis and related processes[1–3]."

"Such conversions enable the production of essential compounds such as aromatics, acids, and olefins, as well as **higher alcohols (HAs)** with two or more carbon atoms ($C_{2+}OH$), which are among the most promising clean alternative aviation fuels and support global sustainability objectives[4,5]."

"We strategically deposited **graphitic carbon overlayers (GCLs)** onto a Mo_2C-matrix-supported CuCo alloy catalyst through carburization[20]."

"Additionally, the hydrophobic properties of the GCLs protect the CuCo alloy from oxidation and inhibit the **water-gas shift reaction (WGSR)**, thereby lowering CO_2 selectivity."

"Fig. 1 | Structural characteristics and catalytic performance of the catalysts. ROH = total alcohols. MeOH = methanol. HAs = higher alcohols. CH^{-} = alkanes (including CH_4). $CH^{=}$ = alkenes. RH =

hydrocarbons (CH^{-} + $CH^{=}$). High-value products = ROH + $CH^{=}$. STY = space-time yield based on the CuCo weight. Yield = product yield on a carbon-number basis."

6. Fig 2, a, right, the difference in the two models is not clear

We appreciate your comment regarding figure clarity. We agree that the distinction between the two models in the original figure was not sufficiently clear. Accordingly, Fig. 2a (right) has been revised by improving the visual contrast and adding clearer labels to better distinguish the two models.

Revision in Figure:

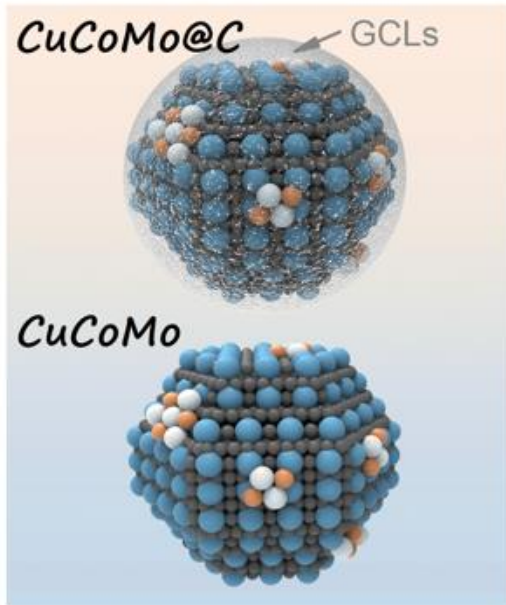

7. L180-185: If it is possible to quantify the Cu or Co surface active sites available, a TON would be beneficial here.

We sincerely thank you for this constructive suggestion. Following your recommendation, we have carried out further analyses of the catalyst surface active sites by combining CO adsorption measurements with complementary O_2–TPO and Raman characterizations. In addition, an interfacial descriptor has been introduced to quantitatively correlate the abundance of coupling sites with the HA selectivity (**Fig. S13**), thereby providing a clearer understanding of intrinsic driving force underlying the structure–performance relationship.

We fully agree that TON/TOF are highly meaningful metrics in catalytic systems where the active sites are singular and well defined. However, in the present catalyst system, the catalytically relevant centers are not simply exposed Cu or Co surface atoms, but rather cooperative sites formed at CuCo-GCLs interfaces, including metal-related sites and defective interfacial carbon sites. Under such circumstances, treating all Cu or Co atoms as equivalent active sites would likely oversimplify the true catalytic nature of this system. Moreover, when apparent turnover metrics are calculated based on CO adsorption, the TOF values of low-carbon-amount samples may be overestimated because

their measured CO uptake is relatively small. Therefore, such values should not be regarded as rigorous indicators of intrinsic activity. Importantly, the calculated HA formation rate exhibits a clear volcano-type dependence (**Fig. 2c**), indicating that the GCLs not only regulate product selectivity, but also determine the efficiency of the effective metal-GCLs interfacial sites.

Therefore, we believe that, compared with TON/TOF metrics, quantitatively correlating the abundance of coupling-active sites with catalytic performance provides a more accurate representation of the true active structure and reaction pathway in this system. Following your suggestion, we have revised this accordingly.

8. Fig 4 – "A plausible reaction" – "A possible reaction"? I'm not sure if we have confirmed it as being plausible?

We thank you for pointing this out. To avoid unnecessary ambiguity in the figure title, we have revised.

Revision in Main Text: "Fig. 4 | Dynamic GCL participation mechanism. Reaction pathway for ethanol formation over CuCoMo@C"

9. L284, L287, charge has units of e, please include.

We thank you for this careful observation. We have revised the manuscript to explicitly include the unit of Bader charge values as **e** at the indicated positions. In addition, several descriptions have been refined to improve the clarity of the presentation.

Revision in Main Text:

"On the CuCoMo surface, co-adsorbed $*CH_2^{CO}/*CH_2^{CO}$ and $*CH_2^{CO}/*CHO^{CO}$ pairs exhibit nearly symmetric charge distributions, with charge differences ($|\Delta q|$) of only 0.007 and 0.01 e, respectively. In contrast, on CuCoMo@C, co-adsorbed GCL-derived $*CH_2^{GCL}$ with CO-derived $*CH_2^{CO}$ and $*CHO^{CO}$ display much larger charge differences of 0.25 and 0.37 e, respectively, forming asymmetrically polarized coupling pairs (Fig. S23b, c)."

10. L289, please improve presentation of \DeltaE_TS to use subscript rather than underscore.

We thank you for this helpful suggestion. We have revised the notation accordingly.

11. L290, Bader charge transfer is normally denoted as \Delta q, not e?

We appreciate your careful reading and comment. We agree that Δq is the more appropriate and commonly used notation for Bader charge transfer/differences. Accordingly, we have revised Δe to

Δq in the manuscript for clarity and consistency.

Revision in Main Text:

"On the CuCoMo surface, co-adsorbed $*CH_2^{CO}/*CH_2^{CO}$ and $*CH_2^{CO}/*CHO^{CO}$ pairs exhibit nearly symmetric charge distributions, with charge differences ($|\Delta q|$) of only 0.007 and 0.01 e, respectively."

12. P12, a title for the conclusions might be valuable, as they appear abruptly.

We thank you for your constructive comment. We note that *Nature Communications* commonly adopts a final Discussion section rather than a separate Conclusions heading. Following your suggestion, we have therefore revised the opening of the final paragraph to provide a clear transition into the concluding discussion and improve the readability of the manuscript.

Revision in Main Text:

Discussion

"By constructing a GCL-coated CuCo alloy supported on a Mo_2C matrix through in situ carburization, alkane selectivity is successfully suppressed to 10%, enabling the co-production of alcohols and olefins with an overall 80% selectivity. In particular, the selectivity toward HAs is increased to nearly 60%, maintaining long-term stability even after prolonged storage. The present results reveal that defective carbon sites at the CuCo-GCL interface are closely correlated with HA formation. The GCLs are not merely passive structural coatings but active catalytic components that dynamically participate in CO hydrogenation. Unlike conventional F–T catalysts, where all intermediates originate exclusively from CO dissociation, the GCLs undergo hydrogenation to generate GCL-derived CH_x species that serve as carbon donors for HA formation. Together with remediation by gaseous CO, this establishes a sustained carbon consumption-replenishment process, partially substituting the CO deoxygenation pathway. Beyond acting as carbon reservoirs, the GCLs also regulate the electronic structure of the C–C coupling intermediates. GCL-derived CH_x species form asymmetrically polarized coupling pairs with CO-derived intermediates, preferentially promoting $*CH_x$ and *CHO coupling while inhibiting the competing hydrocarbon pathway. Consequently, the CO insertion barrier is substantially lowered, thereby promoting oxygenate formation. In addition, the GCLs stabilize the CuCo alloy against oxidation and phase separation while reducing CO_2 formation through their hydrophobic nature, highlighting their multifunctional role in catalyst stabilization and product regulation. This dynamic GCL-involved mechanism provides a solution to the long-standing trade-off between catalytic activity and oxygenate selectivity, extending the traditional understanding of catalyst supports and surface carbon species. These findings demonstrate a general strategy for engineering dynamic catalyst surfaces to steer C–C coupling pathways and enhance oxygenate selectivity in syngas conversion."

13. L515, the data availability should be included here (give link), or in separate data availability statement - currently this seems to be absent.

We thank you for this helpful suggestion. A separate Data availability statement has now been clearly included in the revised manuscript.

Revision in Main Text:

Data availability

"The data supporting the figures presented in this paper are publicly available. Additional supporting data are available from the corresponding authors upon reasonable request. Source data are provided with this paper."

14. Fig S13, this image is not well used and also poorly framed (as in, the image could be zoomed in on the surface configuration of K/CO)

We thank you for pointing this out. We agree that the original presentation was not sufficiently clear. Accordingly, we have revised this figure by improving the framing and zooming in on the surface configuration of the K/CO species to better highlight the relevant structural features.

Revision in Figure:

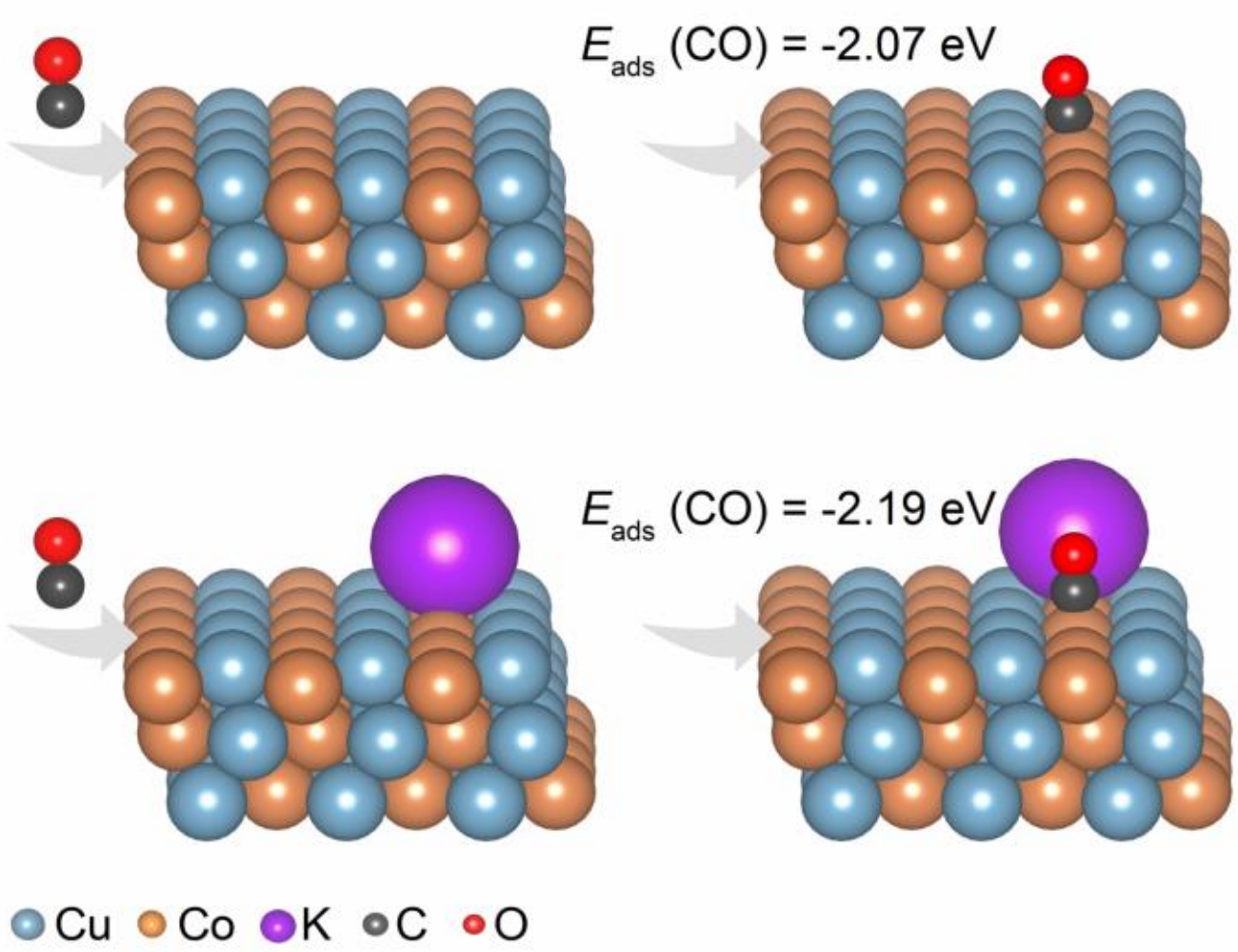

Fig. S24 | CO adsorption energies. (a) CO adsorption energy on the CuCo surface. (b) CO adsorption energy on the K-CuCo surface.

Additional information

Beyond the computational revisions specifically requested, all additional calculations performed using the optimized model have been updated accordingly, and the corresponding changes in the manuscript are provided below.

Revisions in Figures:

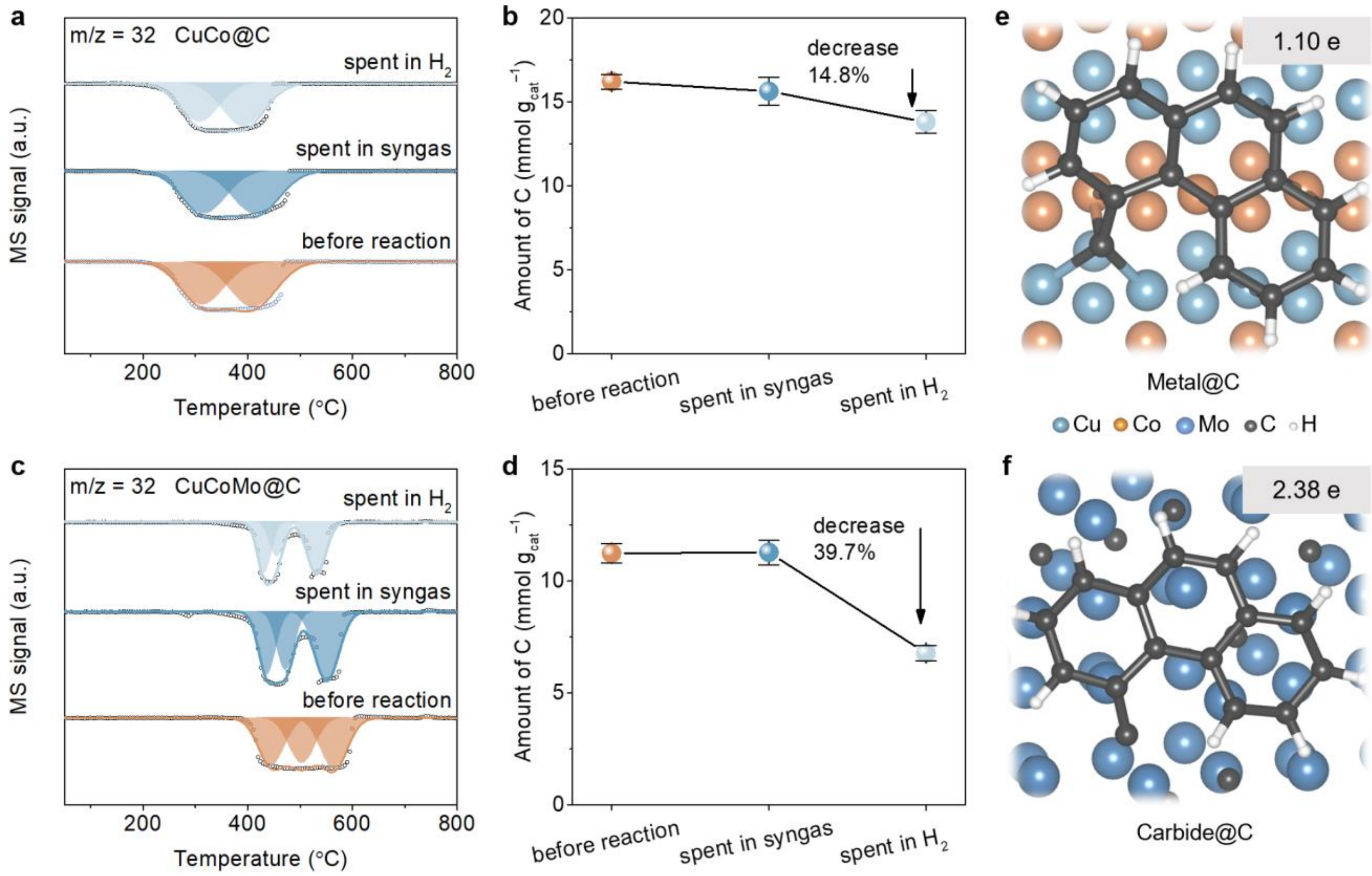

Fig. S14 | Quantitative analysis of carbon overlayer consumption and replenishment. (a) O_2–TPO profiles of the CuCo@C catalyst before reaction, spent in syngas, and spent in H_2 (m/z = 32). (b) Carbon amount of the CuCo@C catalyst at different reaction stages. (c) O_2–TPO profiles of the CuCoMo@C catalyst before reaction, spent in syngas, and spent in H_2 (m/z = 32). (d) Carbon amount of the CuCoMo@C catalyst at different reaction stages. (e) Electron transfer from CuCo to the carbon overlayer. (f) Electron transfer from Mo_2C to the carbon overlayer.

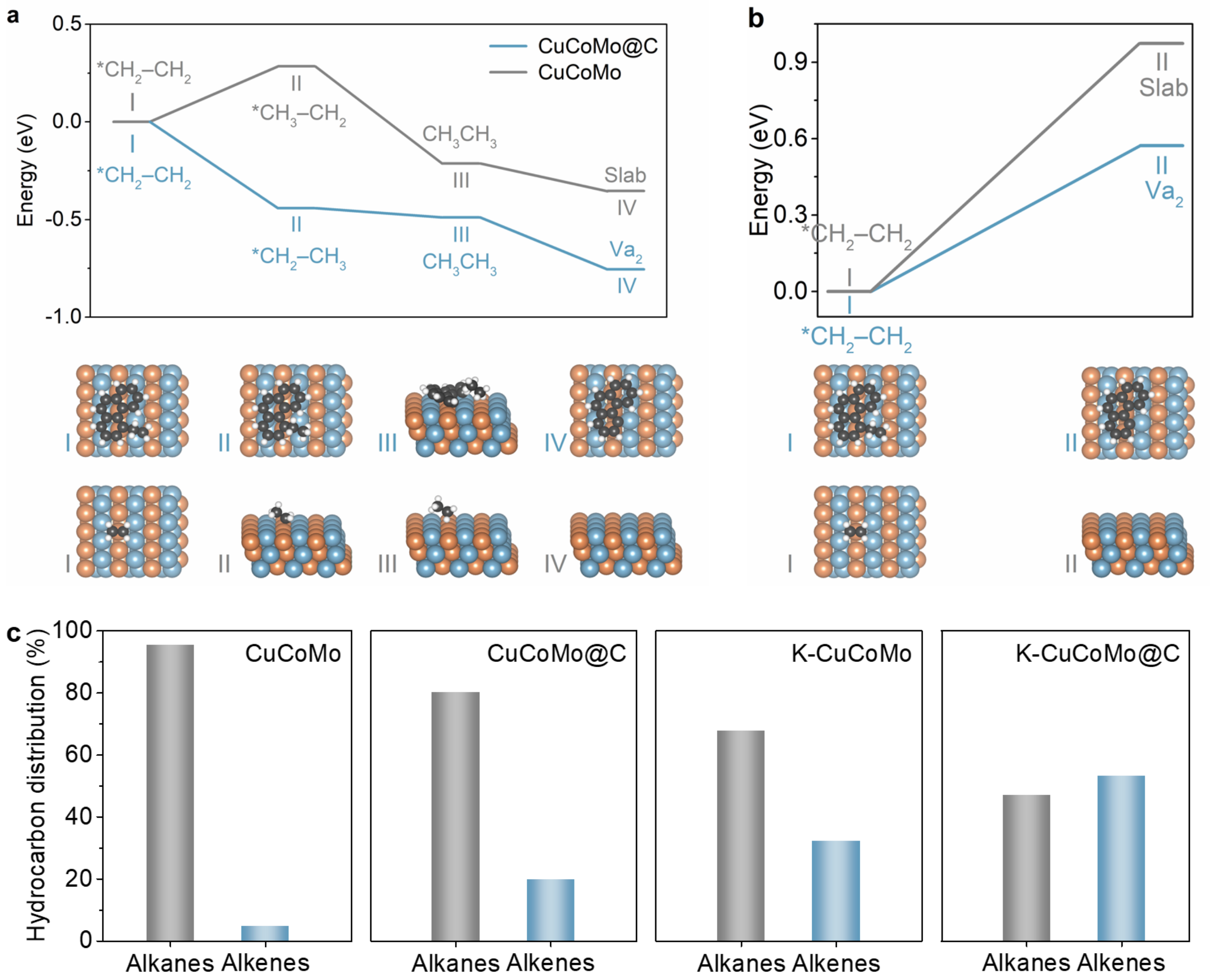

Fig. S22 | Reaction pathways of alkanes and alkenes on CuCoMo and CuCoMo@C surfaces. (a) Reaction pathway of ethylene hydrogenation to ethane. (b) Reaction pathway of ethylene desorption. (c) Hydrocarbon distribution of the catalysts. Reaction conditions: GHSV = 4 L g_{cat}^{-1} h^{-1}, T = 240 °C, P = 3.0 MPa, and H_2/CO = 2.

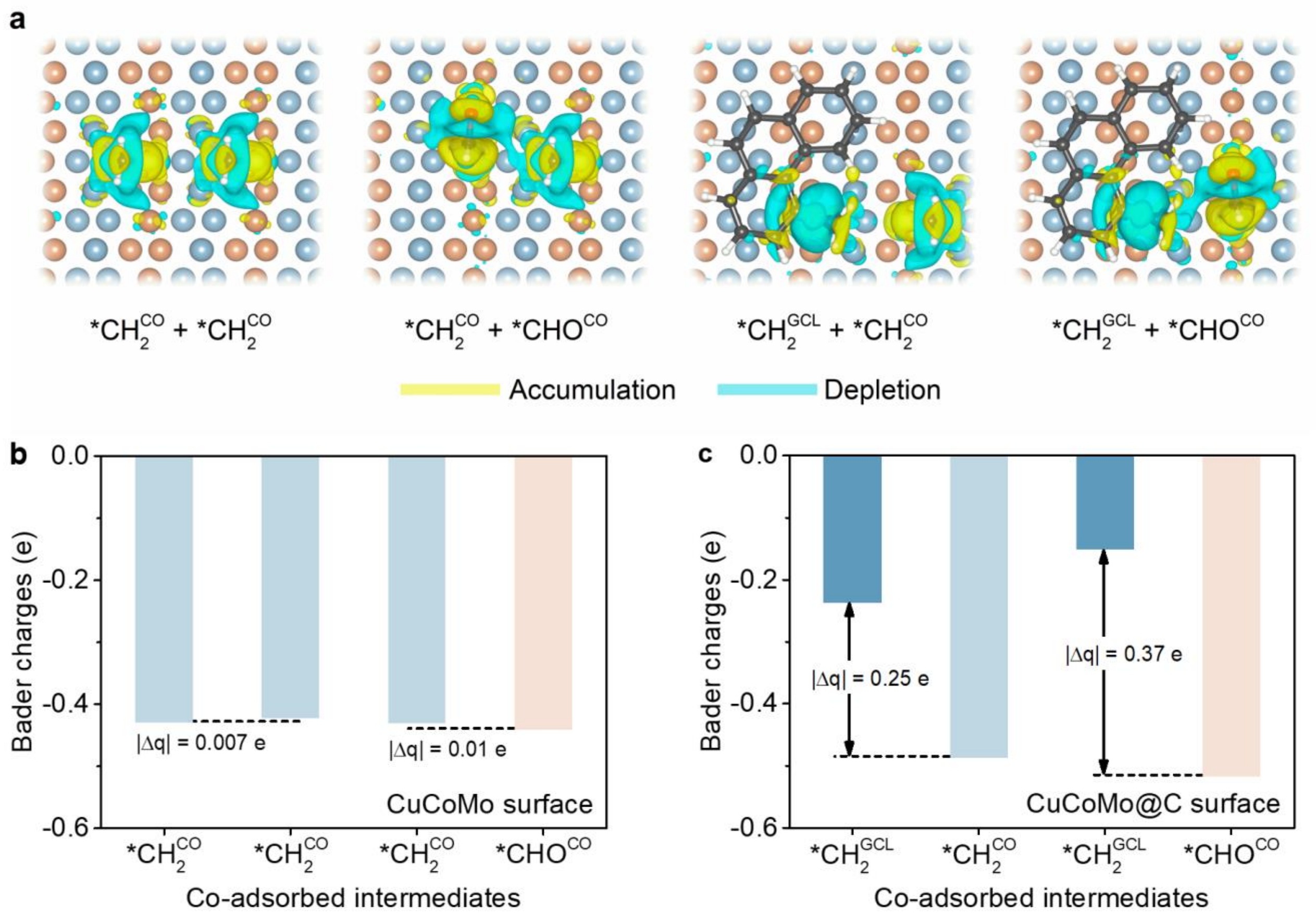

Fig. S23 | Charge distribution analyses of the intermediates. (a) Charge density differences of the co-adsorbed $*CH_2$ and *CHO intermediates on the CuCoMo and CuCoMo@C surfaces. (b) Bader charges of the co-adsorbed $*CH_2$ and *CHO intermediates on the CuCoMo surface. (c) Bader charges of the co-adsorbed $*CH_2$ and *CHO intermediates on the CuCoMo@C surface.

Revisions in Main Text:

On page 8

The enhanced hydrogenation of GCLs supported by the Mo_2C matrix, compared with metallic substrates, can be attributed to the interstitial carbon atoms in Mo_xC[42], which promote the mobility of GCLs (Fig. S15), together with enhanced electron donation from Mo_2C to the GCLs, leading to electron enrichment of interfacial carbon species and increased reactivity toward hydrogenation (**Fig. S14e, f**). Consequently, the GCLs in CuCoMo@C readily generate reactive CH_x intermediates that participate in the reaction cycle.

On page 11

"Compared with the bare metal surface, direct $*CH_2–CH_2$ desorption is thermodynamically more

favorable in the presence of the GCLs, further suppressing alkane formation (**Fig. S22**)."

On page 11

"To elucidate the origin of the preferential $*CH_2$–CHO coupling, charge density difference and Bader charge analyses were performed. Compared with CO-derived $*CH_2^{CO}$, the GCL-derived $*CH_2^{GCL}$ possesses a less electron-rich carbon center owing to charge redistribution at the CuCo-GCL interface (**Fig. S23a**). On the CuCoMo surface, co-adsorbed $*CH_2^{CO}/*CH_2^{CO}$ and $*CH_2^{CO}/*CHO^{CO}$ pairs exhibit nearly symmetric charge distributions, with charge differences ($|\Delta q|$) of only 0.007 and 0.01 e, respectively. In contrast, on CuCoMo@C, co-adsorbed GCL-derived $*CH_2^{GCL}$ with CO-derived $*CH_2^{CO}$ and $*CHO^{CO}$ display much larger charge differences of 0.25 and 0.37 e, respectively, forming asymmetrically polarized coupling pairs (**Fig. S23b, c**). Although both coupling pairs become electronically polarized, the greater charge polarization and the higher intrinsic electrophilicity of the carbonyl carbon render $*CH_2^{GCL}/*CHO^{CO}$ coupling kinetically more favorable than $*CH_2^{GCL}/*CH_2^{CO}$ coupling (Fig. S19c, d). The pronounced asymmetric charge polarization enhances the electronic complementarity between the coupling partners, selectively lowers the CO insertion barrier[45, 46]. Therefore, GCL-derived CH_x species act as carbon donors, partially substituting CH_x species generated through CO deoxygenation, while electronically regulating the C–C coupling process and redirecting CO hydrogenation toward alcohol formation. Furthermore, K promotion slightly strengthens CO adsorption (Fig. S24), increasing the surface coverage of CO-derived intermediates and thereby further facilitating the coupling with GCL-derived CH_x species[47], consistent with the experimentally observed enhancement in HA formation."

Response to Reviewers 2#:

General comment: The revisions and new data provided by the authors are very much appreciated, and many of my previous queries are very well addressed. Thank you.

Response: We sincerely thank you for the positive and encouraging feedback. We greatly appreciate your recognition of our efforts in addressing the previous comments and improving the manuscript.

Questions: There are two minor aspects that I think can just be slightly improved for the reader's benefit:

Q1: In response to my prior comments, you answered QIII by listing computational settings and providing a statement that "These computational settings are standard and have been widely adopted in DFT studies". If they are standard, then it would be appropriate to cite where that standard is reported (noting there is no citation currently). However, for the *k*-point sampling, the provided settings (2×2×1) cannot be "standard" as the choice of this setting is dependent on the unit cell of the studied system. In the current manuscript, the super cell used for modelling is not explicitly stated, so the reader cannot try to make this assessment; but furthermore, *k*-space sampling is a numerically convergent parameter and therefore the validity of this choice should always be presented with a simple graph showing energy as a function of *k*-space sampling. Please provide such a plot in the SI to validate the accuracy of your setting. For completeness, I also note that "electronic convergence criterion" is not specific - what exactly is being converged, please? And I also am inclined to think 0.05 eV/\AA is a relatively inaccurate geometry convergence parameter when reporting local minima/reaction barriers, and it would be valuable for the readers to know how e.g. Fig S21 changes when a more "accurate" (and equally standard) setting of 0.01 eV/\AA is applied.

Response: We sincerely thank you for these constructive and important comments. We agree that the computational settings should be clearly justified and the convergence of the *k*-point sampling should be explicitly demonstrated. We have therefore carefully revised the DFT calculation methods and added additional convergence tests to the Supplementary Information. Our responses to the individual points are provided below.

1. Computational settings and relevant references

We apologize for the lack of appropriate references in our previous response. The statement that the "computational settings are standard and have been widely adopted in DFT studies" was insufficiently supported. We have now added relevant references in the revised manuscript.

2. *k*-point sampling

We agree with you that the choice of *k*-point sampling is dependent on the size of the unit cell and should therefore not be described as a universally "standard" parameter. We have now explicitly stated the unit cell used in the DFT calculation methods in the revised manuscript. In addition, we performed *k*-point convergence tests using different *k*-point meshes. The calculated energies and corresponding CPU times are presented in **Fig. S25**. The calculated energies become essentially stable at the 2×2×1 mesh, with further increasing the *k*-point sampling producing negligible changes. Thus, 2×2×1 provides a reliable and computationally efficient sampling for the present models.

3. Geometry convergence criterion and validation

We apologize for the inaccurate wording in our previous response. The statement "electronic convergence criterion" was not technically precise. The criterion is that energy convergence standard. This has been corrected in the revised manuscript.

We appreciate your concern regarding the 0.05 eV $Å^{-1}$ convergence criterion for locating local minima/reaction barriers. To assess whether this criterion affects the calculated reaction barriers, we performed additional TS calculations using the more stringent convergence criterion of 0.01 eV $Å^{-1}$. For the metal surface model, the calculated barrier for $*CH_2$ and *CHO coupling changes from 1.285 eV with the 0.05 eV $Å^{-1}$ criterion to 1.252 eV with the 0.01 eV $Å^{-1}$ criterion, corresponding to **a difference of only 0.033 eV.** For $*CH_2$ and $*CH_2$ coupling, the corresponding barrier changes from 1.016 eV to 1.027 eV, with **a difference of only 0.011 eV**. The additional convergence tests show that the differences in the calculated energies are negligible and have essentially no impact on the reaction energy profiles and conclusions.

We thank you for pointing out these issues, which have helped us improve the transparency and rigor of the computational methodology.

Action Taken:

1. Revision in Figure

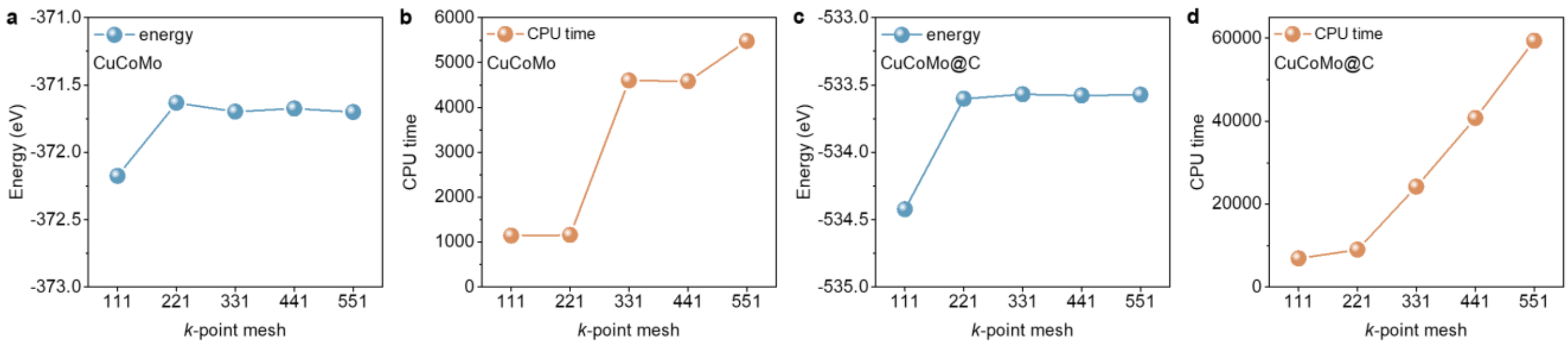

Fig. S25 | *k*-point convergence tests. (a) and (c) Calculated energies as a function of *k*-point sampling for the CuCoMo and CuCoMo@C models, respectively. (b) and (d) Corresponding CPU times required for the calculations.

2. Revisions in Main Text

<u>**On page 15**</u>

DFT calculation methods

Based on the catalyst synthesis and experimental characterizations, the CuCo structure was modeled as a CuCo (111) slab with alternating Cu and Co atomic rows and a Cu:Co ratio of 1:1. The spin-polarized density functional theory (DFT) calculations[47] were performed using the Vienna ab initio simulation package (VASP 5.4.4) based on the projector augmented-wave method with a plane-wave basis set[48,49]. The exchange-correlation potential was treated by using a generalized gradient approximation (GGA) with the Perdew–Burke–Ernzerhof (PBE) parametrization[50]. The three-layer CuCo (111) unit cell was constructed as a rectangular orthogonal cell obtained through a linear transformation of the rhombic lattice vectors, with the atomic configuration unchanged. A vacuum layer of 15 Å was applied to avoid the interaction between periodic images. The plane-wave energy cutoff was set to 450 eV. The calculations used van der Waals correction (DFT–D3). The convergence of the Monkhorst–Pack *k*-point sampling was confirmed by comparing different meshes (Fig. S25), and a 2×2×1 mesh was adopted for structural optimization[51]. The structures were fully relaxed until the maximum force on each atom was less than 0.05 eV $Å^{-1}$, and the energy convergence criterion set to 10^{-5} eV[52].

<u>**On pages 18 and 19**</u>

47. Hohenberg, P. & Kohn, W. Inhomogeneous electron gas. *Phys. Rev.* **136**, 864–871 (1964).
48. Kresse, G. & Furthmüller, J. Efficient iterative schemes for ab initio total-energy calculations using a plane-wave basis set. *Phys. Rev. B* **54**, 11169–11186 (1996).
49. Blöchl, P. E. Projector augmented-wave method. *Phys. Rev. B* **50**, 17953–17979, (1994).

50. Perdew, J. P., Burke, K. & Ernzerhof, M. Generalized gradient approximation made simple. *Phys. Rev. Lett.* **77**, 3865–3868 (1996).
51. Eren, B. et al. Structure of copper–cobalt surface alloys in equilibrium with carbon monoxide gas. *J. Am. Chem. Soc.* **140**, 6575–6581 (2018).
52. Fan, X. et al. Boosted charge and proton transfer over ternary Co/Co_3O_4/CoB for electrochemical nitric oxide reduction to ammonia. *Nat. Commun.* **16**, 4874 (2025).

Q2: I thank the authors for including the Data Availability Statement. However, the provided text does not aid the reader - it would be very valuable to actually state what data is provided, in what formats (i.e., metadata), so the reader can engage with and use the data as effectively as possible. Also consider links/citations to the data if suitable.

Response: We thank you for this helpful suggestion. We agree that the Data Availability Statement (DAS) should provide more specific information about the data available to readers.

In response, we have revised the DAS. In particular, we now specify the DFT-calculated reaction structures provided in the dataset, including the optimized structures of reactants, intermediates, transition states, and products. The structural files are provided in CIF format, and the dataset has been deposited in Figshare with a persistent DOI (https://doi.org/10.6084/m9.figshare.33306180). We have also added the corresponding dataset citation to the reference list to facilitate access and reuse of the data.

Action Taken:

Revisions in Main Text

On page 16

Data availability

The Source Data provided with this article contain the numerical data underlying the figures presented in this study. The structural files corresponding to the DFT-calculated reaction pathways are available at Figshare[53]: https://doi.org/10.6084/m9.figshare.33306180.

On page 19

53. Liu, J. et al. DFT reaction structures of dynamic carbon overlayered CuCo alloy for syngas conversion. *figshare* https://doi.org/10.6084/m9.figshare.33306180 (2026).

Other comment: All other aspects of the revision and response are appreciated, with the additional work completed by the authors commendable.

Response: We sincerely thank you for the positive assessment of our revised manuscript and for recognizing the additional work undertaken in response to the comments. We greatly appreciate your constructive and encouraging feedback, which has helped us further improve the manuscript.

Sincerely,

Youzhu Yuan

On behalf of all authors
